# Correlating Metal Redox Potentials to Co(III)K(I) Catalyst Performances in Carbon Dioxide and Propene Oxide Ring Opening Copolymerization

**DOI:** 10.1002/anie.202308378

**Published:** 2023-08-07

**Authors:** Wouter Lindeboom, Arron C. Deacy, Andreas Phanopoulos, Antoine Buchard, Charlotte K. Williams

**Affiliations:** ^1^ Department Chemistry University of Oxford Chemistry Research Laboratory 12 Mansfield Road Oxford OX1 3TA UK; ^2^ Department of Chemistry Imperial College London Molecular Sciences Research Hub London W12 OBZ UK; ^3^ Department of Chemistry Institute for Sustainability University of Bath Bath BA2 7AY UK

**Keywords:** Carbon Dioxide, Catalysis, Epoxide, Ring Opening Copolymerization, Structure-Activity

## Abstract

Carbon dioxide copolymerization is a front‐runner CO_2_ utilization strategy but its viability depends on improving the catalysis. So far, catalyst structure‐performance correlations have not been straightforward, limiting the ability to predict how to improve both catalytic activity and selectivity. Here, a simple measure of a catalyst ground‐state parameter, metal reduction potential, directly correlates with both polymerization activity and selectivity. It is applied to compare performances of 6 new heterodinuclear Co(III)K(I) catalysts for propene oxide (PO)/CO_2_ ring opening copolymerization (ROCOP) producing poly(propene carbonate) (PPC). The best catalyst shows an excellent turnover frequency of 389 h^−1^ and high PPC selectivity of >99 % (50 °C, 20 bar, 0.025 mol% catalyst). As demonstration of its utility, neither DFT calculations nor ligand Hammett parameter analyses are viable predictors. It is proposed that the cobalt redox potential informs upon the active site electron density with a more electron rich cobalt centre showing better performances. The method may be widely applicable and is recommended to guide future catalyst discovery for other (co)polymerizations and carbon dioxide utilizations.

## Graphical abstract

Carbon dioxide and propene oxide ring opening copolymerization is a leading carbon dioxide utilization technology. Future catalyst development needs better understanding of how to maximize both activity and selectivity. Here, a series of catalysts show a correlation between both their activity and selectivity values with the cobalt reduction potential, providing a useful ‘predictive’ tool for catalyst performances.

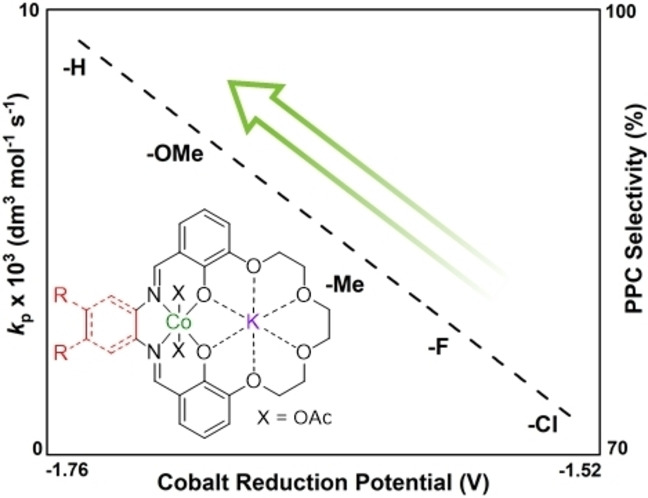

## Introduction

**Figure 1:**
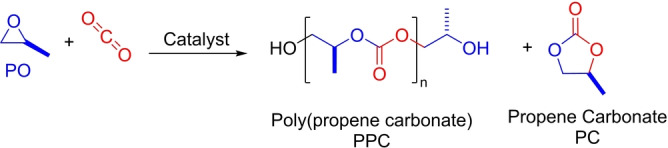
Propene oxide (PO)/carbon dioxide (CO_2_) ring‐opening copolymerization (ROCOP) to synthesize poly(propene carbonate) (PPC) and propene carbonate (PC). Here, the catalyst is applied with 1,2‐cyclohexanediol (CHD) to deliver α,ω‐hydroxyl telechelic polymer chains (catalyst: CHD, 1 : 20, see the Supporting Information for details).

Utilizing CO_2_ to make polymers is a front‐runner carbon dioxide valorisation, an implementable waste recycling strategy as well as locking‐away greenhouse gas emissions.^[1]^ Epoxide/CO_2_ ring opening copolymerization (ROCOP) is truly catalytic, allows up to 48 wt% carbon dioxide uptake, yields valuable products ($1,000–10,000/tonne) and, for the most promising catalysts, can operate at scale (Figure 1).^[2]^ Propene oxide (PO)/CO_2_ ROCOP is particularly important since the product, poly(propene carbonate) (PPC), shows useful properties.^[3]^ Low molar mass, hydroxyl telechelic PPC reduces carbon emissions in manufacturing of home insulation, automotive and household foams, in addition to having desirable properties as a surfactant.^[4]^ Life‐cycle assessments show a ‘triple win’ where every molecule of carbon dioxide utilized saves two more by avoiding PO consumption.^[5]^ Higher molar mass PPC improves properties of bio‐derived plastics, like PLA, and as a component in block polymers furnishes toughened plastics and pressure sensitive adhesives.^[6]^ PO is a commodity chemical already used at scale in polymer manufacturing, but its carbon dioxide copolymerization is challenging because the polymer (PPC) is the kinetic product and, in many cases, the thermodynamic product, propene carbonate (PC), shows an equivalent, or only marginally higher, barrier to formation.^[7]^ The best polymerization catalysts must, therefore, combine both high rates and selectivity, under conditions suitable for larger‐scale use. There are some excellent examples including [Co(III)(salen)X]/PPNX, co‐catalyst tethered [Co(salen‐X)X], trinuclear [Co_3_{(salen)_3_Ph}X_3_] and polymer decorated, PMMA‐[Al(III)(porphyrin)X]/PPNX, catalyst systems (where X=halide or 2,4‐dinitrophenolate, PPN=bis(triphenylphosphine)iminium, salen=various tetradentate salicylimine ligands).^[8]^

Generally, in catalysis, understanding structure–activity relationships is of central importance, from single atom heterogeneous catalysts, to carbon dioxide reduction nanocatalysts, to control over tacticity in alkene polymerization catalysts.^[9]^ It underpins the ethos of homogeneous polymerization catalyst design, but because such catalysts are applied at very low loadings, deconvoluting effects is very demanding. Even when catalysts are studied under relevant conditions, in many cases structure‐performance relationships are elusive. For example, a recent systematic investigation of heterodinuclear catalysts comprising M(II)Mg(II), where M=Cr(II)−Zn(II), for epoxide/carbon dioxide ROCOP failed to show any correlation between activity and metal ionic radius data, published M^2+^ Lewis acidity values or ligand Hammett parameters.^[10]^ Similarly, efficient tetranuclear catalysts of the form Ln_3_(III)/Co(II) where Ln(III)=La‐Dy, for cyclohexene oxide/carbon dioxide ROCOP, failed to show any correlation between activity and a series of different catalyst physical properties.^[11]^ Perhaps the most successful design parameter has been that di‐ (or multi‐)nuclear metal catalysts tend to outperform mononuclear analogues.[^8k^, ^12^] This understanding has derived from the efforts of many researchers comparing different types of dimeric or dinuclear catalysts with mononuclear analogues.[^8k^, ^12^] There has also been attempts to correlate zinc glutarate heterogeneous catalysts’ performances with Zn−Zn separations, although care must be taken since these catalyst surfaces and crystallinities are highly variable.^[13]^ Our team showed that heterodinuclear catalysts can outperform homodinuclear analogues.[^10^, ^14^] Kinetic investigations revealed that synergic Co(II)Mg(II) catalysts perform better because the Co(II) reduces the transition state enthalpy barrier and Mg(II) reduces the transition state entropy barrier.^[14b]^ Currently, aside from targeting (hetero)dinuclear catalysts, there is insufficient ligand structure–activity insight. Rather catalyst performances tend to be (post)rationalized using DFT calculations to elucidate mechanisms.^[15]^ An excellent investigation, from Nozaki and co‐workers, showed a direct relationship between activity and computed difference between M‐carbonate and M‐epoxide bond dissociation energies using (planar, tetradentate ligand)MCl/PPNCl catalyst systems, where M=Cr(III), Co(III).^[8g]^ The discovery of boron/ammonium catalyst systems has also been important particularly for CHO/CO_2_ ROCOP, where the ‘C_5_‐tether’ resulted in higher activity than other linkers.^[16]^ Nonetheless, there was no correlation between activity and boron centre Lewis acidity, or any other measurable parameter.^[16]^ To develop better catalysts both for this carbon dioxide copolymerization and other carbon dioxide utilizations, we need better understanding and predictive measures for catalyst structure–activity and selectivity relationships.

## Results and Discussion

**Figure 2:**
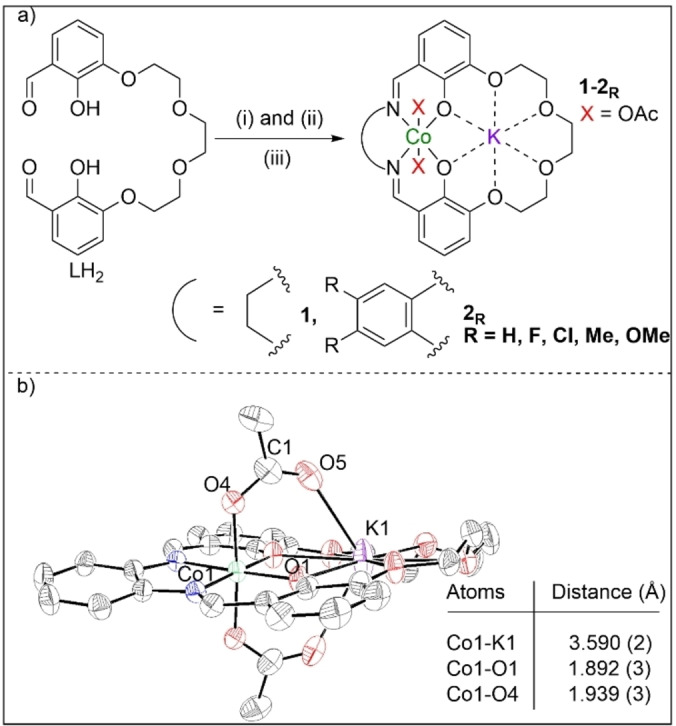
a) Synthesis of complexes **1**–**2_R_**. (i) KOAc, Co(OAc)_2_, MeCN, 25 °C, 30 min. (ii) Diamine, MeCN, 25 °C, 16 h. (iii) AcOH, air, MeCN, 48 h. **2_OMe_**: (i) Ba(ClO_4_)_2_, MeCN, 25 °C; then diamine, 24 h. (ii) Guanidinium sulfate, H_2_O/CHCl_3_, 25 °C, 16 h. (iii) KOAc, Co(OAc)_2_, MeCN, 25 °C, 16 h.(iv) AcOH, air, MeCN, 48 h. b) Molecular structure of **2_H_**, determined by X‐ray diffraction (thermal ellipsoids, 50 % probability) and selected bond lengths (Table S1 and Figure S1).

Here, a series of heterodinuclear catalysts, featuring a macrocyclic ligand coordinating to Co(III)/K(I), are investigated for PO/CO_2_ ROCOP (Figure 2).^[17]^

**Figure 3:**
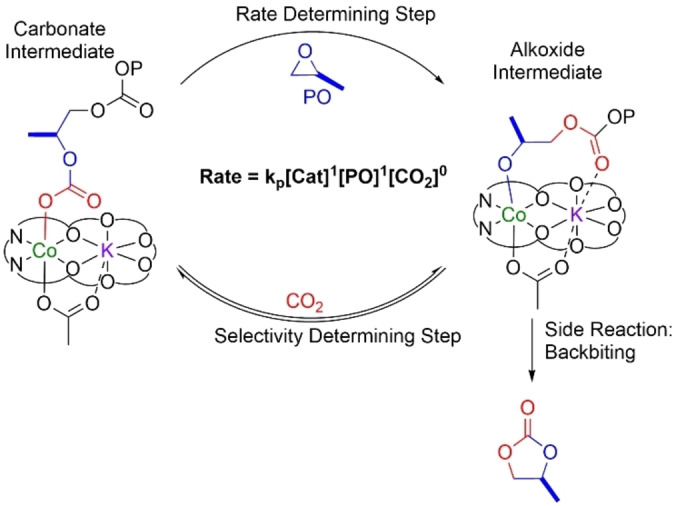
Dinuclear cobaltate mechanism for the PO/CO_2_ ROCOP with rate‐ and selectivity‐limiting steps identified.

Recently, catalyst **1** showed competitive activity and selectivity with leading Co(III)/PPNCl catalyst systems yet it operated without additives and efficiently with alcohols (relevant to delivering a wide range of products).^[17a]^ Its rate law and computed catalytic cycle (DFT) were rationalised by a dinuclear metallate mechanism, in which the rate determining step involves Co(III) coordinating and activating the propene oxide to attack by a transient K(I)‐carbonate nucleophile (Figure 3).^[17c]^ To investigate catalyst structure‐performance relationships, here, systematic modifications to the ligand imine substituents were targeted since these should allow for modification of the electronic character of the Co(III) active site. Catalysts **1**–**2_R_** were synthesized by reacting the dialdehyde ligand precursor (LH_2_) with the appropriate diamine linker, an equivalent of potassium(I) acetate and cobalt(II) acetate, followed by addition of excess acetic acid (Figure 2a, Supporting Information for experimental details; note **2_OMe_** was prepared differently).^[17a]^ By varying the diamines, 6 Co(III)K(I) complexes were isolated, differing only by the ‘linker’: ethylene (**1**), phenylene (**2_H_**) and substituted phenylene (**2_F_**_, **Cl**, **Me**, **OMe**_). The catalysts are all heterodinuclear complexes: Co(III) is always coordinated by the ligands’ diphenolate diamine moieties (*O*,*N*,*N*,*O*) and K(I) by the diphenolate‐tetraether moieties. All complexes were fully characterized using NMR spectroscopies (Figure S2–S26), IR spectroscopy (Figure S27), cyclic voltammetry (CV) (Figure S28, Table S2), elemental analysis and, where possible, by single crystal X‐ray diffraction (Table S1, Figure 2 and S1). Typical ^1^H NMR data confirm the complex formation with the LH_2_ aldehyde resonance being completely consumed (9.94 ppm) and a single imine resonance forming (8.40–7.51 ppm). The phenol proton resonances (∼10.86 ppm) disappear and new acetate and linker resonances are observed (Figure S20–S26). All the ^13^C{^1^H} NMR spectra are assigned using COSY, HSQC and HMBC and show the expected acetate (∼180 ppm), imine (162–155 ppm) and linker resonances (Figure S2–S26). All complexes show asymmetric and symmetric acetate stretches in the IR spectra (Figure S27). Single crystals of **2_H_**, characterized by XRD, confirm the heterodinuclear speciation and ligand coordination modes (Figure 2b and Table S1). The catalyst has *C_2_* symmetry (Co_1_‐K_1_ axis) which renders the acetate ligands equivalent and both adopt bridging, i.e. *κ*_2_, coordination modes. In contrast, **1** has a structure featuring both bridging and terminal acetate ligands (Figure S1).^[17a]^ These differences may arise from the greater ‘phenylene’ linker rigidity, supported by a shorter Co−K distance in **2_H_** (3.5904(17) Å) than in **1** (3.6979(5) Å).

**Figure 4:**
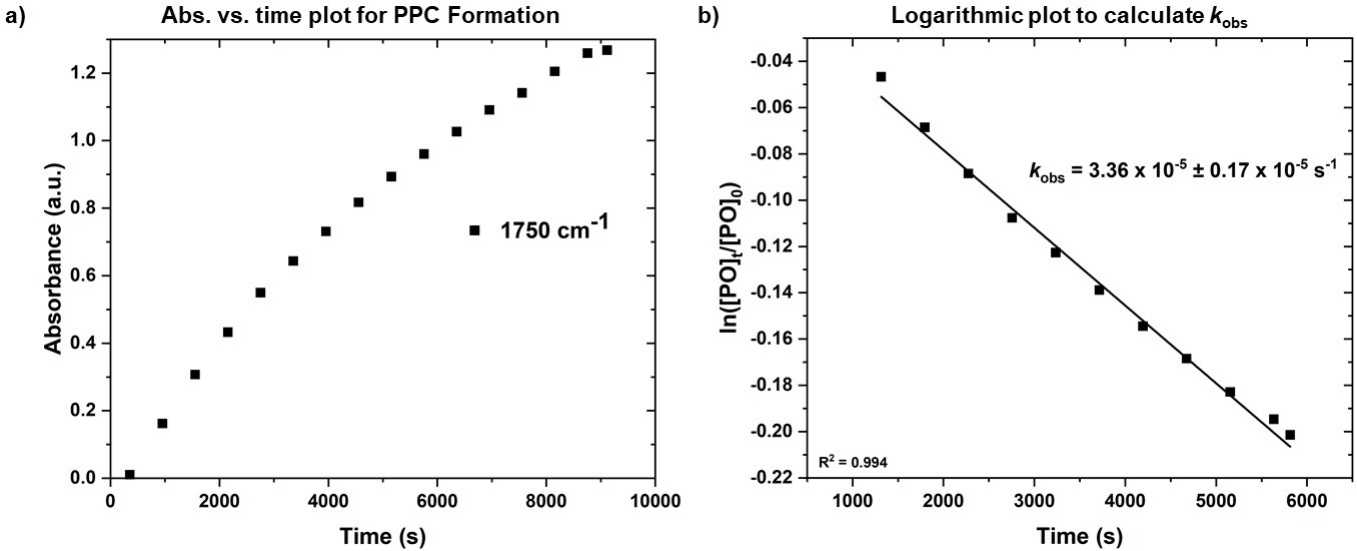
Exemplar polymerization kinetics data for catalyst **2_H_**. a) Plot showing PPC formation, analysed by changes to the absorbance at 1750 cm^−1^ (carbonyl stretch) versus time. b) Semi‐logarithmic plot of PO concentration vs. time, used to determine *k*_obs_ (Figs. S29–S32).

Catalysts **1**–**2_R_** were each tested for PO/CO_2_ copolymerization, at 50 °C, using neat PO (14 M, 6 mL), 0.025 mol % (i.e. 1 : 4000, catalyst:PO) catalyst loading, 20 bar CO_2_ pressure and with 0.5 mol % 1,2‐cyclohexanediol (CHD, 20 equiv. vs catalyst) (Figure 1, Table 1). These conditions are demanding but relevant to production of PPC polyols where the excess diol controls molar mass; notably under these conditions other leading catalysts can show diminished activity.[^11a^, ^17a^, ^18^] Using a Parr reactor fitted with a DiComp sentinel probe, attached to an ATR‐IR spectrometer, allowed for continual monitoring of absorptions, and hence concentrations, of both polymer (PPC, 1750 cm^−1^) and cyclic carbonate by‐product (PC, 1810 cm^−1^). All catalysts showed good performances with high selectivity for PPC formation, quantitative CO_2_ uptake (no ether linkages), and high selectivity for hydroxyl telechelic PPC (Figure 4 and S29–S39).^[17a]^ The PPC samples all showed molar mass values, consistent with theoretical values, in the target range for polyols, with monomodal, narrow dispersity distributions (*Ð*<1.20).^[17a]^

**Table 1:** CO_2_/PO ROCOP data for catalysts **1** and **2_H_**_, **Me**, **OMe**, **F**, **Cl**_.^[a]^

Cat.	Productivity TON^[b]^	Activity TO_FPPC_ (h^−1^)^[c]^	Polym. rate coefficient *k*p *10^3[d]^ (dm^3^ mol^−1^ s^M−>1^)	Select. PPC^[e]^ (%)	PPC molar mass^[f]^ *M*n [*Ð*] (g mol^−1^)
**1** ^[g]^	1360±68	333±17	11.20±0.56	98	5300 [1.08]
**2_H_**	973±49	389±19	9.40±0.47	>99	4000 [1.06]
**2_Me_**	1402±70	101±6	4.40±0.22	85	5500 [1.15]
**2_OMe_**	1494±75	191±10	7.25±0.36	96	8900 [1.11]
**2_F_**	1196±60	73±4	1.78±0.09	80	4400 [1.11]
**2_Cl_**	962±48	62±3	1.44±0.07	75	4200 [1.13]

[a] Catalyst (0.025 mol %, 1 equiv., 3.5 mM), 1,2‐cyclohexanediol (0.5 mol %, 20 equiv., 70 mM), PO (6 mL, 4000 equiv. 14 M), 20 bar CO_2_, 50 °C. Errors from triple repeat experiments. All catalysts >99 % CO_2_ selectivity by ^1^H NMR spectroscopy (Figure S34, Table S3). [b] Turnover number (TON)=moles of PO consumed/moles catalyst, PO conversion determined from the ^1^H NMR spectrum from the integrals for PPC (4.92 ppm) and PC (4.77 ppm), using mesitylene as an internal standard. [c] Turnover frequency of PPC (TOF_PPC_)=(TON*PPC selectivity)/time (h). [d] *k_p_* =*k_obs_*/[cat]^1^; *k_obs_* determined from plots of ln[PO]_*t*_/[PO]_0_ vs time (Figure 4 and S29–S33). [e] PPC selectivity determined from the ^1^H NMR spectrum of the crude product by comparing integrals for PPC (4.92 ppm) vs. PC (4.77 ppm), errors ±1 %. [f] Determined by GPC, in THF, calibrated with narrow‐*M*_n_ polystyrene standards; dispersity values in parentheses (Figure S35–S39). [g] Data consistent with previous reports for catalyst **1**.^[17a]^

Within the series of catalysts there are significant differences in performances: the activity values, as assessed by turn‐over‐frequency (TOF), vary from 60–390 h^−1^ and the PPC selectivity varies from 75–99 %. For each catalyst, the selectivity was consistent throughout the reaction and independent of conversion, thereby suggesting that the different catalysts’ structures are influencing performances (Figure 4 and S29–S33). The rate law for **1** is first order in both PO and catalyst concentrations but independent of carbon dioxide pressure (Figure 3).^[17a]^ It is underpinned by the dinuclear cobaltate mechanism supported by kinetics and DFT calculations (Figure 3).^[17c]^ From the experimental data, the rate determining step could be epoxide coordination and/or ring‐opening by the carbonate intermediate but the computed mechanism suggests that ring‐opening is rate limiting.

The new catalysts are all assumed to operate by similar rate laws and all showed the expected first orders in epoxide concentrations (Figure 4). To properly understand differences in rates between the catalysts, both the catalytic turn‐over frequency value (TOF) and the initial rate constants (5–20 % conversion range) were determined. In other work, it was shown that the initial polymerization rates, determined in a similar manner, were within error equivalent to integrated rates (20–80 % conversion) giving confidence that they allow for comparisons of propagation rate differences within the series.^[14a]^ The comparison between propagation rate constants is significant since it prevents any misleading point kinetic values from distorting trends and measurements are conducted prior to higher conversions where diffusion limitations which may limit performances.

For each catalyst, the absorbance versus time data was converted into semi‐logarithmic [PO] vs. time plots by independent calibration of the starting and final PO concentrations (Figure 4). The pseudo first order rate coefficients, *k*_obs, PPC_, were converted into propagation rate coefficients, *k*_p, PPC_ by accounting for the catalyst concentration, using the rate law. The trends in propagation rate constants matched those in point kinetic activity (i.e. TOF), with values from 9.40±0.47 to 1.44±0.07 mM^−1^ s^−1^ (Table 1). The catalyst featuring the rigid phenylene linker, **2_H_**, was the most active and selective, showing a TOF of 389 h^−1^, *k*_p, PPC_ of 9.40±0.47 mM^−1^ s^−1^ and >99 % PPC selectivity. The success of the catalyst featuring the phenylene linker was particularly promising since it allowed systematic comparison of catalysts featuring substituted phenylene linkers, i.e. complexes **2_H_**_, **Me**, **OMe**, **F**, **Cl**_. The phenylene substituents were all installed opposite the two N atoms, such that each is both *para*‐ and *meta*‐ to an imine group. All substituents are selected to limit the (negative) steric effects at the Co(III) active site, although any dispersion effect differences are not measured in this work. The methyl substituted catalyst, **2_Me_**, showed a three‐fold reduction in rate, TOF=111 h^−1^ (*k*_p, PPC_=4.40±0.22 mM^−1^ s^−1^) and lower selectivity (85 % PPC) than **2_H_**. The methoxy substituted catalyst, **2_OMe_**, showed TOF=191 h^−1^ (*k*_p, PPC_=7.25±0.36 mM^−1^ s^−1^) and selectivity of 96 % but was also less effective than **2_H_**. Applying fluorine substituents **2_F_** significantly slowed the rate, TOF=73 h^−1^ (*k*_p, PPC_=1.78±0.09 mM^−1^ s^−1^) and reduced selectivity, 80 %, compared with **2_H_**. The chlorine substituted catalyst, **2_Cl_**, showed the lowest activity and selectivity, TOF=62 h^−1^ (k_p_=1.44±0.07 mM^−1^ s^−1^) and 75 % PPC selectivity. The best catalyst remained the phenylene variant, **2_H_**, nonetheless, the substituents clearly influence both rate and selectivity.

**Figure 5:**
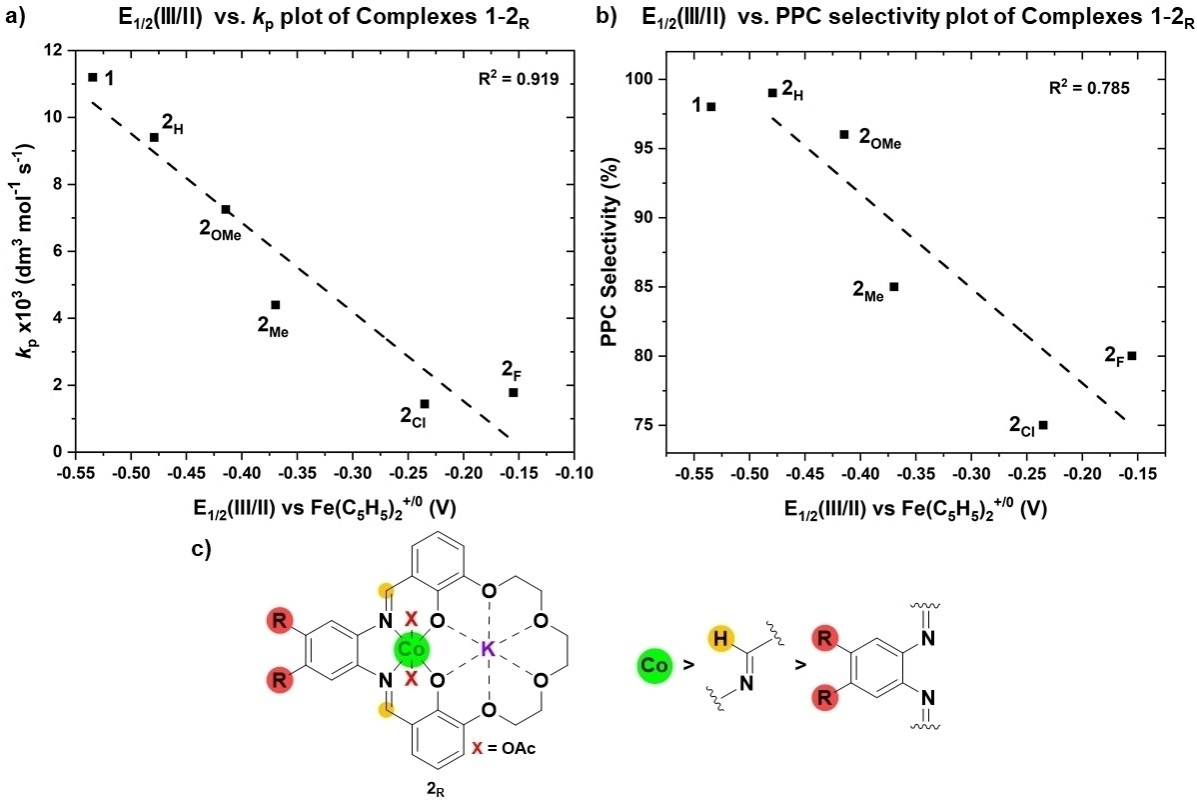
Linear correlations between catalyst activity (a) and selectivity (b) with Co(III) reduction potential values. a) Plot of *E*_1/2_(III/II) vs. *k*_p_. b) Plot of *E*_1/2_(III/II) vs. PPC selectivity. c) Schematic diagram showing ‘detectors’ for rate and selectivity with the accuracy of the correlation, red=ligand substituents assessed by Hammet parameter, yellow=imine chemical shifts as assessed by ^1^H NMR spectroscopy, green=reduction potential of the Co centre as assessed by cyclic voltammetry (Figure S40–S42 for correlations with other substituents).

To better understand the structure‐performance relationships, it is important to characterize the active site and specifically the Co(III) since it coordinates both alkoxide and carbonate intermediates, and the epoxide in the transition state, in the catalytic cycle. It was proposed that the Co(III) reduction potentials might offer information about the relative electron density at the active site. To test this notion, cyclic voltammetry experiments were conducted for each of the catalysts under an inert atmosphere (N_2_, glove box), at ambient temperature and in acetonitrile solutions. Experiments applied a fixed catalyst concentration (1.6 mM), tested in an inert electrolyte (tetrabutylammonium hexafluorophosphate, 0.1 M) and at fixed scan rate (0.1 V/s) from −1.5 to 1.5 V (vs. ferrocene, see SI). All complexes showed two single electron reductions, corresponding to Co(III/II) and Co(II/I), at values consistent with those reported for other Co(III) complexes; in most cases these reductions are reversible (Figure S28, Table S2).^[19]^ Interestingly, a plot of the propagation rate constants (*k_p_*) *vs*. the reduction potentials, *E*_1/2_(Co(III/II)) or E_red_(Co(II/I)), showed linear fits and a direct correlation (Figure 5a, b, S40). Even more surprisingly, plotting the selectivity for PPC vs. the same reduction potentials (either Co(III) or Co(II)) also showed linear fits and direct correlations.

Within the ‘phenylene’ series of catalysts, **2_H_** has the most negative reduction potential, i.e. the greatest electron density at Co(III) of the **2_R_** series and it performed best. Even better, the previously reported ethylene linked catalyst, **1**, showed the lowest absolute reduction potential, i.e. the highest electron density at Co(III), and the highest overall activity and selectivity. These measurements clearly show that increasing the Co(III) electron density (as inferred from more negative reduction potentials) correlates with higher copolymerization catalytic activity and selectivity. Thus, the Co(III) redox measurements provide a simple predictor, based on the ground‐state catalyst structure, that informs upon two of the critical polymerization performance metrics. It must be emphasized that current understanding of ‘catalyst design’ does not predict that catalysts with high activity should also show high selectivity.^[8]^ Further, these findings are especially surprising given the prior failures to correlate activity or selectivity to experimentally measurable catalyst parameters or properties.[^10^, ^11^, ^16^]

With the value of Co(III) electrochemistry as ‘probe’ evident, other catalyst structural parameters were also examined to understand whether these catalysts were unusually ‘predictable’. Plots of catalytic activity, or selectivity, against the imine protons’ chemical shifts (^1^H NMR spectra) also showed a linear relationship but with a much weaker correlation (Figure S41). The greater separation of ‘probe’ from active site and the insensitivity of NMR spectroscopy likely limits the utility of this method. Plots of catalyst performance metrics against the phenylene substituent Hammett parameters, σ, accounting for both meta‐ and para‐ effects, failed to show any correlation (Figure S42). The possible use of Guttman Beckett, or related NMR spectroscopy experiments, to ‘measure’ active site Lewis acidity (by binding of epoxides or other ‘model’ substrates) was not feasible due to the tendency for probe molecules to preferentially coordinate to K(I). Both DFT and experimental investigations revealed that attempts to coordinate epoxides form a non‐productive K(I)‐epoxide intermediate.^[17c]^ This intermediate, although the most stable adduct, shows a much higher catalytic pathway than the (higher‐energy) productive Co(III)‐epoxide species.^[17c]^ The same issue prevents investigations of epoxide binding by X‐ray crystallography. These difficulties using other simple experimental measurements to predict catalyst performances further highlight the benefit of using Co(III) reduction potentials to inform upon active site electronics.

### Discussion

**Figure 6:**
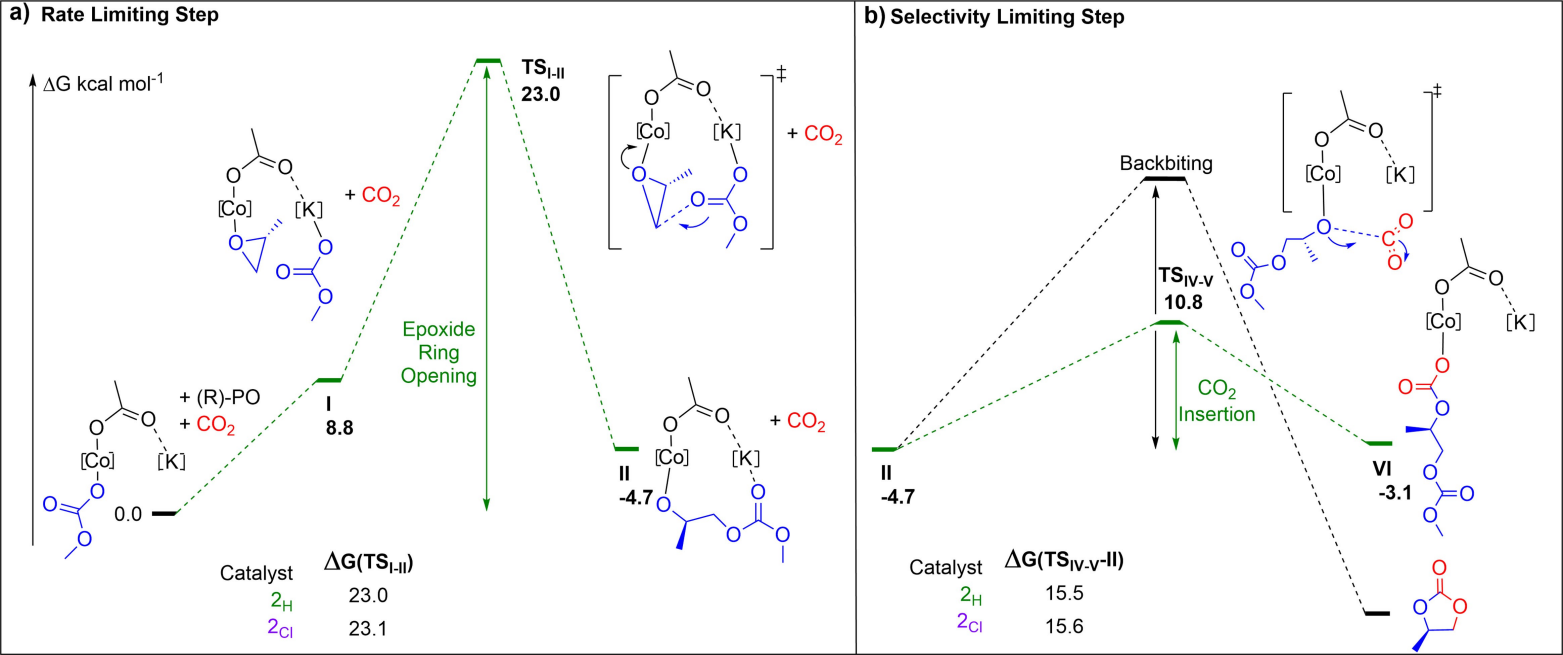
The PO/CO2 ROCOP mechanism highlighting rate‐ and selectivity‐limiting steps. a) The rate‐determining step is the epoxide ring‐opening with the calculated barriers for **2_H_** and **2_Cl_** (ligands are omitted for clarity, Figure S44–S56, Table S4–S9). b) The selectivity‐determining step in which the alkoxide intermediate (II) forms either PPC (CO_2_ insertion) or PC (backbiting), with calculated barriers for **2_H_** and **2_Cl_**_._ N.B. For clarity some intermediates are not illustrated in this step, the numbering follows the full mechanism (Figure S44–S56, Table S4–S9).

To rationalise the structure performance correlations, it is appropriate to examine the polymerization rate law and mechanism. The polymerization rate law is first order in both epoxide and catalyst concentrations, with the proposed rate determining transition step involving attack upon a Co(III)‐epoxide intermediate (**I**) by a K(I)‐carbonate species (TS_I‐II_, Figure 6a).^[17c]^

The attack forms a new Co(III)‐alkoxide intermediate, **II**. In subsequent steps, **II** inserts carbon dioxide, via an equilibrium process, to (re)form the carbonate intermediate **VI** which is equivalent, except for being one repeat unit longer, to the initial reference intermediate (0.0), capable of initiating the next catalytic cycle (Figure 6b). The rate differences observed experimentally between these catalysts are rationalised by more electron rich Co(III)/K(I) catalysts, like **1** and **2_H_**, being more reactive towards epoxide ring‐opening and insertion reactions. It is proposed that these electron rich catalysts form slightly destabilized epoxide coordination intermediates (**I**) as a consequence of reduced Lewis acidity of the Co(III) centres. These catalysts should also show lower barriers to epoxide ring‐opening—i.e. catalysis is likely accelerated by reducing the transition state energy compared with less electron rich catalysts. In addition, more electron rich Co(III) catalysts are expected to form destabilized Co(III)‐alkoxide intermediates (**II**), which show carbon dioxide insertion equilibria driven towards the Co(III)‐carbonate intermediate (**VI**). Even minor shifts to the CO_2_ insertion equilibria could significantly accelerate rates by increasing the true concentration of ‘catalyst’, i.e. carbonate intermediate **I**, available in the rate determining step. To understand whether DFT calculations could be used to detect any differences between the catalysts, the rate determining PO insertion step as well as the selectivity determining CO_2_ insertion step were investigated computationally for each catalyst (see Supporting Information for details). Here, the experimental catalytic performance extremes are discussed, i.e. the fastest and most selective **2_H_** compared against the slowest and least selective **2_Cl_** (Figure 6). Although the DFT calculations predicted rate differences within the family of catalysts comparable to those observed experimentally (see Supporting Information for full analysis), they failed to replicate any trend when comparing a series of calculated kinetic or thermodynamic parameters against experimentally measured rates. Additionally, there was no obvious correlation between any catalyst ground‐state computed parameter, including frontier energy levels, bond‐lengths or bond strengths (Wiberg Bonding Indices), ionization potentials or charges on Co(III). Overall, at this level of theory DFT could not be used to explain the observed catalyst activity trends. The failure of easily applicable and common computational methods to distinguish between catalysts further highlights the significance of using Co(III) reduction potentials to predict polymerization activity.

The catalytic selectivity for PPC (polymer) vs. PC (by‐product) is as important as maximizing activity, not least because large‐scale removal of high boiling cyclic carbonates is energy intensive. The selectivity depends upon the reactions available to the alkoxide intermediate **II**. Two pathways are possible: either reaction with carbon dioxide, an equilibrium process, to form the carbonate intermediate **VI**, enabling subsequent PPC propagation. Or the backbiting upon its own polycarbonate chain to form propene carbonate (the five‐membered ring) and a chain‐shortened alkoxide intermediate.^[17c]^ The extent of the carbon dioxide equilibrium dictates which of the pathways are accessed (Figure 6b).^[17c]^ It was observed that higher carbon dioxide pressures and lower temperatures favour carbon dioxide insertion and polymer propagation.^[17c]^ These catalysts clearly show different PPC selectivity values under identical experimental conditions (i.e. temperature and pressure), suggesting their structures, and electronic properties also influence the carbon dioxide insertion equilibrium.

To understand whether the different polymerization selectivity values arose from any changes to the cyclic carbonate reaction barriers, the experimental barrier to PC formation was measured.^[17c]^ It was possible to measure only the barrier to backbiting by reacting the hydroxyl‐telechelic polymer, PPC, with each of the catalysts, over a range of temperatures, and measuring the different rates of cyclic carbonate PC formation. The experiments were undertaken using the most (**1**) and least (**2_Cl_**) selective catalysts in forward polymerization (Figure S43). Both catalysts show the same PC formation barriers, within experimental error, with values being **1**=19.5±1.0 kcal mol^−1^ and **2_Cl_** =19.9±1.2 kcal mol^−1^. Thus, the polymerization selectivity is not governed by changes to the propene carbonate formation barriers. Rather selectivity is governed by the local concentration of the alkoxide intermediate **II** and the relative position of the carbon dioxide insertion equilibrium. Electron rich catalysts, like **1**, have carbon dioxide insertion equilibria driven towards the carbonate intermediate. This results in both higher selectivity and lower backbiting rates since the local alkoxide intermediate concentration is lower. The position/extent of the carbon dioxide insertion equilibrium also influences the rate of polymerization as discussed earlier (Figure 6). Thus, despite the by‐product formation barriers being accessible under reaction conditions, differing extents of carbon dioxide insertion control product rates and selectivity.

The goals of catalyst design are to increase activity and selectivity and, as such, understanding structure‐performance relationships is essential for rational catalyst design. Unfortunately, many homogeneous polymerizations lack clear correlations between catalyst structures/physical parameters and performances. Here, a useful method to directly measure the relative electron density at the active site, in this case Co(III), is described allowing for predictions of both activity and selectivity. Using electrochemical methods to characterize the catalyst, even when the catalysis is not itself a redox reaction, shows great promise as a measure for active site electron density and as a predictor for performances. The method is attractive since cyclic voltammetry experiments are inexpensive, easy to set‐up and work under inert conditions—there is no reliance on sophisticated operando spectroscopies. The technique could be used to test and accelerate future catalyst development. Electrochemical reduction potentials can be easily determined for other metals, main group elements or even for some ‘organic’ active sites, thus, in future these measurements are recommended to guide other catalyst structure‐performance relationships. A different application of active redox potential has been to assess catalyst lifetime, for example where thermally driven metal reduction deactivates the catalyst. Several researchers already applied Co(III/II) redox potential measurements, with Co(III)(salen)X/PPNX and with Co(III)(pophyrin)X/PPNX catalysts, to rationalise catalyst lifetimes and decomposition routes to inactive Co(II) species.^[20]^

Considering the generality of the methodology, many other polymerization catalyses also require labile metal alkoxide/carboxylate/carbonate intermediates in the mechanisms. These processes may also benefit from the electrochemical methods to identify more active catalysts.^[21]^ For example, the industrial route to many bio‐derived plastics is heterocycle (lactone/cyclic carbonate) ring‐opening polymerization; coatings and surfactants are prepared using epoxide/anhydride ROCOP and switchable catalyses access recyclable plastics, thermoplastic elastomers, degradable adhesives and electrolytes.[^21^, ^22^] To accelerate uptake of these sustainable polymers, which all operate by mechanisms involving Lewis acidic but labile oxygenated catalyst intermediates, use of active site reduction potential may help to identify promising new catalysts and optimise known structures. A greater diversity of carbon dioxide utilization technologies will be needed to deliver a future ‘net‐zero’ chemical industry, yet very few are sufficiently understood for scaled operation at appropriate cost.^[2a,23**]**^

## Conclusion

A systematic series of heterodinuclear Co(III)/K(I) catalysts, differing by the backbone linker group, were compared for propene oxide/carbon dioxide ring opening copolymerization—an important carbon dioxide utilization process. A direct correlation between the active site (Co) reduction potential, and its electron density, was discovered which directly correlated with the catalytic activity and selectivity. The best catalysts are the most electron‐rich; they are both faster and more selective for poly(propene carbonate) formation. The ability to correlate catalyst performance data with an easy‐to‐measure parameter applied to the catalyst ground state is both very unusual and important both to understand the mechanism and design better catalysts in the future. The method could be broadly applicable to other types of catalyst and other reactions; it is particularly recommended to accelerate other sustainable polymerizations and carbon dioxide utilizations.

## Supporting Information

The authors have cited additional references within the Supporting Information.^[24]^ CCDC 2222739.^[25]^

## Conflict of interest

CKW is a director of Econic Technologies.

1

## Supporting information

As a service to our authors and readers, this journal provides supporting information supplied by the authors. Such materials are peer reviewed and may be re‐organized for online delivery, but are not copy‐edited or typeset. Technical support issues arising from supporting information (other than missing files) should be addressed to the authors.

Supporting Information

Supporting Information

## Data Availability

The data that support the findings of this study are available from the corresponding author upon reasonable request.
