# Supplementary material for: Correlating Metal Redox Potentials to Co(III)K(I) Catalyst Performances in Carbon Dioxide and Propene Oxide Ring Opening Copolymerization
Source: Angew Chem Int Ed Engl. 2023 Aug 7;62(37):e202308378. doi: 10.1002/anie.202308378 (PMC10952574; doi:10.1002/anie.202308378)
Supplement: Supplementary file 2 — Supporting Information [file ANIE-62-0-s002.pdf]

Angewandte Chemie
Eine Zeitschrift der Gesellschaft Deutscher Chemiker

Supporting Information

Correlating Metal Redox Potentials to Co(III)K(I) Catalyst Performances in Carbon Dioxide and Propene Oxide Ring Opening Copolymerization

W. Lindeboom, A. C. Deacy, A. Phanopoulos, A. Buchard, C. K. Williams**

Supporting Information

Table of Contents

Experimental Section 3 – 5
Table S1 Selected Atom Distances and Angles from Solid State Structure of $\mathbf{2_H}$. 6
Figure S1 Molecular structure of **1** and $\mathbf{2_H}$. 6
Figure S2 ^{1}H NMR of $\mathbf{2_H}$ ($CDCl_3$, 298K). 7
Figure S3 ^{13}C{^{1}H} NMR of $\mathbf{2_H}$ ($CDCl_3$, 298K). 7
Figure S4 2D COSY NMR of $\mathbf{2_H}$ ($CDCl_3$, 298K). 8
Figure S5 2D HSQC NMR of $\mathbf{2_H}$ ($CDCl_3$, 298K). 8
Figure S6 2D HMBC NMR of $\mathbf{2_H}$ ($CDCl_3$, 298K). 9
Figure S7 ^{1}H NMR of $\mathbf{2_{Me}}$ ($CDCl_3$, 298K). 9
Figure S8 ^{13}C{^{1}H} NMR of $\mathbf{2_{Me}}$ (CD_3OD, 298K). 10
Figure S9 2D COSY NMR of $\mathbf{2_{Me}}$ (CD_3OD, 298K). 10
Figure S10 2D HSQC NMR of $\mathbf{2_{Me}}$ (CD_3OD, 298K). 11
Figure S11 2D HMBC NMR of $\mathbf{2_{Me}}$ (CD_3OD, 298K). 11
Figure S12 ^{1}H NMR of $\mathbf{2_{OMe}}$ ($CDCl_3$, 298K). 12
Figure S13 ^{13}C{^{1}H} NMR of $\mathbf{2_{OMe}}$ ($CDCl_3$, 298K). 12
Figure S14 2D COSY NMR of $\mathbf{2_{OMe}}$ ($CDCl_3$, 298K). 13
Figure S15 2D HSQC NMR of $\mathbf{2_{OMe}}$ ($CDCl_3$, 298K). 13
Figure S16 2D HMBC NMR of $\mathbf{2_{OMe}}$ ($CDCl_3$, 298K). 14
Figure S17 ^{1}H NMR of $\mathbf{2_F}$ ($CDCl_3$, 298K). 14
Figure S18 ^{13}C{^{1}H} NMR of $\mathbf{2_F}$ ($CDCl_3$, 298K). 15
Figure S19 2D COSY NMR of $\mathbf{2_F}$ ($CDCl_3$, 298K). 15
Figure S20 2D HSQC NMR of $\mathbf{2_F}$ ($CDCl_3$, 298K). 16
Figure S21 2D HMBC NMR of $\mathbf{2_F}$ ($CDCl_3$, 298K). 16
Figure S22 ^{1}H NMR of $\mathbf{2_{Cl}}$ ($CDCl_3$, 298K). 17
Figure S23 ^{13}C{^{1}H} NMR of $\mathbf{2_{Cl}}$ ($CDCl_3$, 298K). 17
Figure S24 2D COSY NMR of $\mathbf{2_{Cl}}$ ($CDCl_3$, 298K). 18
Figure S25 2D HSQC NMR of $\mathbf{2_{Cl}}$ ($CDCl_3$, 298K). 18
Figure S26 2D HMBC NMR of $\mathbf{2_{Cl}}$ ($CDCl_3$, 298K). 19
Figure S27 IR spectra of Complexes $\mathbf{2_R}$. 20
Figure S28 CV curves of Complexes $\mathbf{2_R}$. 21
Table S2 Summary of Electrochemical Data for Complexes **1**-$\mathbf{2_R}$. 22
Figure S29 a) Absorbance versus time plot for PPC Formation for $\mathbf{2_{Me}}$. b) Logarithmic plot to calculate k_{obs} for $\mathbf{2_{Me}}$. 22
Figure S30 a) Absorbance versus time plot for PPC Formation for $\mathbf{2_{OMe}}$. b) Logarithmic plot to calculate k_{obs} for $\mathbf{2_{OMe}}$. 22
Figure S31 a) Absorbance versus time plot for PPC Formation for $\mathbf{2_F}$. b) Logarithmic plot to calculate k_{obs} for $\mathbf{2_F}$. 22
Figure S32 a) Absorbance versus time plot for PPC Formation for $\mathbf{2_{Cl}}$. b) Logarithmic plot to calculate k_{obs} for $\mathbf{2_{Cl}}$. 23
Figure S33 Conversion versus Selectivity plot of PO/CO_2 polymerisation $\mathbf{2_H}$ (0-20% conversion). 24
Figure S34 Exemplar NMR of polymerisation aliquot of PO/CO_2 polymerisation used to determine conversion and selectivity. 24
Table S3 ROCOP of CO_2/PO of Complexes **1**-$\mathbf{2_R}$. 25
Figure S35 GPC trace of PO/CO_2 polymerisation run from Table 1 of $\mathbf{2_{Me}}$. 25
Figure S36 GPC trace of PO/CO_2 polymerisation run from Table 1 of $\mathbf{2_{OMe}}$. 25
Figure S37 GPC trace of PO/CO_2 polymerisation run from Table 1 of $\mathbf{2_H}$. 26

Figure S38 GPC trace of PO/CO_2 polymerisation run from Table 1 of $\mathbf{2_F}$. 26
Figure S39 GPC trace of PO/CO_2 polymerisation run from Table 1 of $\mathbf{2_{Cl}}$. 26
Figure S40 a) E_{Red}(III/II) vs. k_p plot of complexes **1**-$\mathbf{2_R}$. b) E_{Red}(III/II) vs. PPC selectivity plot of complexes **1**-$\mathbf{2_R}$. 27
Figure S41 a) Imine Chemical Shift versus log $k_{p,\ 2R}$. b) Imine Chemical Shift versus PPC selectivity. 27
Figure S42 a) Activity Hammett plot of complexes $\mathbf{2_R}$. b) Selectivity Hammett plot of complexes $\mathbf{2_R}$. 27
Figure S43 a) Eyring analysis of backbiting of PPC using $\mathbf{2_{Cl}}$. b) reaction scheme of consumption of PPC to PC. 28
General X-ray crystallography experimental 28
Density Functional Theory Calculations 28
Figure S44 Previously calculated lowest energy pathway for the propagation sequence of the alternating copolymerisation of *R*-PO and carbon dioxide. Key intermediates and transition states to be analysed have been highlighted. 30
Table S4 Computed Gibbs free energies of key intermediates and transition states identified for the propagation sequence of the alternating copolymerisation of *R*-PO and carbon dioxide using $\mathbf{2_H}$. 30
Table S5 Computed Gibbs free energies of key intermediates and transition states identified for the propagation sequence of the alternating copolymerisation of *R*-PO and carbon dioxide using $\mathbf{2_F}$. 31
Table S6 Computed Gibbs free energies of key intermediates and transition states identified for the propagation sequence of the alternating copolymerisation of *R*-PO and carbon dioxide using $\mathbf{2_{Cl}}$. 31
Table S7 Computed Gibbs free energies of key intermediates and transition states identified for the propagation sequence of the alternating copolymerisation of *R*-PO and carbon dioxide using $\mathbf{2_{OMe}}$. 32
Table S8 Computed Gibbs free energies of key intermediates and transition states identified for the propagation sequence of the alternating copolymerisation of *R*-PO and carbon dioxide using $\mathbf{2_{Me}}$. 32
Table S9 Comparison of calculated ranges for various parameters to differentiate catalysts $\mathbf{2_R}$. 33
Figure S45 Comparison of Co–O bond length in ***0_phenR*** against propagation rate coefficient. 33
Figure S46 Comparison of Co–O WBIs in ***0_phenR*** against propagation rate coefficient. 34
Figure S47 Comparison of PO ring-opening transition state barrier ($\mathbf{TS_{I\text{-}II}}$) against propagation rate coefficient. 34
Figure S48 Comparison of CO_2 insertion transition state barrier ($\mathbf{TS_{IV\text{-}V}}$) against propagation rate coefficient. 35
Figure S49 Comparison of free energy for **II** against propagation rate coefficient. 35
Figure S50 Comparison of free energy for **VI** against propagation rate coefficient. 36
Figure S51 Comparison of free energy difference between **II** and **VI** against propagation rate coefficient. 36
Figure S52 Fragmentation used during Activation Strain Analysis. 37
Figure S53 Activation Strain Analysis during approach of $\mathbf{TS_{I\text{-}II}}$ for $\mathbf{2_H}$ plotted as a function of the breaking bond. 38
Figure S54 Activation Strain Analysis during approach of $\mathbf{TS_{I\text{-}II}}$ for $\mathbf{2_H}$ plotted as a function of the forming bond. 38
Figure S55 Activation Strain Analysis during approach of $\mathbf{TS_{I\text{-}II}}$ for $\mathbf{2_{Cl}}$ plotted as a function of the breaking bond. 39
Figure S56 Activation Strain Analysis during approach of $\mathbf{TS_{I\text{-}II}}$ for $\mathbf{2_{Cl}}$ plotted as a function of the forming bond. 39
Computational coordinates 40
References 62

Experimental Section

General Procedures

All experimental manipulations were performed using a dual-manifold nitrogen-vacuum Schlenk line or in a nitrogen filled glovebox. All solvents and reagents were obtained from commercial sources and used as received, unless stated otherwise. Acetonitrile, pentane and toluene were obtained from an SPS system, degassed by several freeze-pump-thaw cycles, further dried with 3 Å molecular sieves and stored under N_2. The epoxide monomers were dried, overnight over calcium hydride, and purified by fractional distillation, followed by degassing with N_2 and stored under N_2. CO_2 gas (BOC, CP grade, 99.995 %) was passed through two carbon dioxide purifiers (VICI Metronics carbon dioxide purifier) in series, at 50 bar pressure, before use at lower pressures in the copolymerizations. The pro-ligand (LH_2) was purchased from Enamine.

^{1}H, COSY, HSQC, HMBC, ^{19}F NMR spectra were measured using a Bruker AVIII HD 400 NMR spectrometer. $^{13}C\{^{1}H\}$ NMR spectra were determined using a Bruker AV III 500, equipped with a cryoprobe. All spectra were recorded at 298 K, unless stated otherwise. FT-IR spectra were obtained on a Shimadzu IR Spirit spectrometer, fitted with a KBr window and DLATGS detector, with temperature control. Gel permeation chromatography (GPC) was conducted using a Shimadzu LC-20AD instrument, at 40 °C, with two mixed bed PSS SDV linear S columns in series, and with THF as eluent at a flow rate of 1 mL/min. Molar mass values were calibrated using narrow molar mass polystyrene standards.

General Procedure for PO/CO_2 ROCOP

A solution of catalyst (13 mg, 0.02 mmol), 1,2- cyclohexane diol (46 mg, 0.4 mmol) and mesitylene (30 µL. 0.2 mmol, internal standard) in PO (6 mL, 85.7 mmol) was injected into a 100 mL Parr reactor, under a stream of dry CO_2. The reactor was also fitted with a DiComp sentinel probe, attached to an ATR-IR spectrometer, which allowed for continual monitoring of PPC formation. The reactor was then pressurized with CO_2 to the target reaction pressure and allowed to reach the required temperature. Upon reaction completion, the reactor vessel was cooled to room temperature and depressurized slowly, with cooling. The catalyst was quenched by the slow addition of benzoic acid (1 M solution in $CHCl_3$). A sample of the crude reaction mixture was removed for NMR spectroscopy and GPC analysis. The polymer was precipitated by dropwise addition of the crude solution into methanol, the solution was decanted and polymer dried *in vacuo*.

General Procedure for PC Formation (Backbiting Reactions)

A 100 mL Parr reactor was charged with catalyst (11 mg, 0.018 mmol) and a solution of poly(propylene carbonate) (0.18 g, 1.8 mmol) in propene oxide (6 mL, 86 mmol). The reactor was fitted with a DiComp sentinel probe, attached to an ATR-IR spectrometer, which allowed for continual monitoring of PPC consumption and PC formation. The reactor was then heated to the desired temperature. Upon reaction completion, the reactor vessel was cooled to room temperature and a sample of the crude mixture removed for NMR spectroscopy.

Synthesis of 2_H

A Schlenk tube was charged with LH_2 (300 mg, 0.77 mmol), $Co(OAc)_2$ (136 mg, 0.77 mmol), KOAc (74 mg, 0.77 mmol) and dry MeCN (40 mL), under a nitrogen atmosphere. The solution was stirred for 2 hours, before adding 1,2-phenylenediamine (83 mg, 0.77 mmol) and left to stir overnight. Then, the reaction was exposed to air and acetic acid (44 µL, 0.77 mmol). The solution was stirred for a further 48 hours and then filtered and the solvent was removed under vacuum. The solid was triturated with toluene (3 x 50 mL) and pentane (3 x 50 mL) and the solution evaporated to dryness to give the pure product (219 mg, 68% yield).

^{1}H NMR (400 MHz, $CDCl_3$, 298K). δ(ppm): 8.19 (2H, s, –**H**C=N–), 8.01 (2H, dd, HC–**H**C=C–N–, $^{3}J_{H\text{-}H}$ = 6.1, 3.4 Hz), 7.37 (2H, dd, **H**C–HC=C–N–, $^{3}J_{H\text{-}H}$ = 6.2, 3.4 Hz), 7.01 (2H, d, Ar–$\mathbf{H}_{meta}$, $^{3}J_{H\text{-}H}$ = 8.1 Hz), 6.73 (2H, d, Ar–$\mathbf{H}_{meta}$, $^{3}J_{H\text{-}H}$ = 7.5 Hz), 6.47 (2H, t, Ar–$\mathbf{H}_{para}$, $^{3}J_{H\text{-}H}$ = 7.5 Hz), 4.24-3.78 (12H, m, –O–C$\mathbf{H}_2$–), 1.36l (6H, s, $\mathbf{H}_3$C–C(O)O). ^{13}C NMR (125 MHz, $CDCl_3$, 298K). δ(ppm): 179.50 (–**C**(O)O), 158.88 (–H**C**=N–), 157.20 (Ar–$\mathbf{C}_{ipso}$–O^-, Ar–$\mathbf{C}_{ortho}$–O/CH), 151.91 (Ar–$\mathbf{C}_{ipso}$–O^-, Ar–$\mathbf{C}_{ortho}$–O/CH), 147.05 (Ar–**C**–N), 127.32 (H**C**–HC=C–N–, Ar–$\mathbf{C}_{Meta}$), 118.55 (Ar–$\mathbf{C}_{ipso}$–O^-, Ar–$\mathbf{C}_{ortho}$–O/CH), 115.62 (HC–H**C**=C–N–), 113.16 + 113.04 (Ar–$\mathbf{C}_{para}$, Ar–$\mathbf{C}_{meta}$), 70.22 (–O–CH_2–), 69.53 (–O–$\mathbf{C}H_2$–), 66.21 (–O–$\mathbf{C}H_2$–), 24.88 (H_3**C**–C(O)O). Calculated; C, 53.3; H, 4.5; N, 4.1 %. Found; C, 51.3; H, 4.4; N, 4.0 %.

Synthesis of 2_{Cl}

A Schlenk tube was charged with LH_2 (300 mg, 0.77 mmol), $Co(OAc)_2$ (136 mg, 0.77 mmol), KOAc (74 mg, 0.77 mmol) and dry MeCN (40 mL), under a nitrogen atmosphere. The solution was stirred for 2 hours, before adding 4,5-dichloro-1,2-phenylenediamine (136 mg, 0.77 mmol) and left to stir overnight. The reaction was exposed to air and acetic acid (44 µL, 0.77 mmol). The solution was stirred for a further 48 hours and then filtered and solvent removed under vacuum. The resulting solid was dissolved in a minimum amount of methanol and precipitated with diethyl ether. The solid was triturated with toluene (3 x 50 mL) and pentane (3 x 50 mL). The solution was filtered and solvent removed under vacuum to collect the product (258 mg, 45% yield).

^{1}H NMR (400 MHz, $CDCl_3$, 298K). δ(ppm): 8.04 (4H, s, –**H**C=N–, ClC–**H**C=C–N–), 6.98 (2H, d, Ar–**H**$_{meta}$, $^3J_{H-H}$ = 8.1 Hz), 6.72 (2H, d, Ar–**H**$_{meta}$, $^3J_{H-H}$ = 7.5 Hz), 6.46 (2H, t, Ar–**H**$_{para}$, $^3J_{H-H}$ = 7.8 Hz), 4.24-3.82 (12H, m, –O–C**H**$_2$–), 1.38 (6H, s, **H**$_3$C–C(O)O). ^{13}C NMR (125 MHz, $CDCl_3$, 298K). δ(ppm): 179.38 (–**C**(O)O), 158.90 (–H**C**=N–), 157.21 (Ar–**C**$_{ipso}$–O$^-$, Ar–**C**$_{ortho}$–O/CH), 151.64 (Ar–**C**$_{ipso}$–O$^-$, Ar–**C**$_{ortho}$–O/CH), 146.15 (Ar–**C**–Cl, Ar–**C**–N), 130.67 (Ar–**C**–Cl, Ar–**C**–N), 127.04 (Ar–**C**$_{meta}$), 117.78 (Ar–ClC=**C**H–), 116.36 (Ar–**C**$_{ipso}$–O$^-$, Ar–**C**$_{ortho}$–O/CH), 113.36 + 113.25 (Ar–**C**$_{para}$, Ar–**C**$_{meta}$), 69.90 (–O–**C**H$_2$–), 69.03 (–O–**C**H$_2$–), 66.10 (–O–**C**H$_2$–), 24.46 (H$_3$**C**–C(O)O). Calculated; C, 48.3; H, 3.8; N, 3.8 %. Found; C, 48.0; H, 4.1; N, 3.7 %.

Synthesis of 2_F

A Schlenk tube was charged with LH_2 (300 mg, 0.77 mmol), $Co(OAc)_2$ (136 mg, 0.77 mmol), KOAc (74 mg, 0.77 mmol) and dry MeCN (40 mL), under a nitrogen atmosphere. The solution was stirred for 2 hours before adding 4,5-difluoro-1,2-phenylenediamine (111 mg, 0.77 mmol) and left to stir overnight. The reaction was exposed to air and acetic acid (44 µL, 0.77 mmol). The solution was stirred for a further 48 hours and then filtered and solvent removed under vacuum. The solid was dissolved in a minimum methanol and precipitated with diethyl ether. The solid was triturated with toluene (3 x 50 mL) and pentane (3 x 50 mL). The solution was filtered and solvent removed under vacuum to collect the product (50 mg, 9% yield).

^{1}H NMR (400 MHz, $CDCl_3$, 298K). δ(ppm): 7.95 (2H, s, –**H**C=N–), 7.79 (2H, t, FC–**H**C=C–N–, $^3J_{H-F}$ = 9.1 Hz), 6.98 (2H, d, Ar–**H**$_{meta}$, $^3J_{H-H}$ = 7.8 Hz), 6.74 (2H, d, Ar–**H**$_{meta}$, $^3J_{H-H}$ = 7.5 Hz), 6.48 (2H, t, Ar–**H**$_{para}$, $^3J_{H-H}$ = 7.8 Hz), 4.20-3.82 (12H, m, –O–C**H**$_2$–), 1.38 (6H, s, **H**$_3$C–C(O)O). ^{13}C NMR (125 MHz, $CDCl_3$, 298K). δ(ppm): 179.45 (–**C**(O)O), 158.77 (–H**C**=N–), 157.18 (Ar–**C**$_{ipso}$–O$^-$, Ar–**C**$_{ortho}$–O/CH), 151.88 (Ar–**C**$_{ipso}$–O$^-$, Ar–**C**$_{ortho}$–O/CH), 143.16 (Ar–**C**–F, Ar–**C**–N), 127.27 (Ar–**C**$_{meta}$), 118.13 (Ar–**C**$_{ipso}$–O$^-$, Ar–**C**$_{ortho}$–O/CH), 113.51 + 113.45 (Ar–**C**$_{para}$, Ar–**C**$_{meta}$), 103.86 (Ar–FC–**C**H–), 70.14 (–O–**C**H$_2$–), 69.38 (–O–**C**H$_2$–), 66.33 (–O–**C**H$_2$–), 24.85 (H$_3$**C**–C(O)O). Calculated; C, 50.6; H, 4.0; N, 3.9 %. Found; C, 50.2; H, 3.9; N, 4.2 %.

Synthesis of 2_{Me}

A Schlenk tube was charged with LH_2 (300 mg, 0.77 mmol), $Co(OAc)_2$ (136 mg, 0.77 mmol), KOAc (74 mg, 0.77 mmol) and dry MeCN (40 mL), under a nitrogen atmosphere. The solution was stirred for 2 hours before adding 4,5-dimethyl-1,2-phenylenediamine (111 mg, 0.77 mmol) and left to stir for two days. Next, the reaction mixture was exposed to air and acetic acid (44 µL, 0.77 mmol) was added and the solution was stirred for a further 48 hours. The solution was then filtered and the precipitate was dried under vacuum (280 mg, 52% yield).

^{1}H NMR (400 MHz, $CDCl_3$, 298K). δ(ppm): 8.14 (2H, s, –**H**C=N–), 7.78 (2H, s, MeC–**H**C=C–N–), 7.01 (2H, d, Ar–**H**$_{meta}$, $^3J_{H-H}$ = 8.0 Hz), 6.72 (2H, d, Ar–**H**$_{meta}$, $^3J_{H-H}$ = 7.5 Hz), 6.46 (2H, t, Ar–**H**$_{para}$, $^3J_{H-H}$ = 7.8 Hz), 4.20-3.80 (12H, m, –O–C**H**$_2$–), 2.41 (6H, s, **H**$_3$C–C(O)O) 1.39 (6H, s, **H**$_3$C–C=HC). ^{13}C NMR (125 MHz, CD_3OD, 298K). δ(ppm): 160.87 (–H**C**=N–), 158.64 (Ar–**C**$_{ortho}$–O/CH), 153.03 (Ar–**C**$_{para}$), 144.43 (Ar–**C**–N), 139.39 (Ar–**C**–Me), 128.58 (Ar–**C**$_{meta}$), 119.75 (Ar–**C**$_{ipso}$–O$^-$), 118.26 (Ar–**C**H–C–N), 115.92 + 115.69 (Ar–**C**$_{para}$, Ar–**C**$_{meta}$), 71.28 (–O–**C**H$_2$–), 70.13 (–O–**C**H$_2$–), 68.33 (–O–**C**H$_2$–), 49.45 (H$_3$**C**–C(O)O), 20.04 (Ar-**C**H$_3$). Calculated; C, 54.5; H, 4.9; N, 4.0 %. Found; C, 52.3; H, 5.0; N, 4.0 %.

Synthesis of 2_{OMe}

A round bottomed flask was charged with LH_2 (300 mg, 0.77 mmol), $Ba(ClO_4)_2$ (258 mg, 0.77 mmol) and methanol (300 mL) and left to stir for 1 h. 1,2-Diamino-4,5-dimethoxybenzene (129 mg, 0.77 mmol) was added to the reaction mixture and it was left to stir overnight. The solution was concentrated, under reduced pressure, and excess chloroform (250 mL) was added. Deionized water was added together with guanidine sulfate (831 mg, 7.7 mmol, 10 equiv.), the biphasic mixture was stirred overnight. The layers were separated and the aqueous layer was extracted with chloroform (3 x 50 mL). The combined organic layers were dried (magnesium sulfate) and the solvent was removed under a reduced pressure to yield the

intermediate macrocycle as an orange solid (250 mg, 60%). Next, a Schlenk tube was charged with the macrocycle (200 mg, 0.38 mmol), $Co(OAc)_2$ (68 mg, 0.38 mmol), KOAc (38 mg, 0.38 mmol) and dry MeCN (40 mL), under a nitrogen atmosphere. The solution was stirred overnight after which the reaction was exposed to air and acetic acid (22 µL, 0.38 mmol). The solution was stirred for a further 48 hours and then filtered and solvent removed under vacuum. The resulting solid was triturated toluene (3 x 50 mL) and pentane (3 x 50 mL). The combined solutions were filtered and the solvent was removed under vacuum to yield the product as a solid (75 mg, 27% yield).

^{1}H NMR (400 MHz, $CDCl_3$, 298K). δ(ppm): 8.15 (2H, s, –**H**C=N–), 7.79 (2H, s, MeOC–**H**C=C–N–), 7.01 (2H, s, Ar–$\mathbf{H}_{meta}$), 6.72 (2H, s, Ar–$\mathbf{H}_{meta}$), 6.47 (2H, s, Ar–$\mathbf{H}_{para}$), 4.20-3.80 (12H, m, –O–C$\mathbf{H}_2$–), 2.40 (6H, s, H_3C-O-Ar), 1.39 (6H, s, $\mathbf{H}_3$C–C(O)O). ^{13}C NMR (125 MHz, $CDCl_3$, 298K). δ(ppm): 179.47 (–**C**(O)O), 156.42 (–H**C**=N–), 151.91 (Ar–$\mathbf{C}_{ortho}$–O/CH), 145.13 (Ar–$\mathbf{C}_{ortho}$–O/CH), 136.26 (Ar–**C**–OMe, Ar–**C**–N), 127.24 (Ar–$\mathbf{C}_{meta}$), 118.67 (Ar–**C**–OMe, Ar–**C**–N), 116.38 (Ar–MeOC–**C**H–), 113.34 (Ar–$\mathbf{C}_{ipso}$–O^-), 112.94 + 112.83 (Ar–$\mathbf{C}_{para}$, Ar–$\mathbf{C}_{meta}$), 70.17 (–O–**C**H_2–), 69.50 (–O–**C**H_2–), 66.12 (–O–**C**H_2–), 24.95 (H_3**C**–C(O)O), 22.86 (–O–**C**H_3). Calculated; C, 52.2; H, 4.7; N, 3.8 %. Found; C, 51.2; H, 4.6; N, 4.1 %.

Table S1 Selected Bond Lengths and Angles from the Solid State Structure of **2$_H$**.

Atoms	Distance (Å)	Atoms	Angles (°)
K$_1$ - Co$_1$	3.5904 (17)	N$_1$ - Co$_1$ - O$_4$	88.27 (12)
Co$_1$ - N$_1$	1.907 (3)	N$_1$ - Co$_1$ - O$_1$	93.70 (13)
Co$_1$ - O$_1$	1.892 (3)	O$_{1'}$ - Co$_1$ - O$_1$	87.86 (17)
Co$_1$ - O$_4$	1.939 (3)	O$_4$ - Co$_1$ - O$_1$	94.28 (12)
k$_1$ - O$_1$	2.587 (3)	N$_1$ - Co$_1$ - N$_{1'}$	84.75 (19)
K$_1$ - O$_2$	2.713 (3)		
K$_1$ - O$_3$	2.819 (3)		
K$_1$ - O$_5$	2.875 (4)		
C$_1$ - O$_4$	1.264 (5)		

1

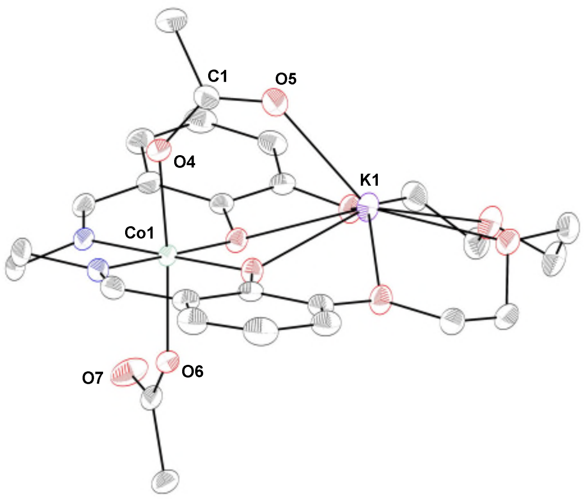

2$_H$

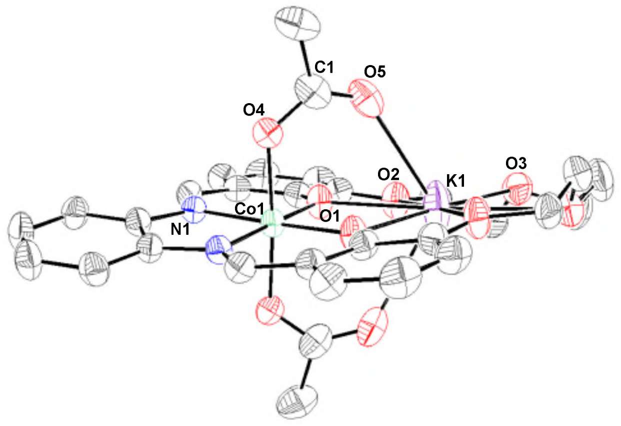

Atoms	Distance (Å)
C1-O4	1.280(2)
C1-O5	1.227(3)
Co1-K1	3.698(5)
C1-O4	1.291(2)
C1-O5	1.225(3)

Figure S1 Molecular structures, determined by single crystal X-ray diffraction methods, of **1** and **2$_H$**.

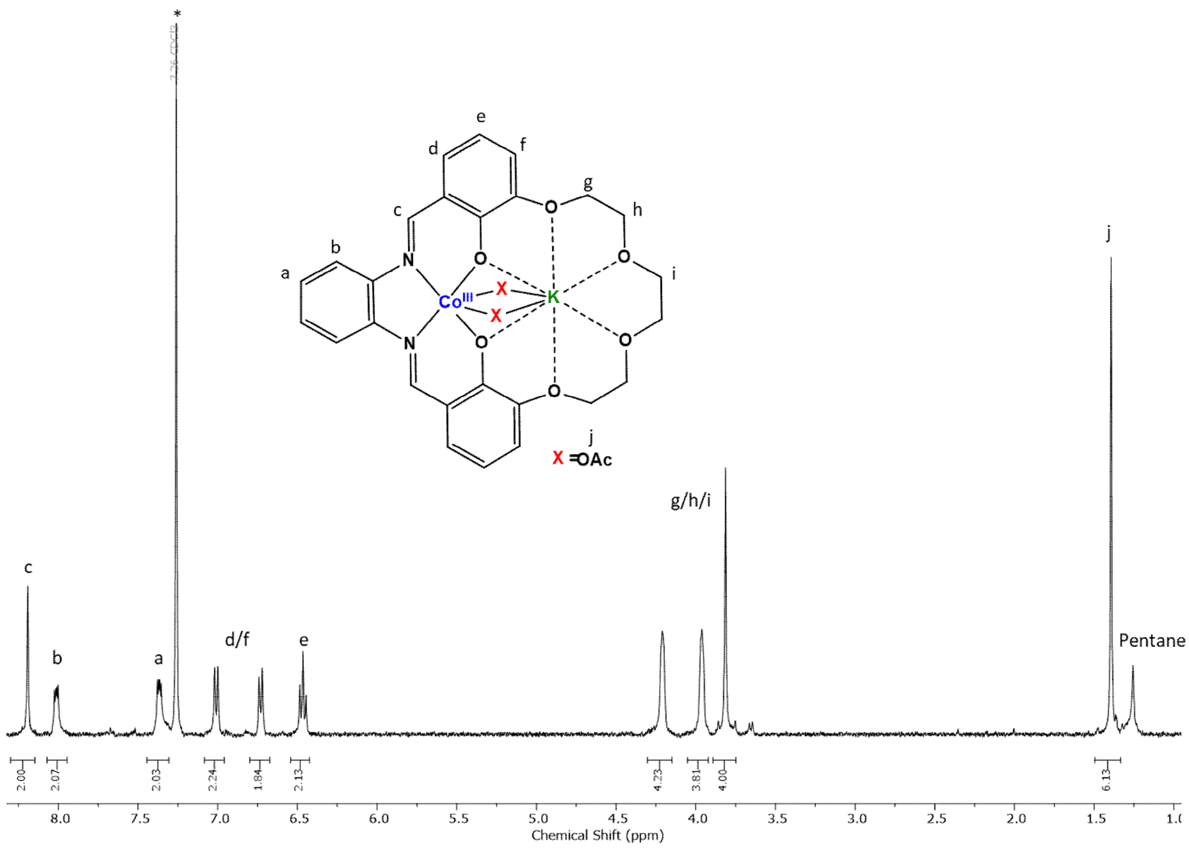

Figure S2 ^{1}H NMR Spectrum of $\mathbf{2_H}$ ($CDCl_3$, 298K).

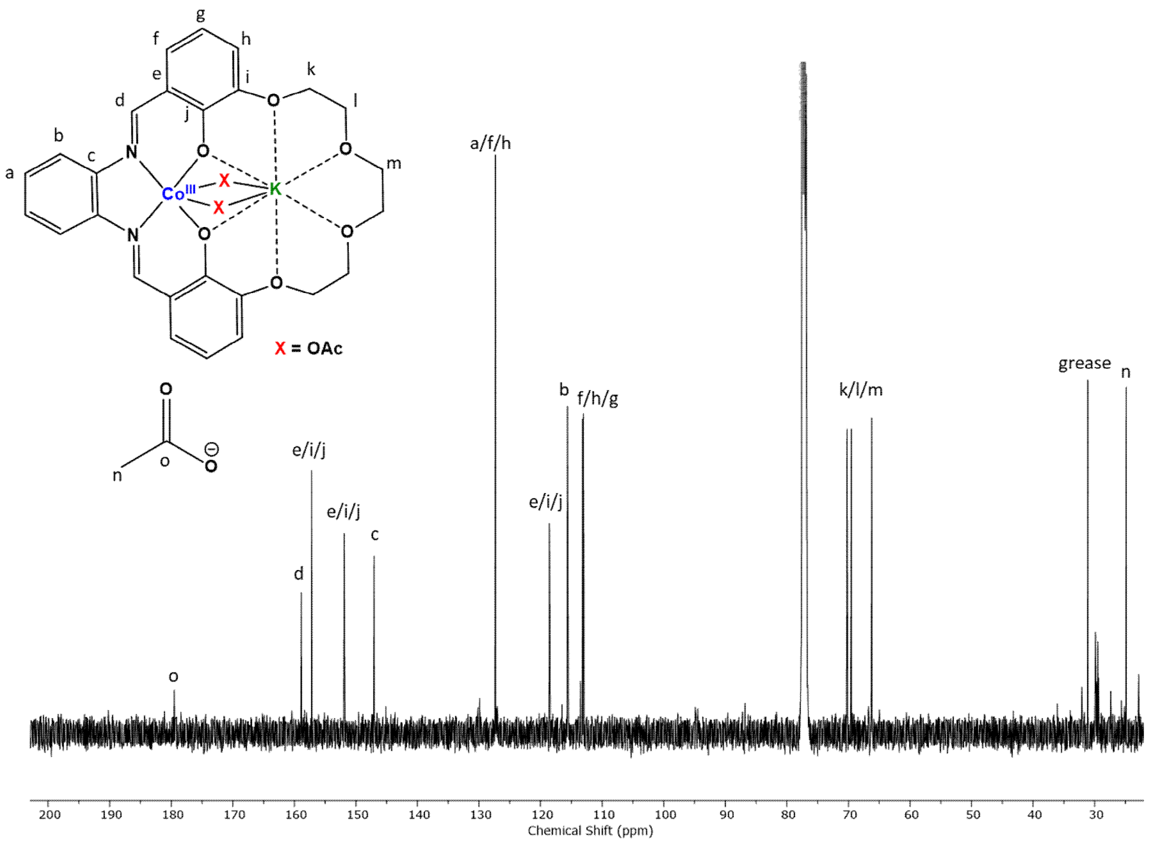

Figure S3 $^{13}C\{^{1}H\}$ NMR Spectrum of $\mathbf{2_H}$ ($CDCl_3$, 298K).

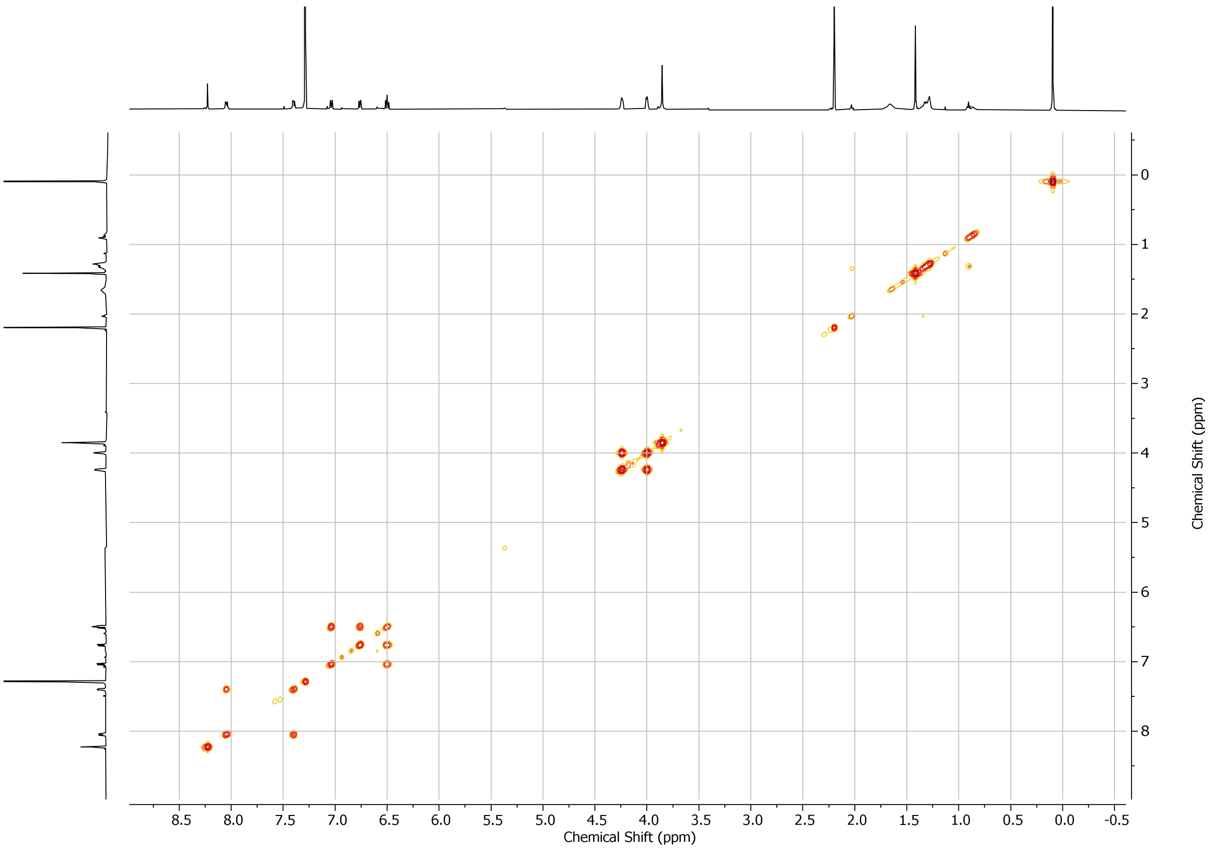

Figure S4 2D COSY NMR Spectrum of $\mathbf{2_H}$ ($CDCl_3$, 298K).

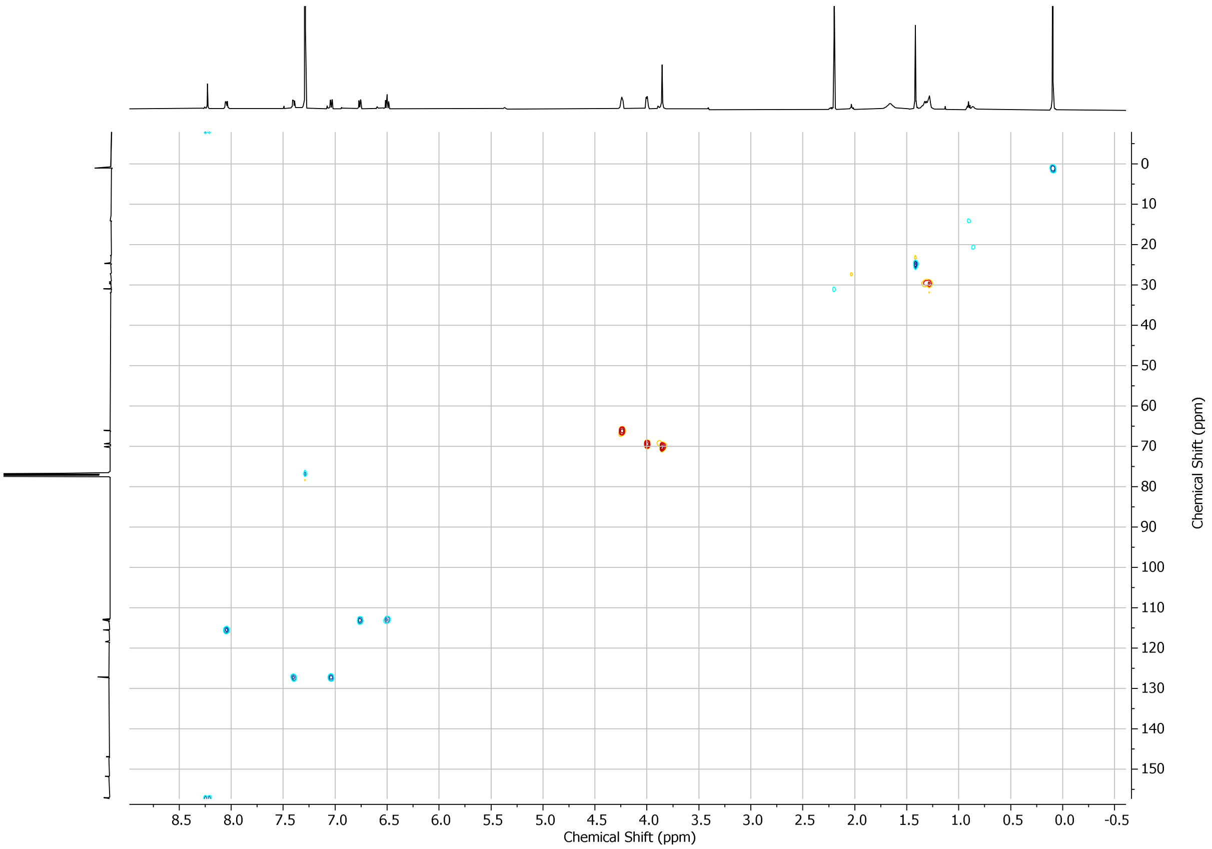

Figure S5 2D HSQC NMR Spectrum of $\mathbf{2_H}$ ($CDCl_3$, 298K).

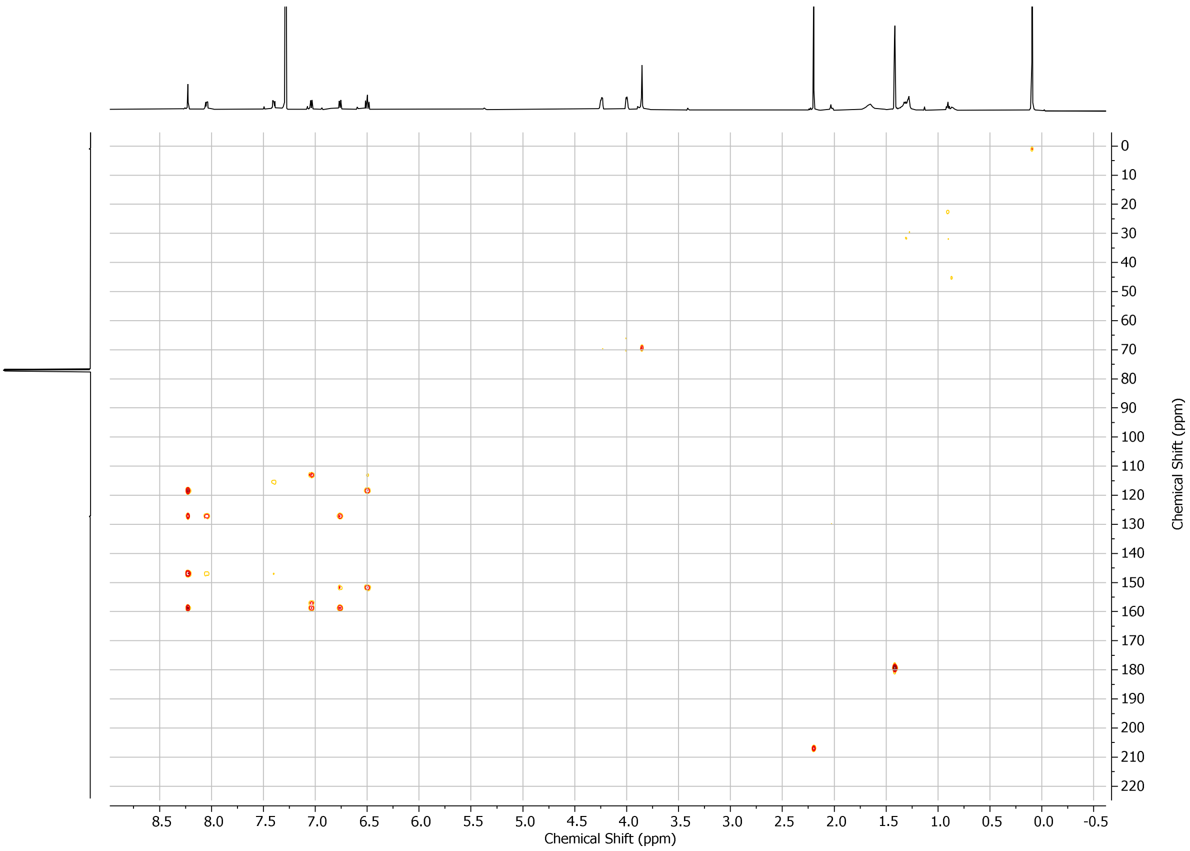

Figure S6 2D HMBC NMR Spectrum of **2$_{H}$** ($CDCl_3$, 298K).

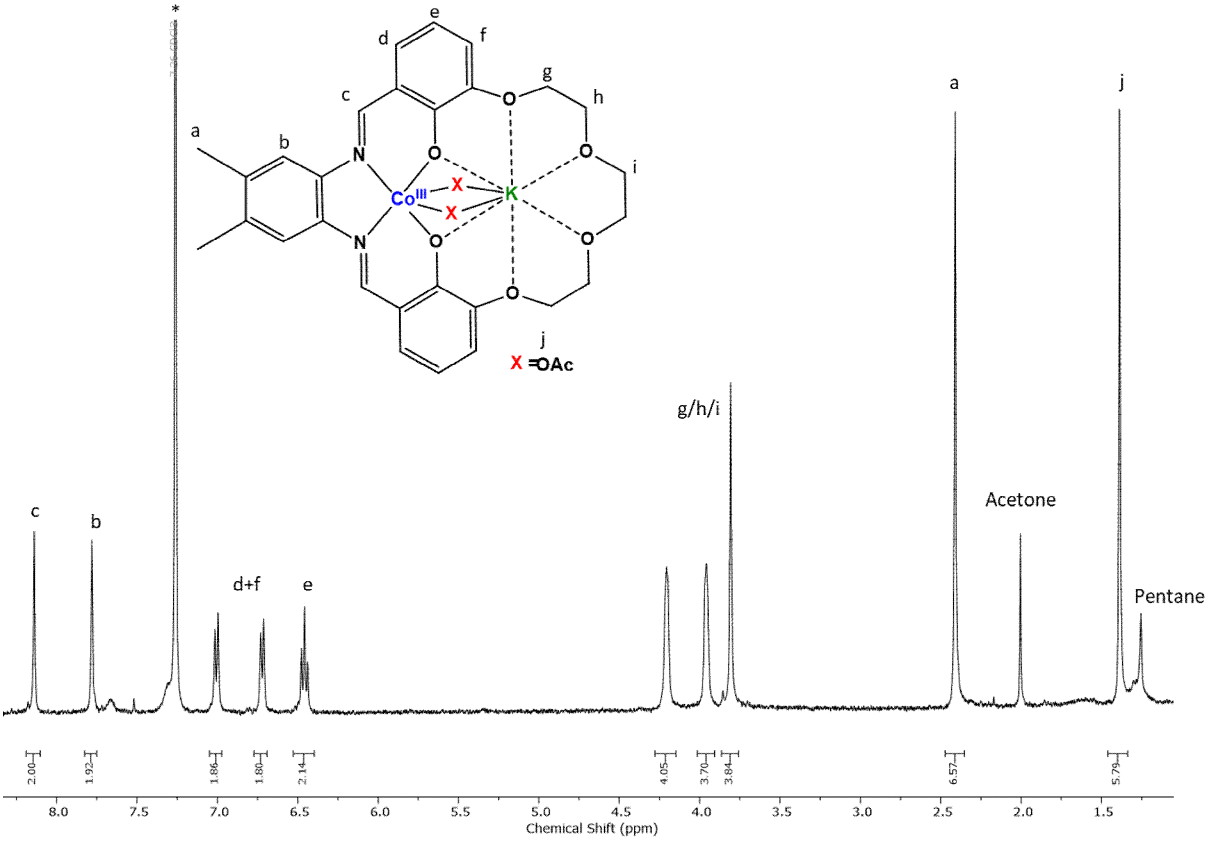

Figure S7 ^{1}H NMR Spectrum of **2$_{Me}$** ($CDCl_3$, 298K).

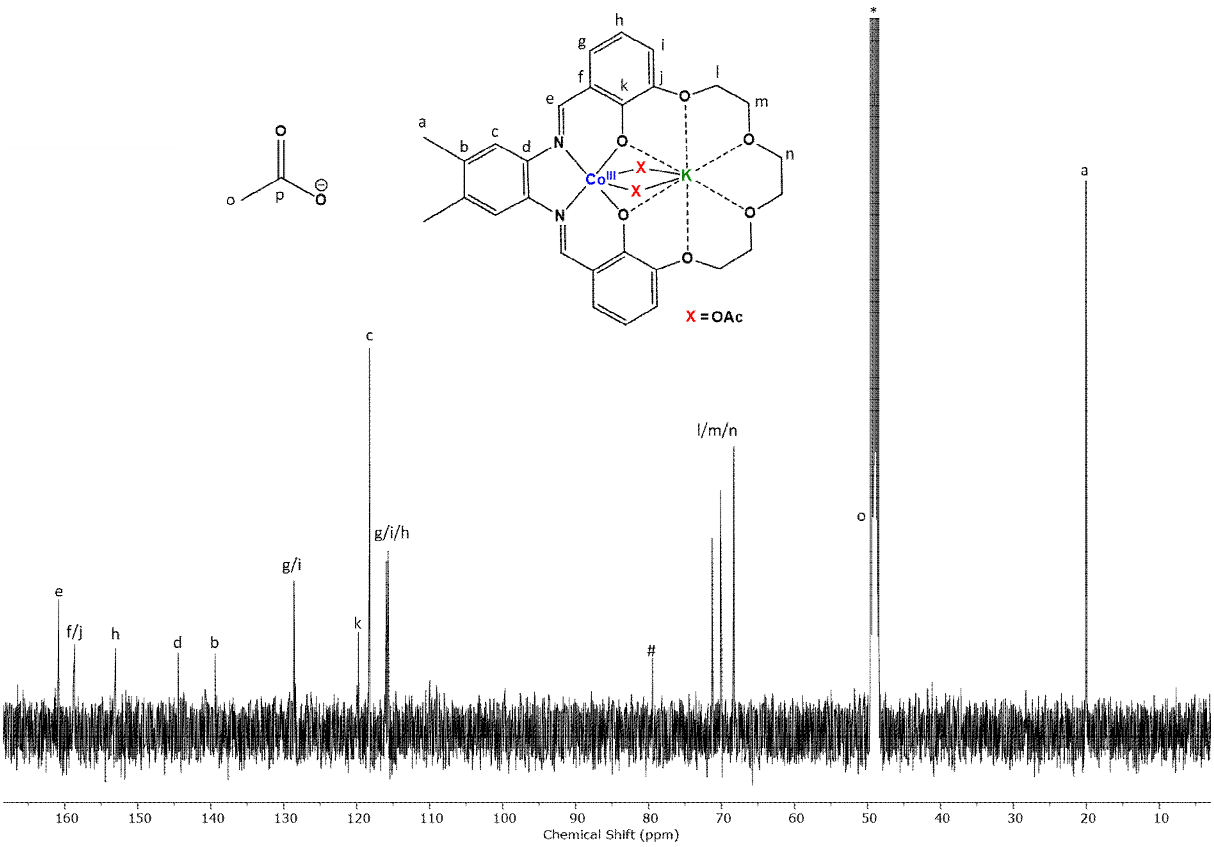

Figure S8 $^{13}C\{^{1}H\}$ NMR Spectrum of $\mathbf{2_{Me}}$ (CD_3OD, 298K).

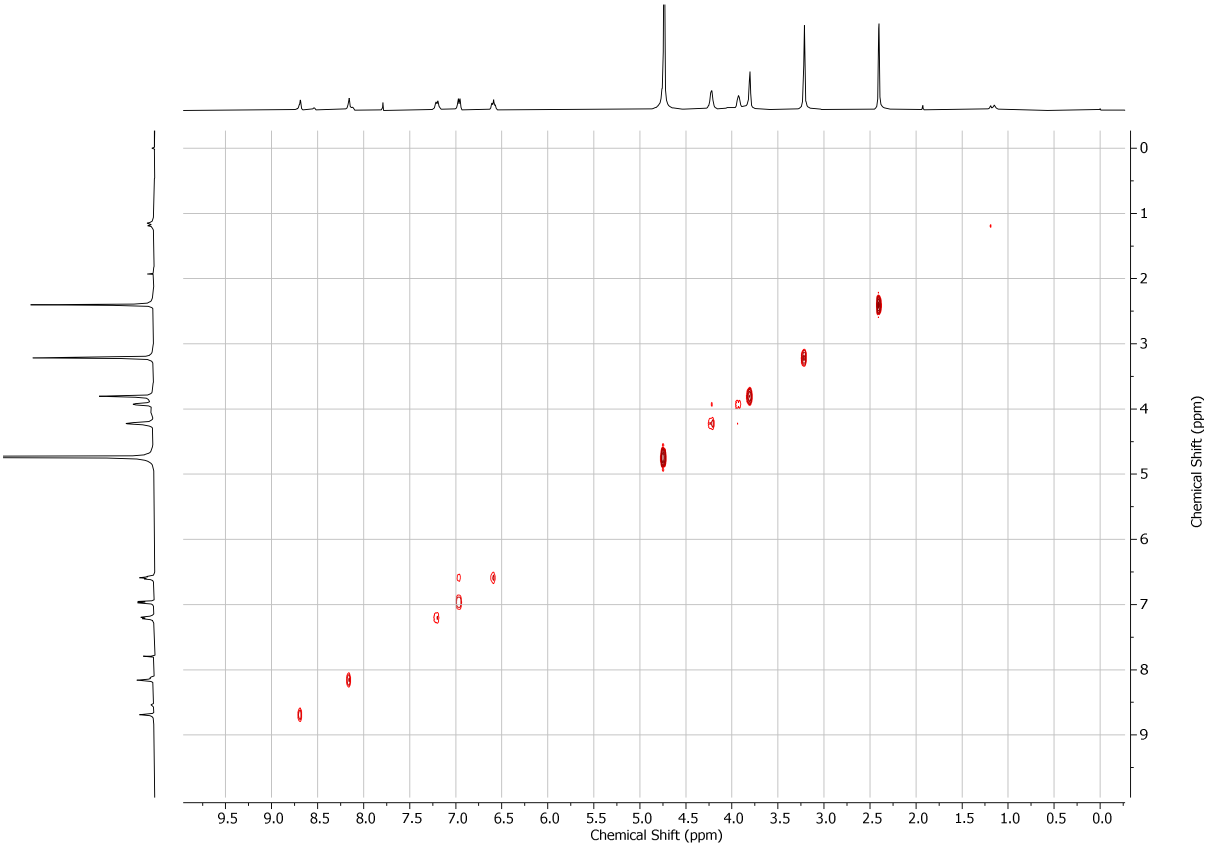

Figure S9 2D COSY NMR Spectrum of $\mathbf{2_{Me}}$ (CD_3OD, 298K).

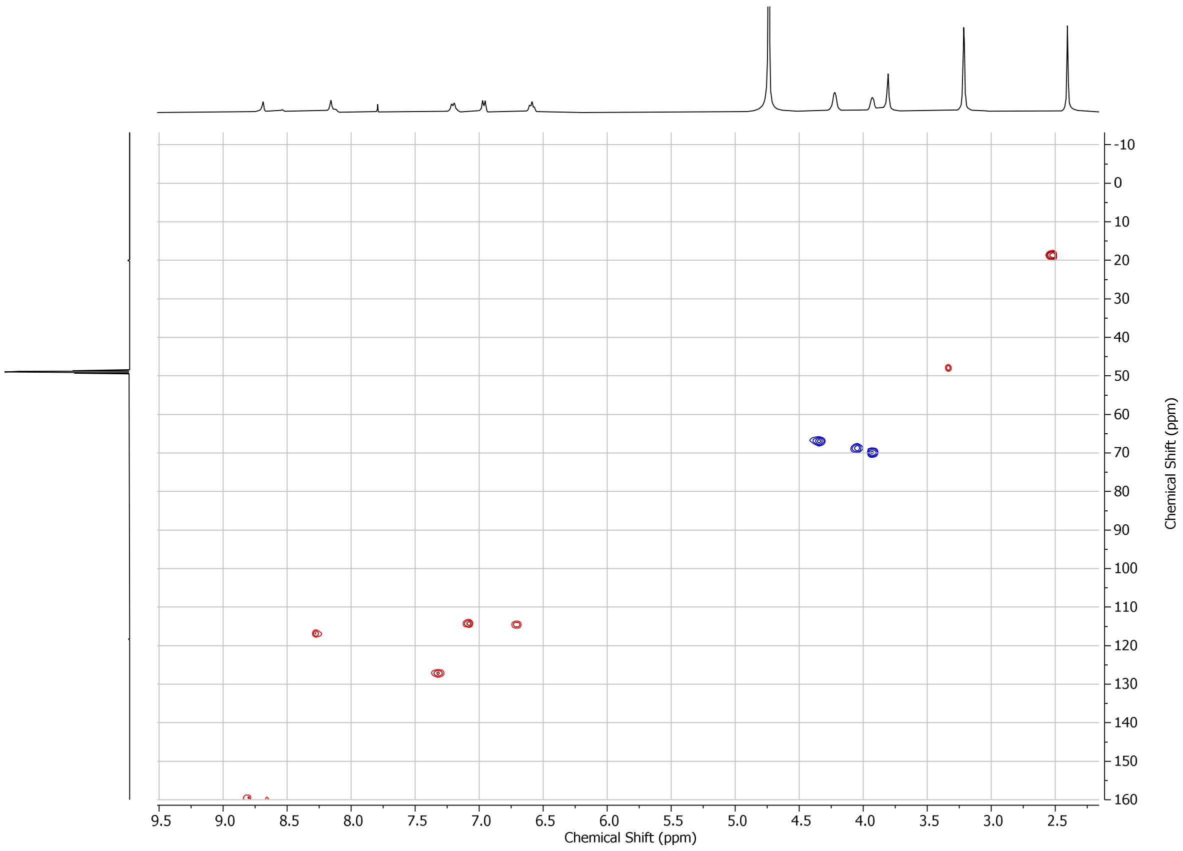

Figure S10 2D HSQC NMR Spectrum of $\mathbf{2}_{\mathbf{Me}}$ (CD_3OD, 298K).

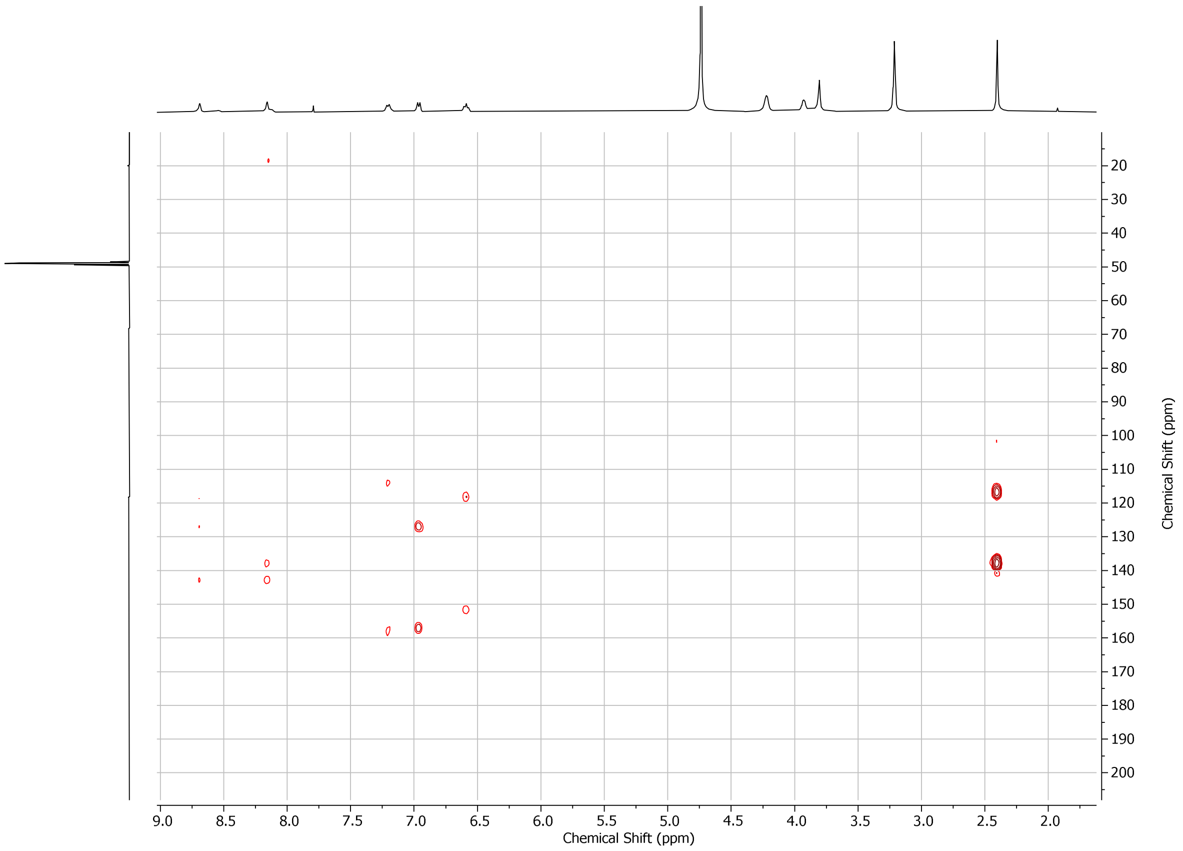

Figure S11 2D HMBC NMR Spectrum of $\mathbf{2}_{\mathbf{Me}}$ (CD_3OD, 298K).

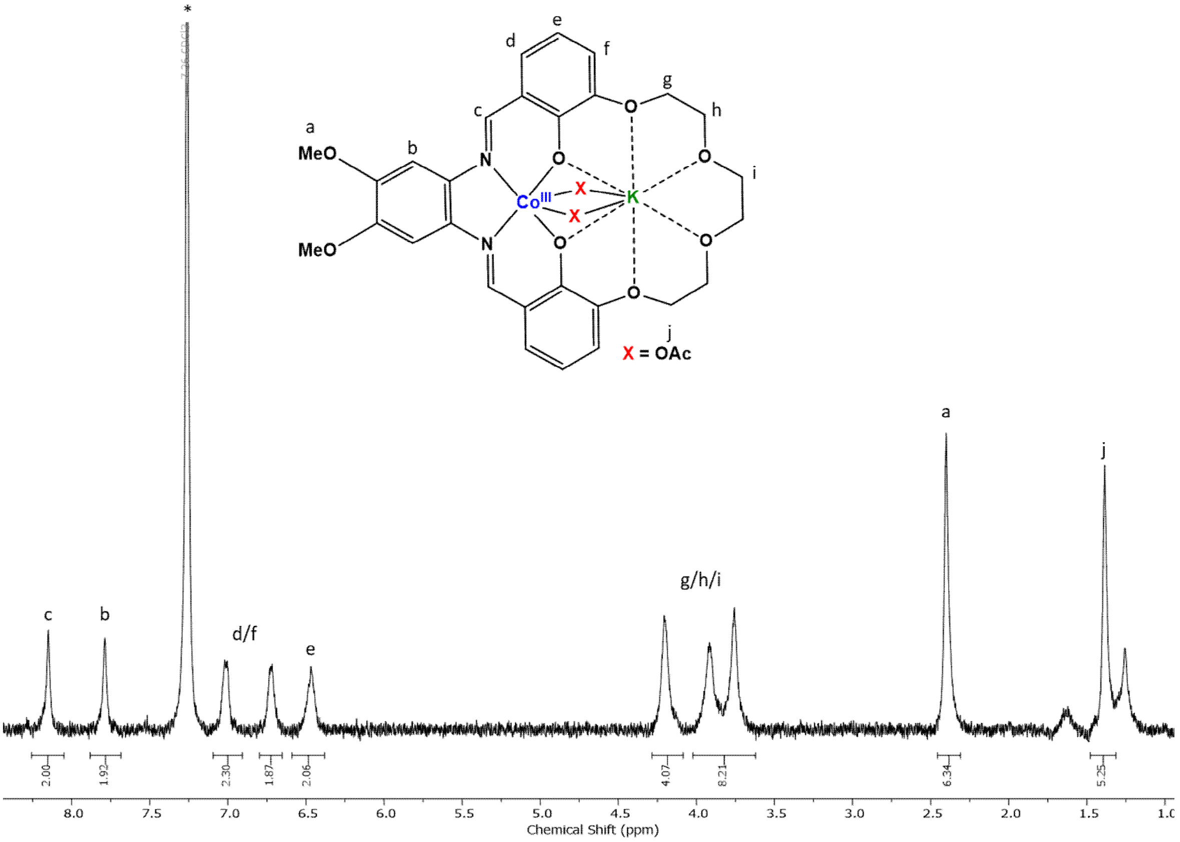

Figure S12 ^{1}H NMR Spectrum of $\mathbf{2_{OMe}}$ ($CDCl_3$, 298K).

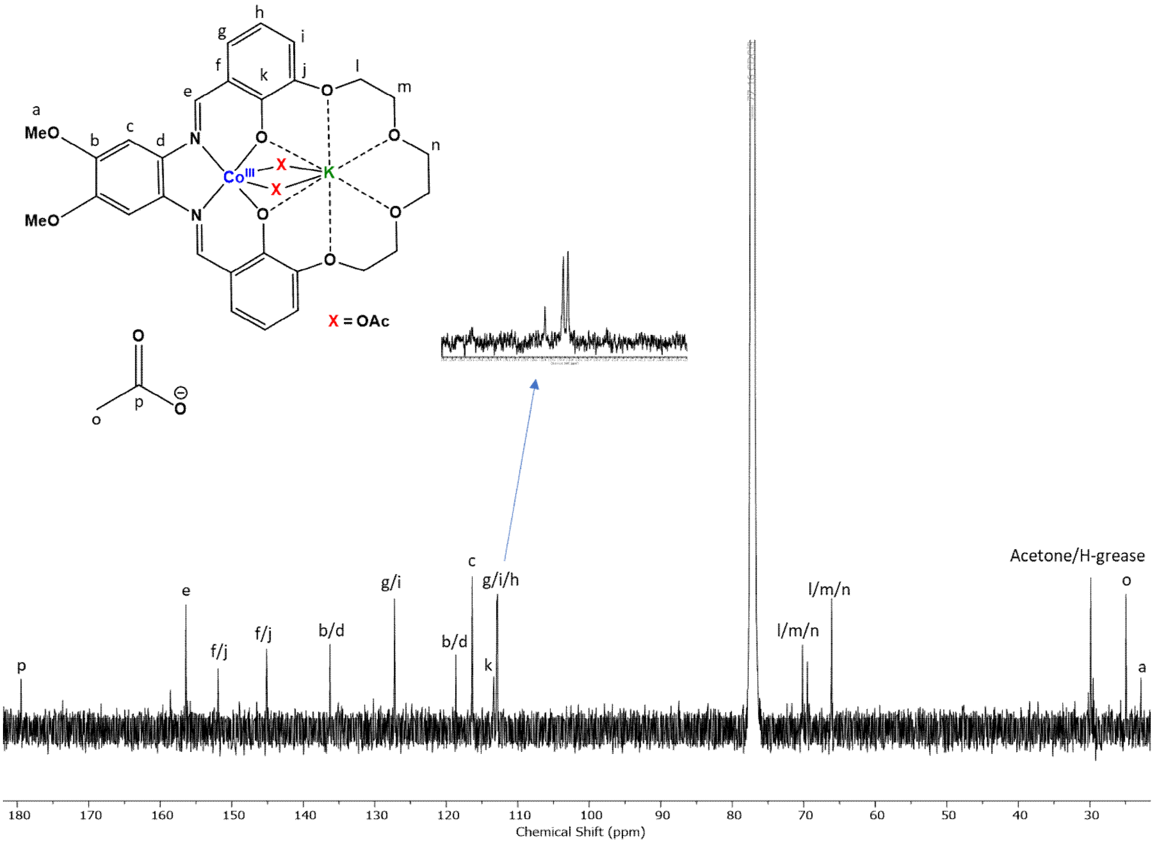

Figure S13 $^{13}C\{^{1}H\}$ NMR Spectrum of $\mathbf{2_{OMe}}$ ($CDCl_3$, 298K).

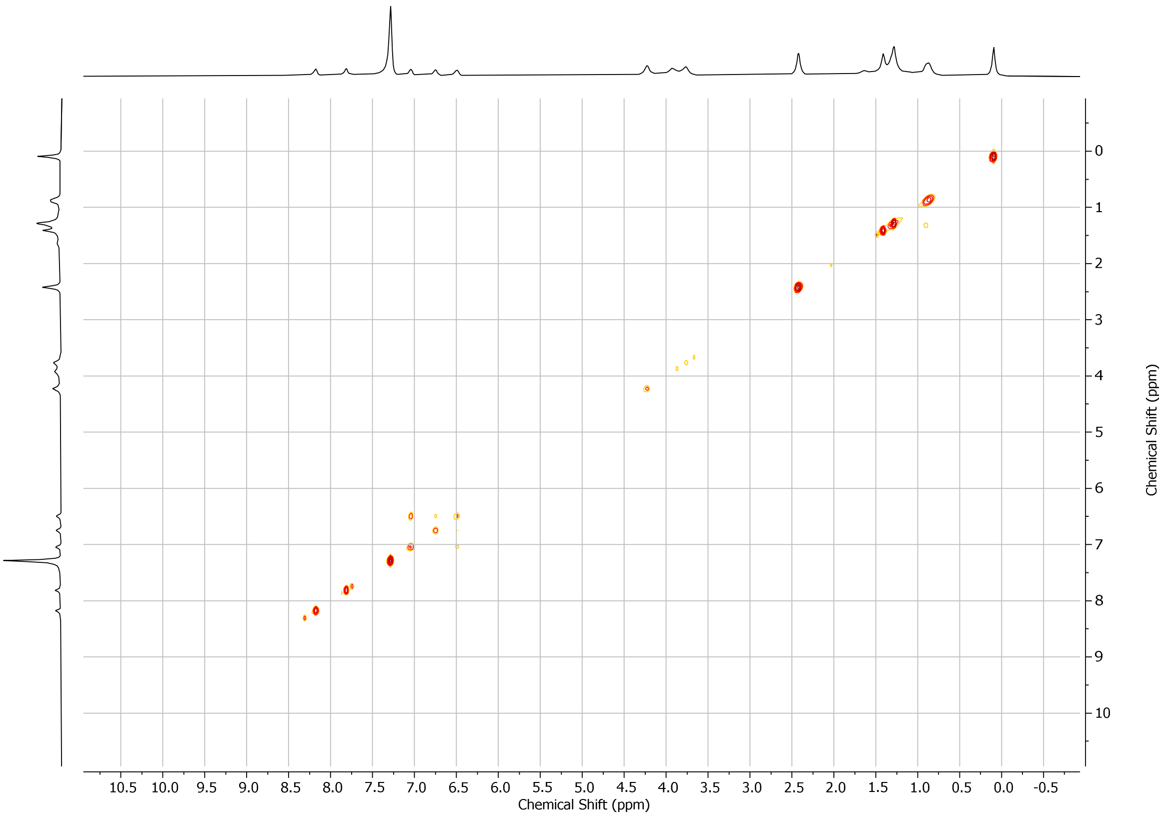

Figure S14 2D COSY NMR Spectrum of **2**$_{\mathbf{OMe}}$ ($CDCl_3$, 298K).

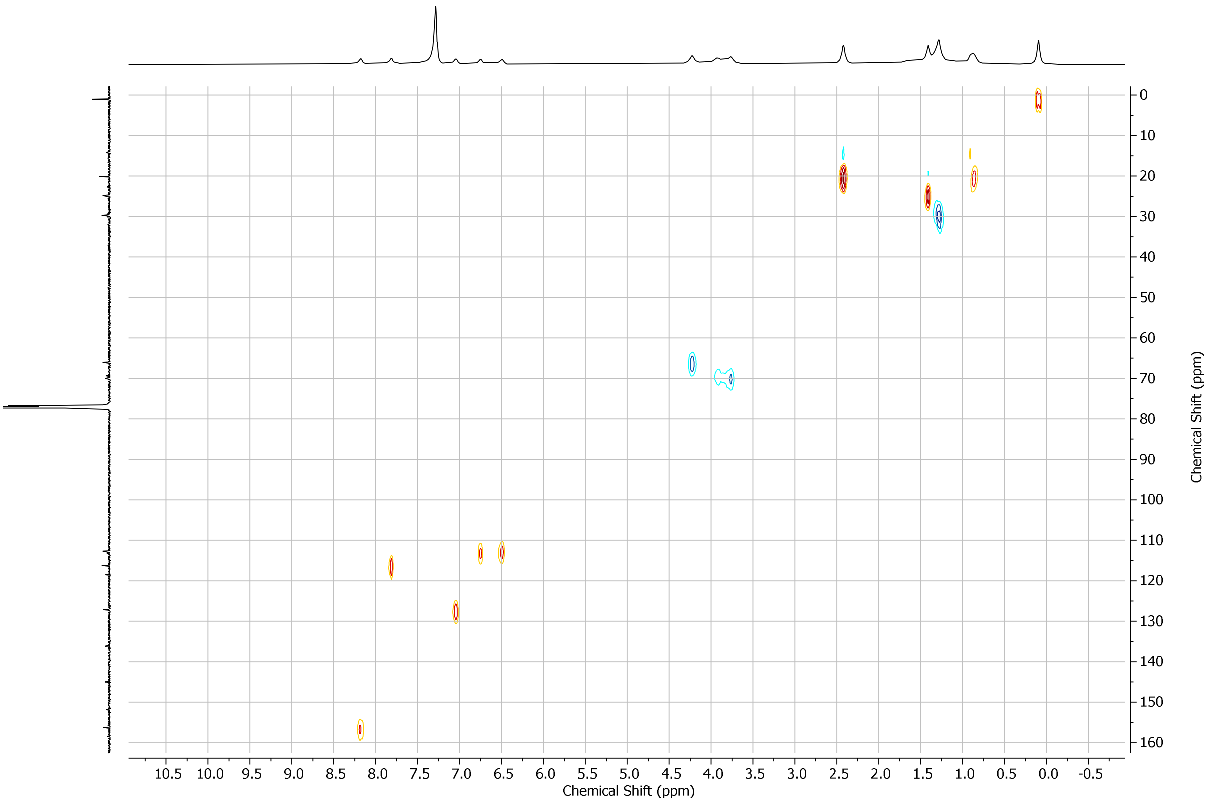

Figure S15 2D HSQC NMR Spectrum of **2**$_{\mathbf{OMe}}$ ($CDCl_3$, 298K).

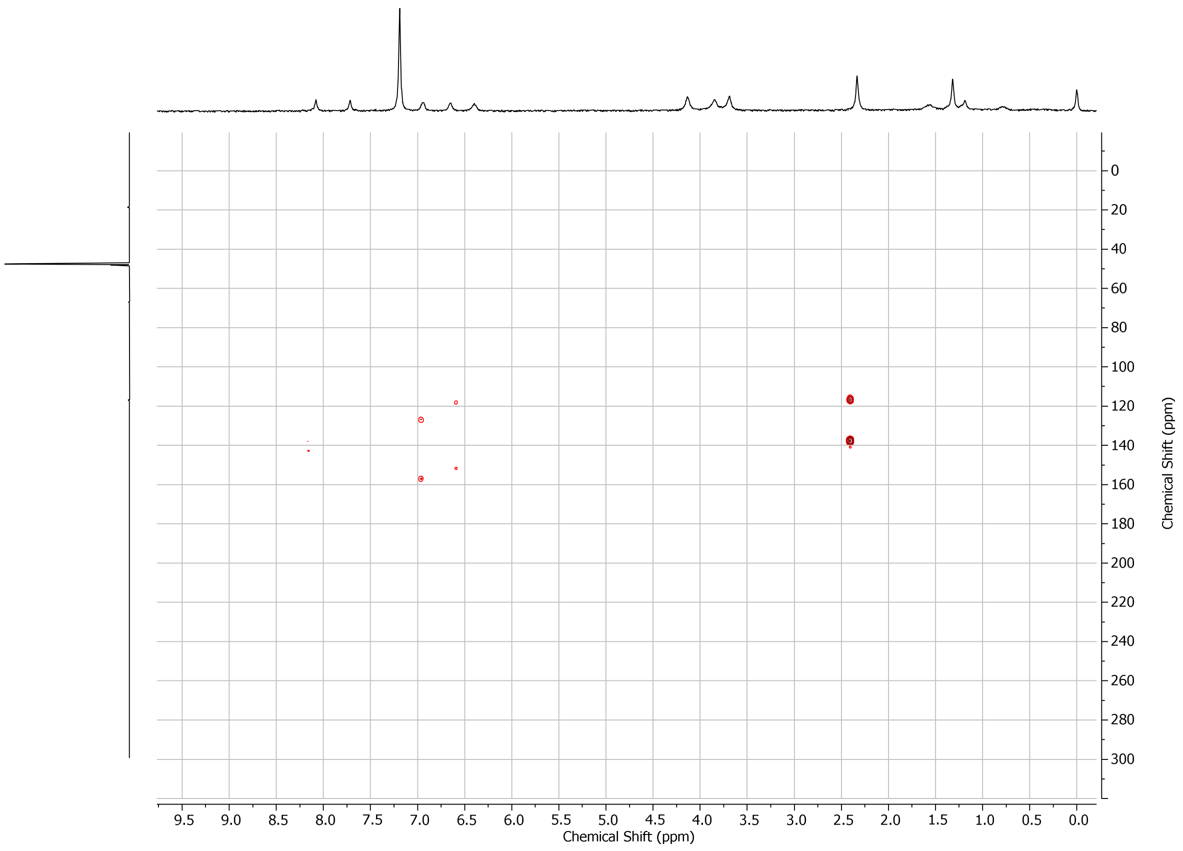

Figure S16 2D HMBC NMR Spectrum of $\mathbf{2}_{\mathbf{OMe}}$ ($CDCl_3$, 298K).

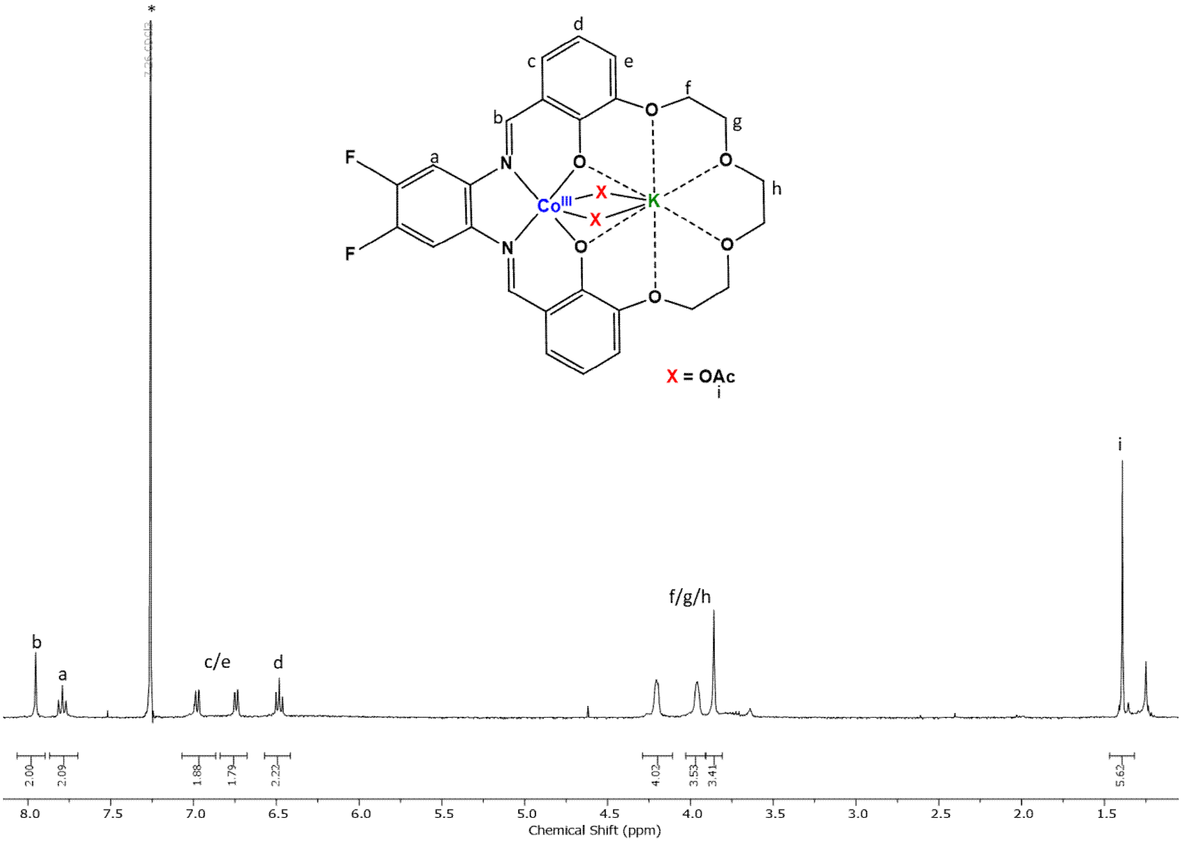

Figure S17 ^{1}H NMR Spectrum of $\mathbf{2}_{\mathbf{F}}$ ($CDCl_3$, 298K).

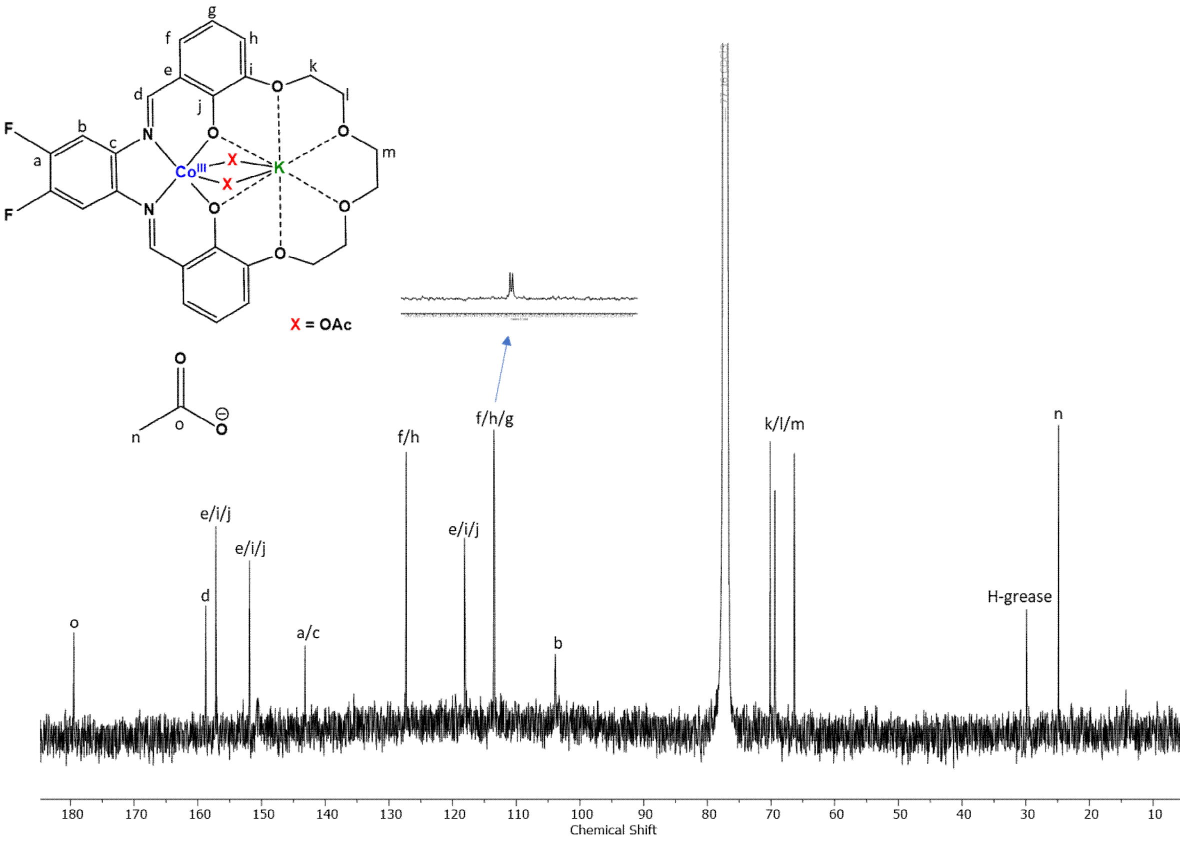

Figure S18 $^{13}C\{^{1}H\}$ NMR Spectrum of $\mathbf{2_F}$ ($CDCl_3$, 298K).

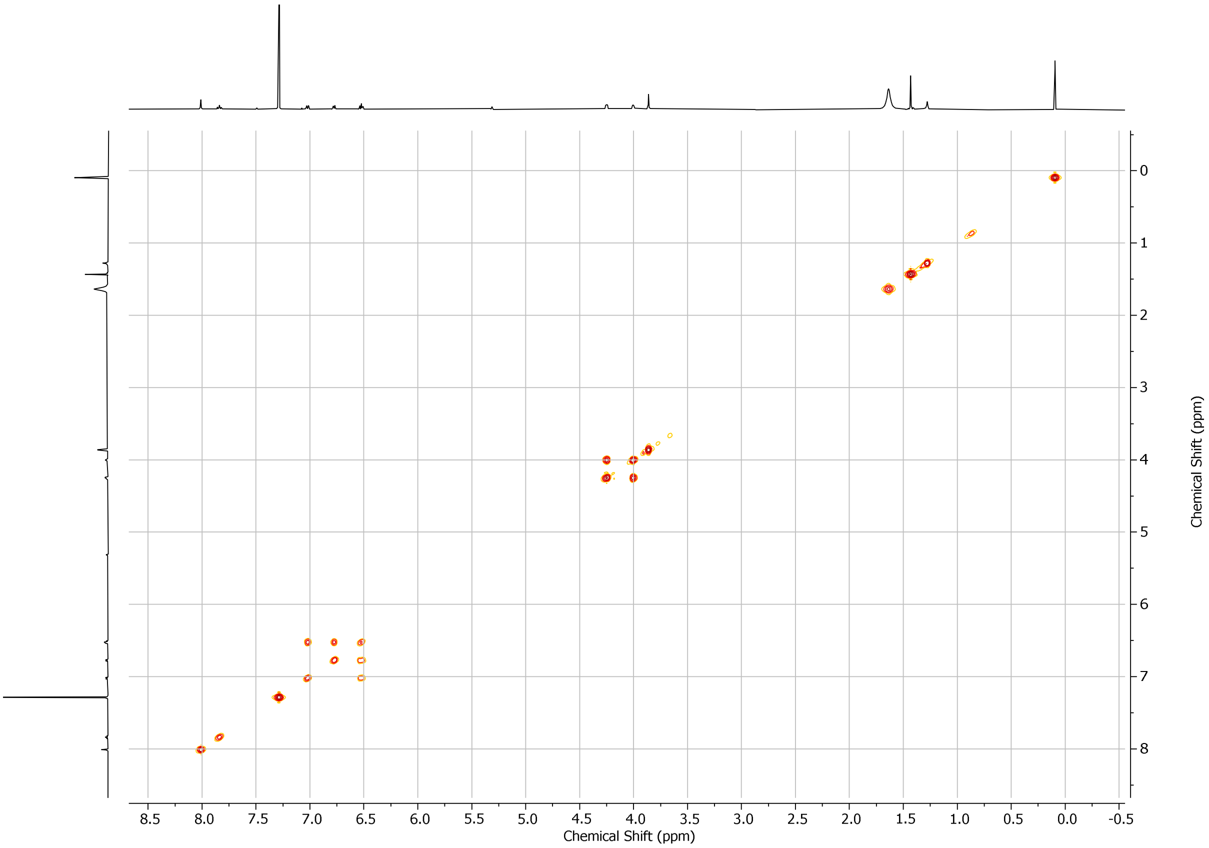

Figure S19 2D COSY NMR Spectrum of $\mathbf{2_F}$ ($CDCl_3$, 298K).

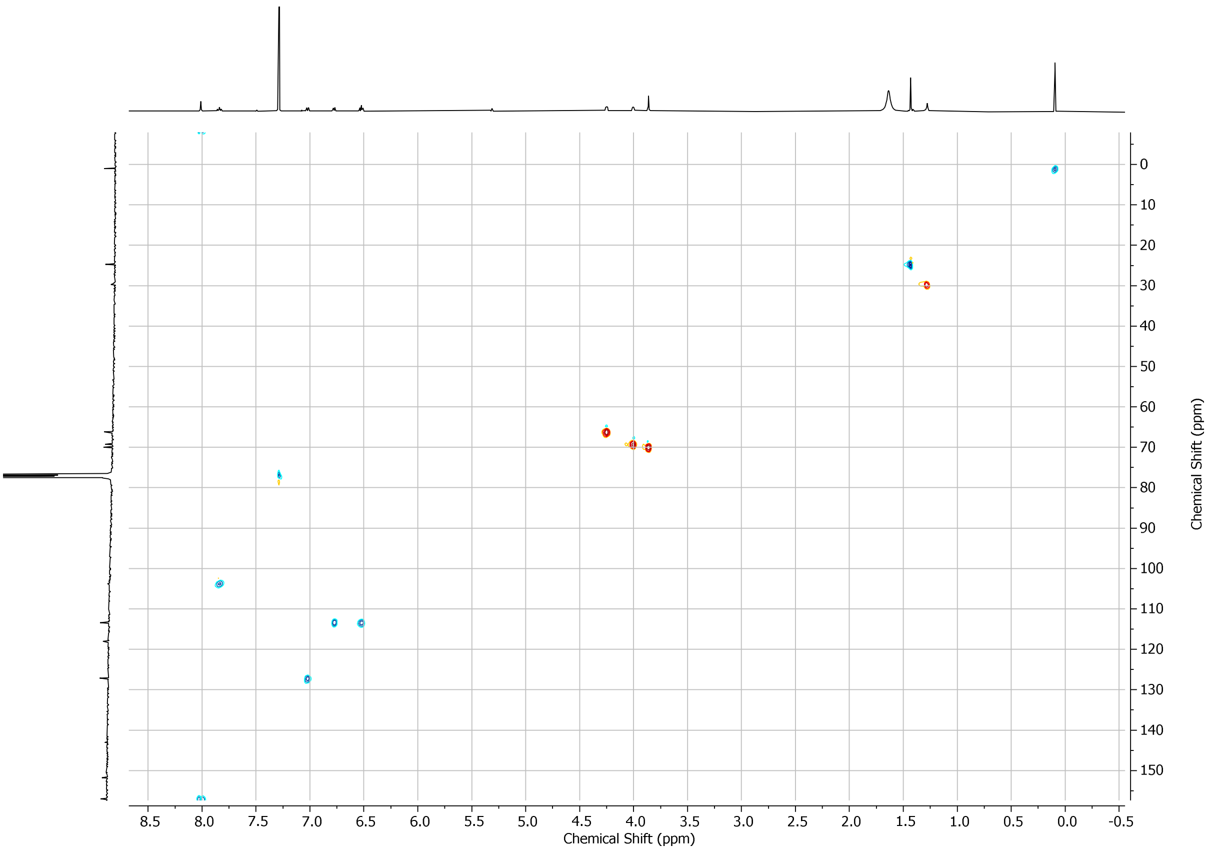

Figure S20 2D HSQC NMR Spectrum of $\mathbf{2_F}$ ($CDCl_3$, 298K).

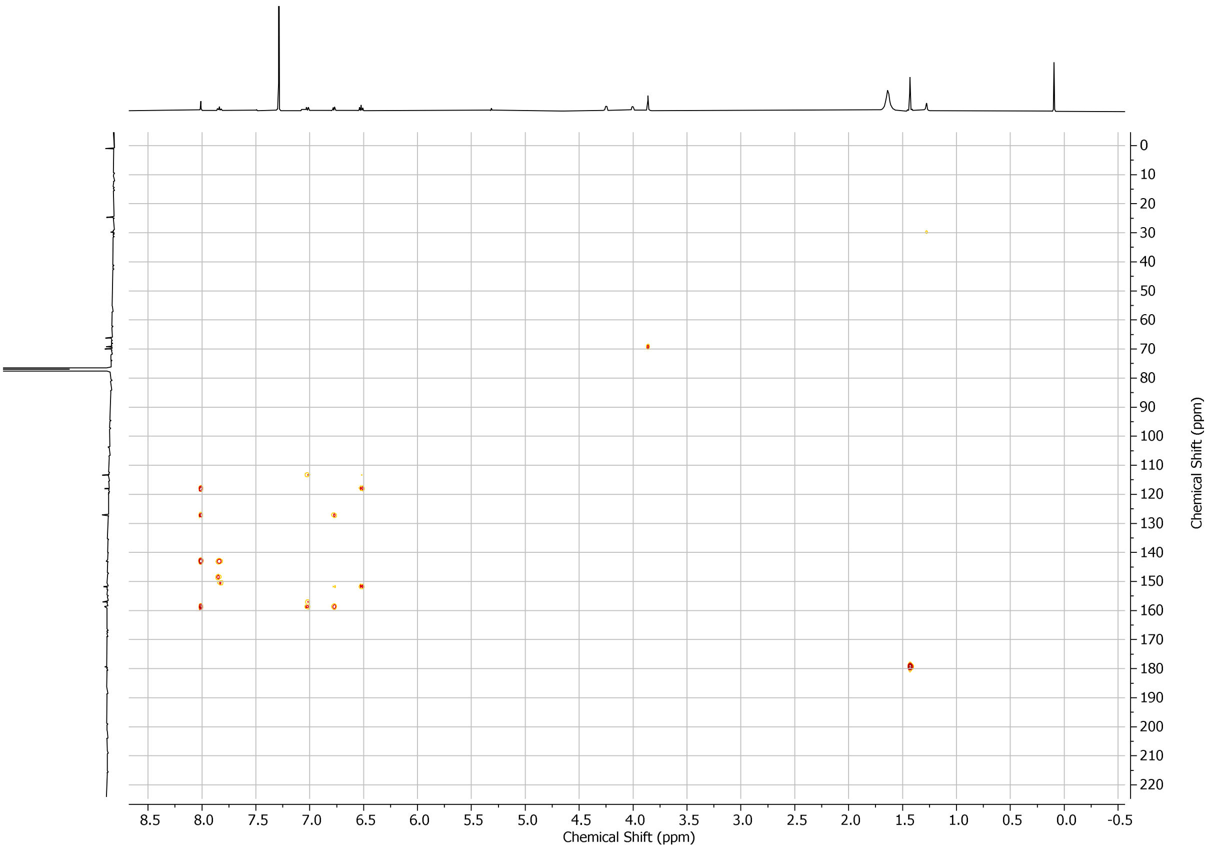

Figure S21 2D HMBC NMR Spectrum of $\mathbf{2_F}$ ($CDCl_3$, 298K).

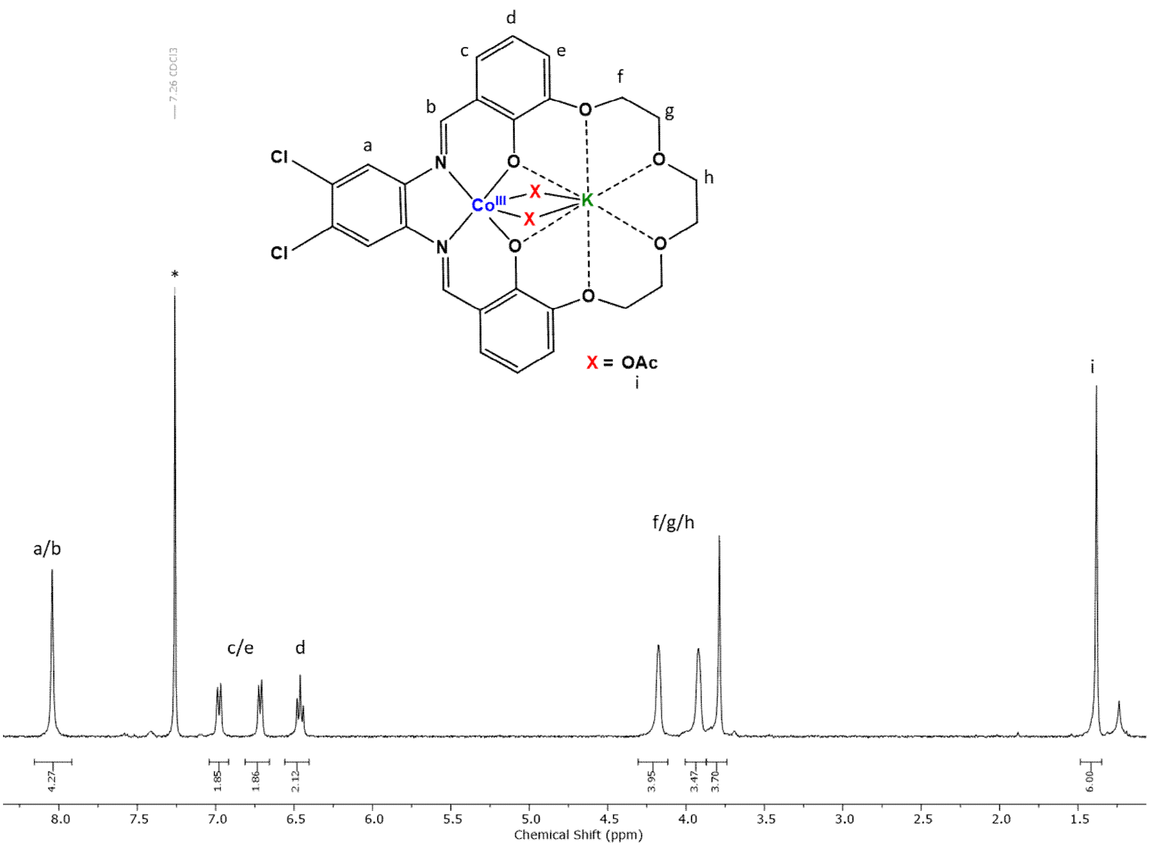

Figure S22 ^{1}H NMR Spectrum of $\mathbf{2}_{Cl}$ ($CDCl_3$, 298K).

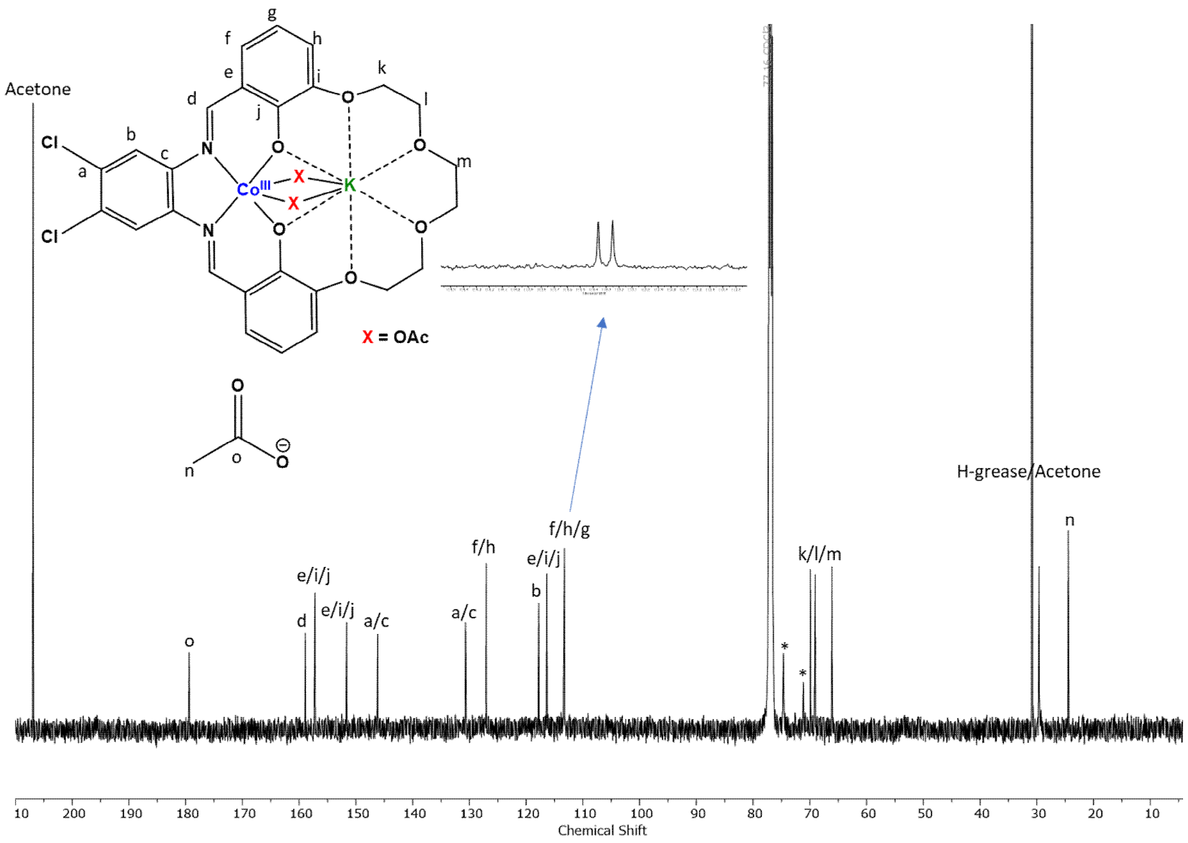

Figure S23 $^{13}C\{^{1}H\}$ NMR Spectrum of $\mathbf{2}_{Cl}$ ($CDCl_3$, 298K).

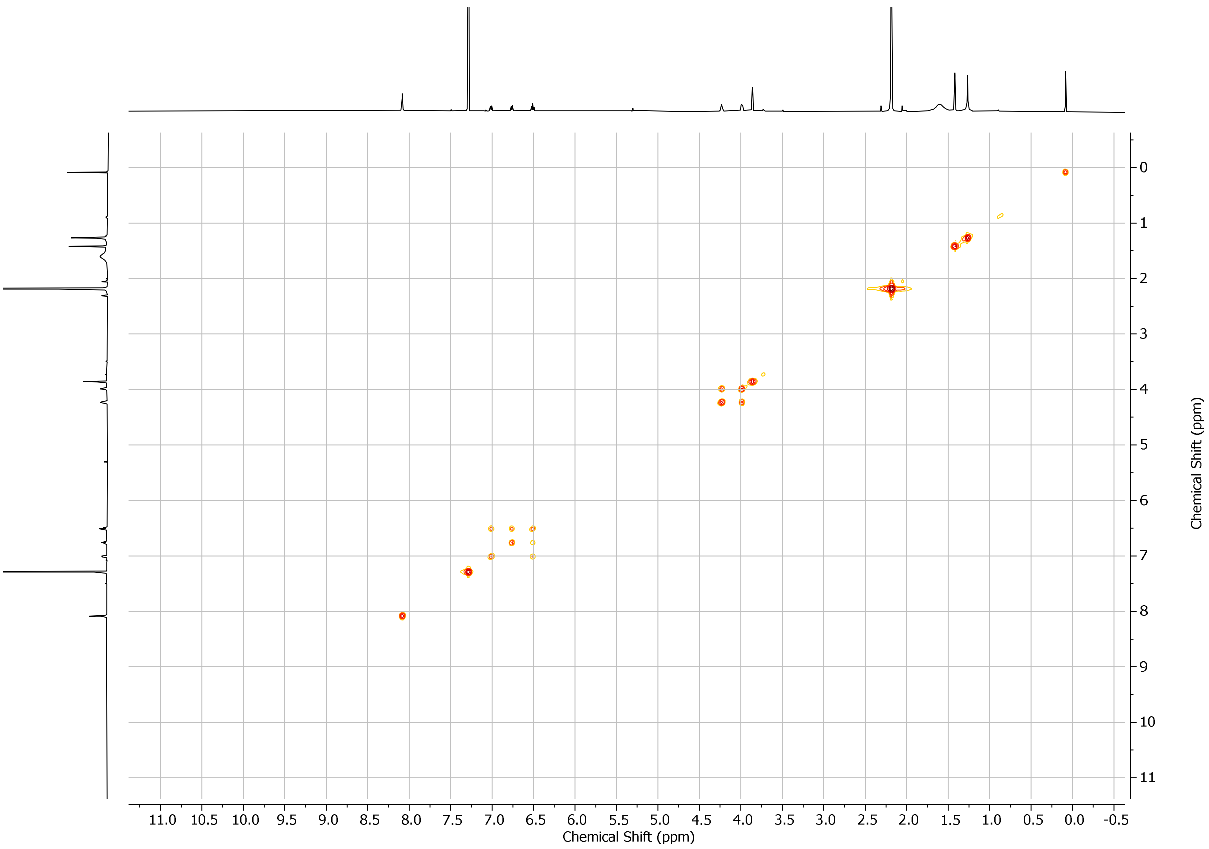

Figure S24 2D COSY NMR Spectrum of $\mathbf{2}_{Cl}$ ($CDCl_3$, 298K).

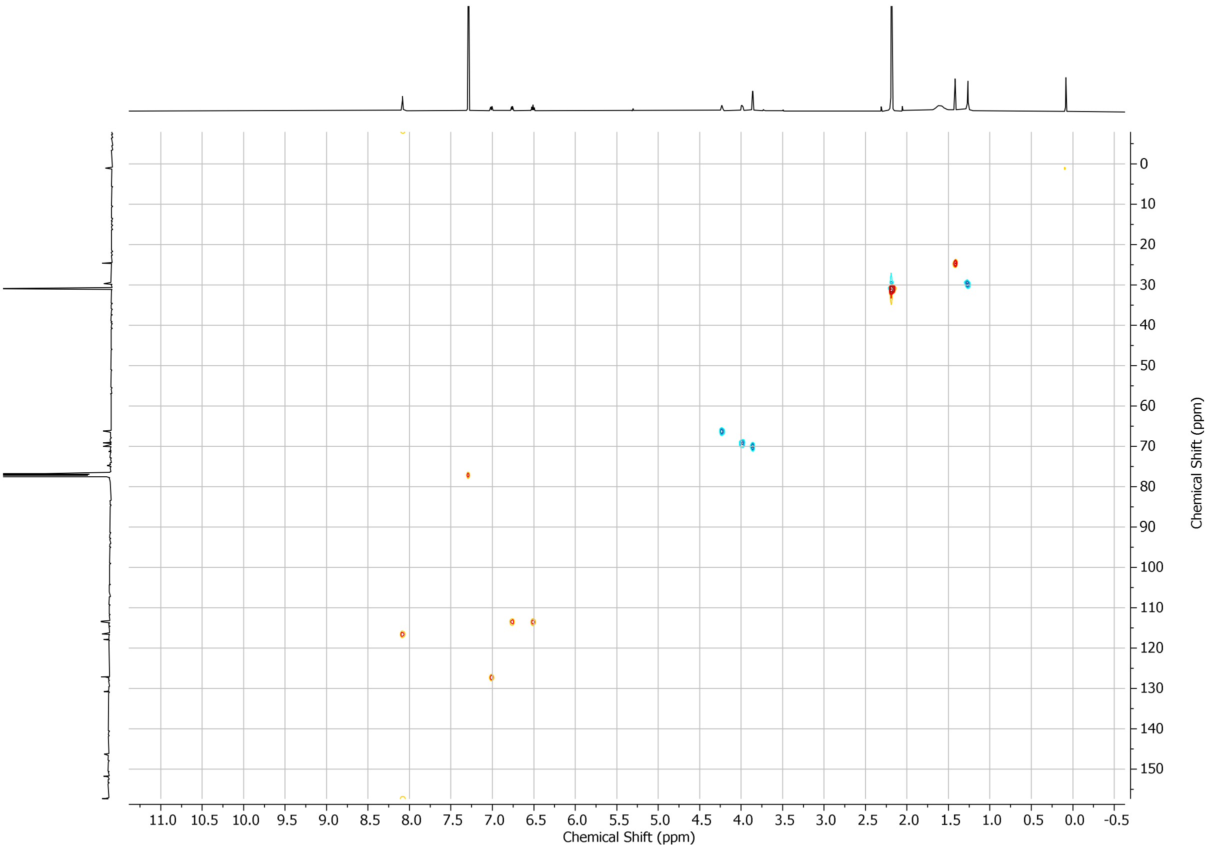

Figure S25 2D HSQC NMR Spectrum of $\mathbf{2}_{Cl}$ ($CDCl_3$, 298K).

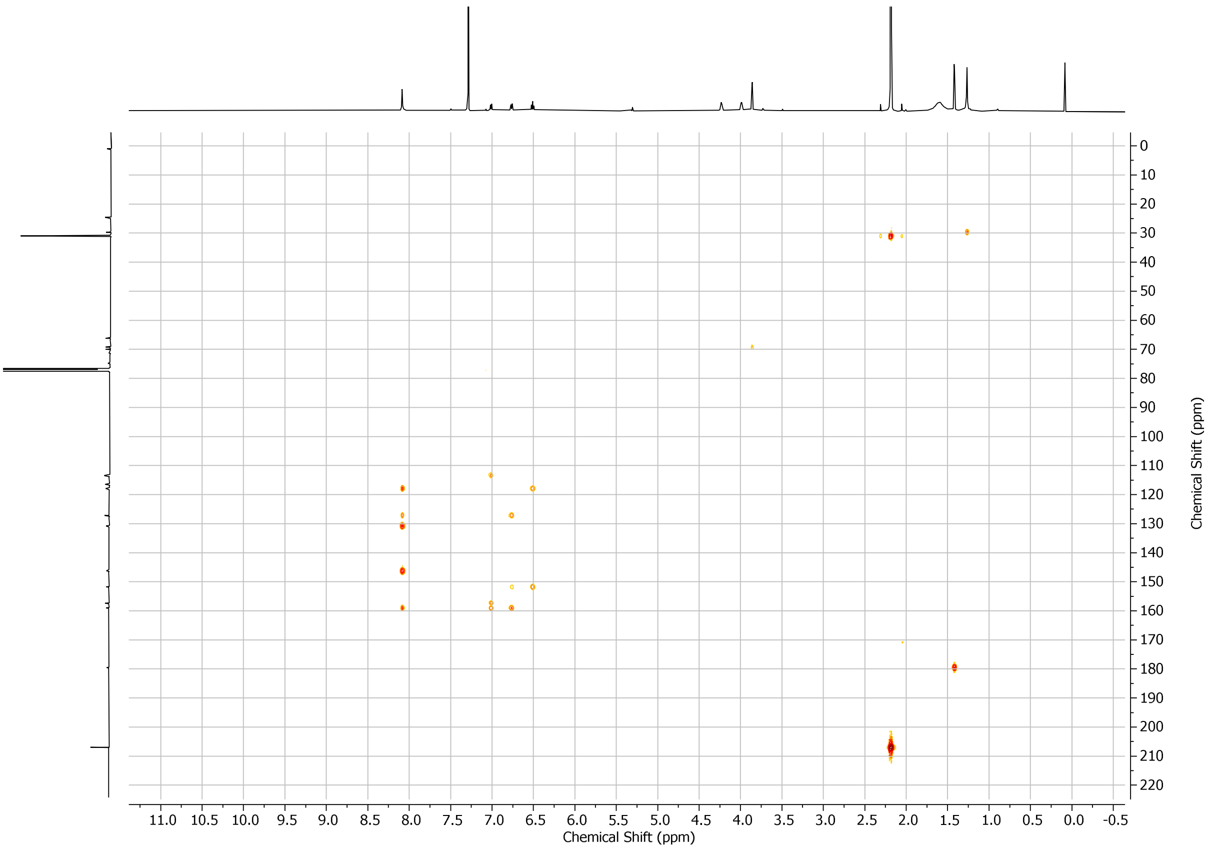

Figure S26 2D HMBC NMR Spectrum of **2$_{Cl}$** ($CDCl_3$, 298K).

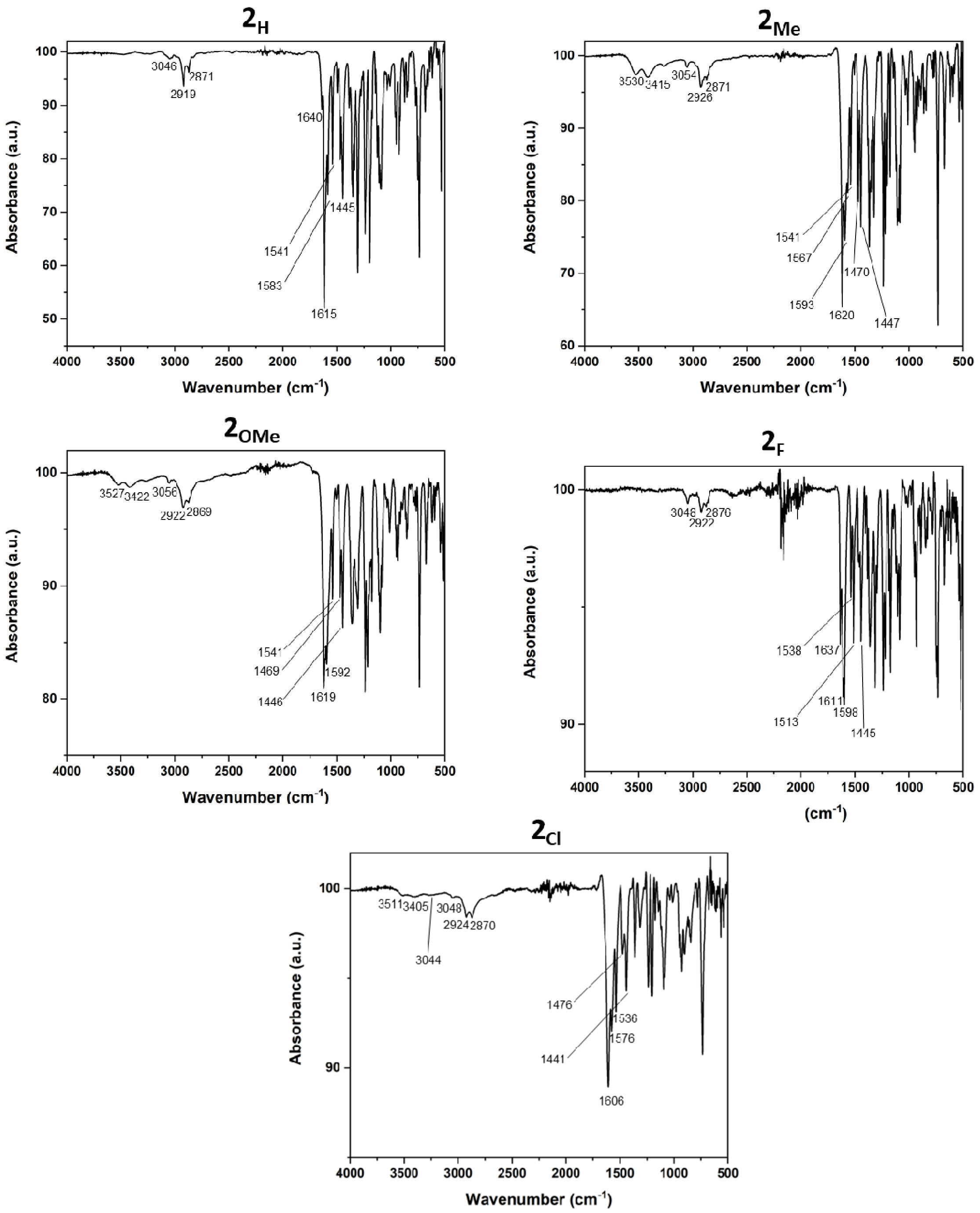

Figure S27 IR Spectra of Complexes $\mathbf{2_R}$.

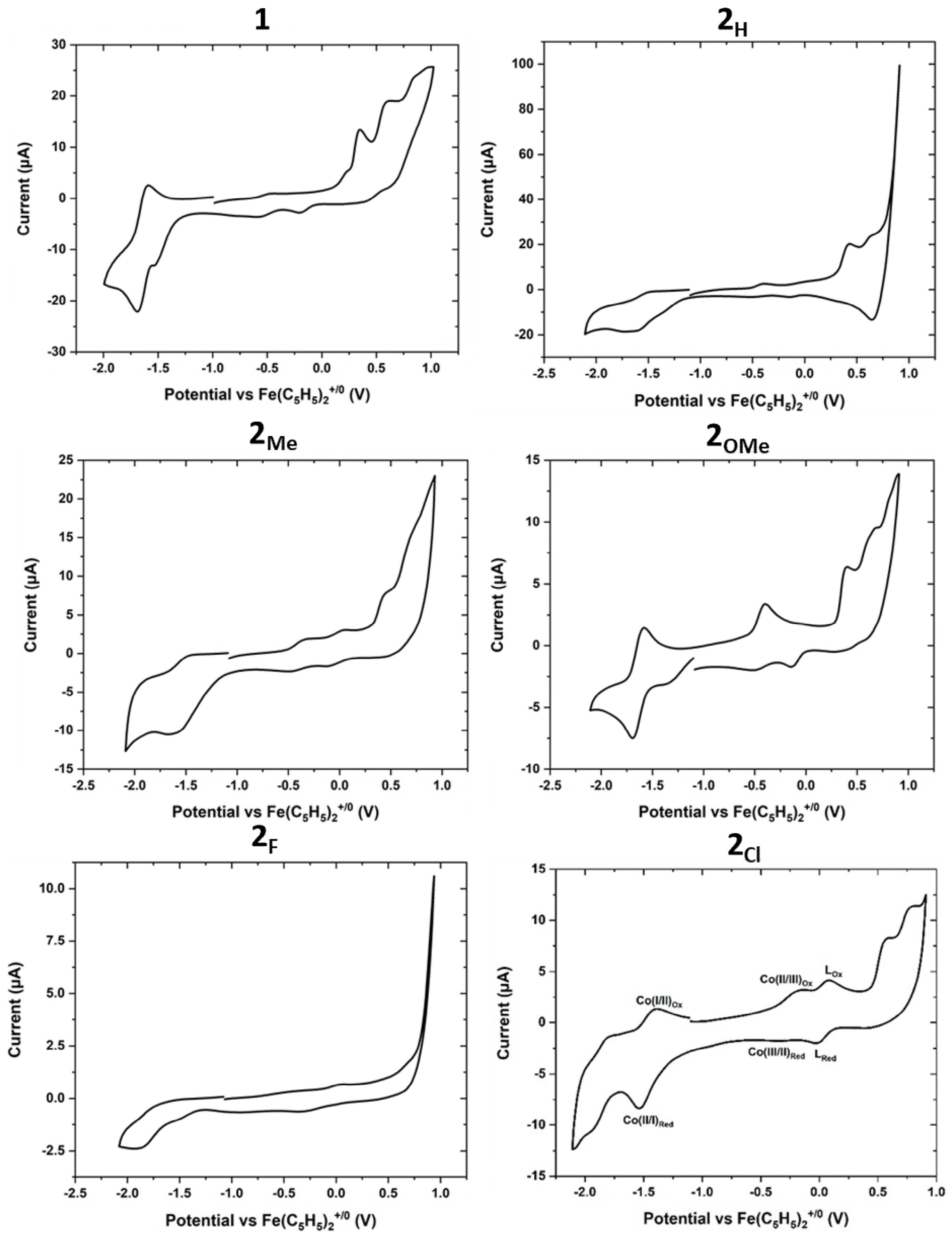

Figure S28 Cyclic Voltammetry Data of Complexes $\mathbf{2_R}$.

Table S2 Summary of Electrochemical Data for Complexes **1**-**2**$_{\mathbf{R}}$.[a]

Cat.	E_{Red} Co(II/I) (V)	$E_{1/2}$ Co(II/I) (V)	$E_{1/2}$ Co(III/II) (V)
**1**	-1.68	-1.65	-0.53
**2**$_{\mathbf{Me}}$	-1.61	-1.52	-0.37
**2**$_{\mathbf{OMe}}$	-1.69	-1.64	-0.41
**2**$_{\mathbf{H}}$	-1.72	-1.65	-0.48
**2**$_{\mathbf{F}}$	-1.55	-	-0.16
**2**$_{\mathbf{Cl}}$	-1.54	-1.49	-0.24

[a]Reduction potentials in acetonitrile referenced to ferrocene/ferrocenium.

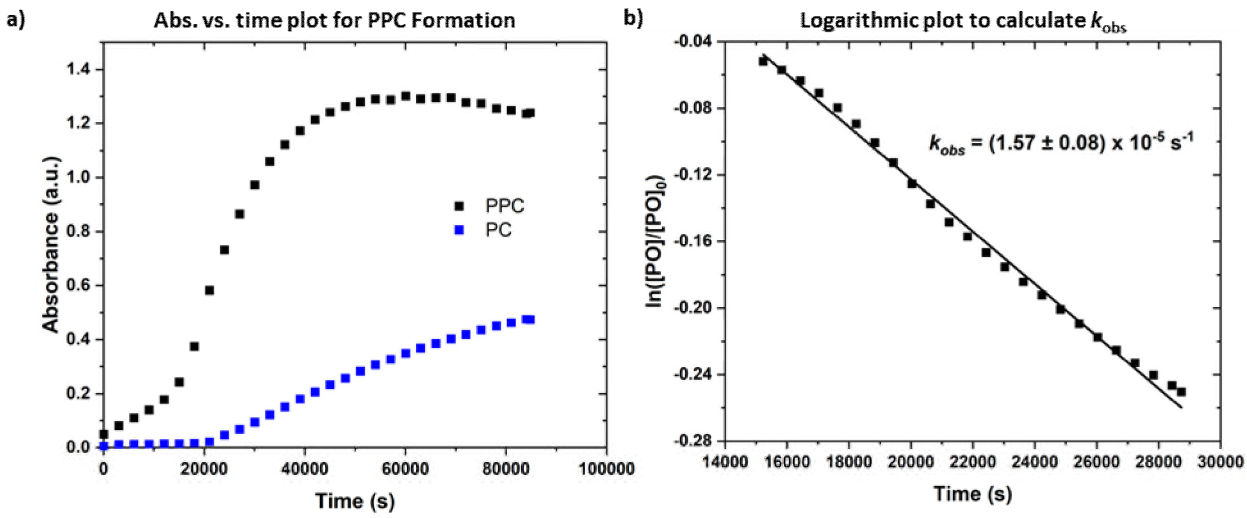

Figure S29 a) Absorbance versus time plot for PPC formation for **2**$_{\mathbf{Me}}$. b) Semi-logarithmic concentration vs. time plot, used to calculate k_{obs} for **2**$_{\mathbf{Me}}$.

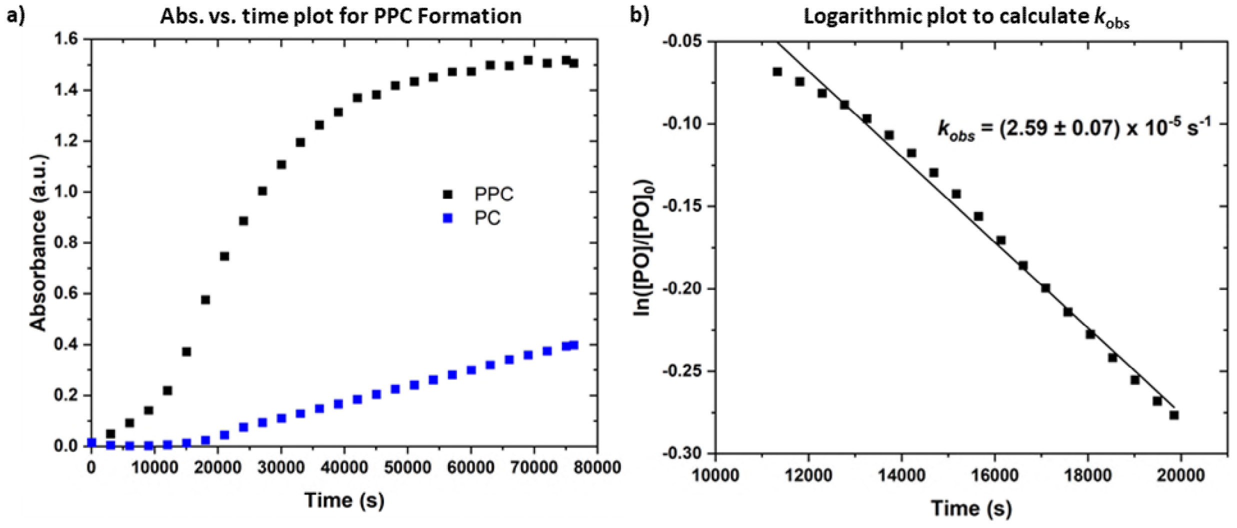

Figure S30 a) Absorbance versus time plot for PPC formation for **2**$_{\mathbf{OMe}}$. b) Semi-logarithmic concentration vs. time plot, used to calculate k_{obs} for **2**$_{\mathbf{OMe}}$.

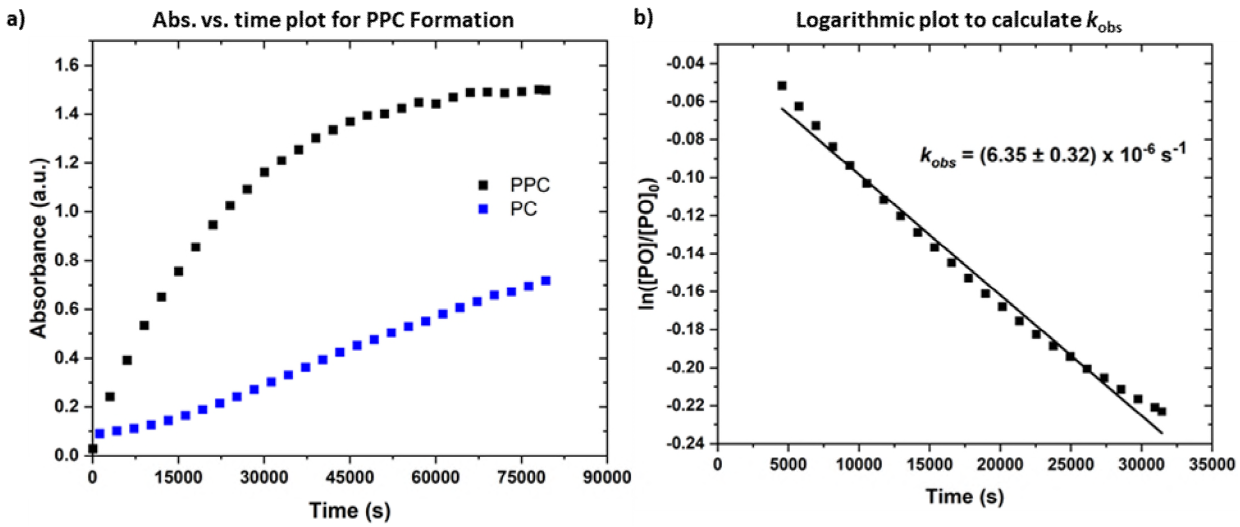

Figure S31 a) Absorbance versus time plot for PPC formation for $\mathbf{2_F}$. b) Semi-logarithmic concentration vs. time plot, used to calculate k_{obs} for $\mathbf{2_F}$.

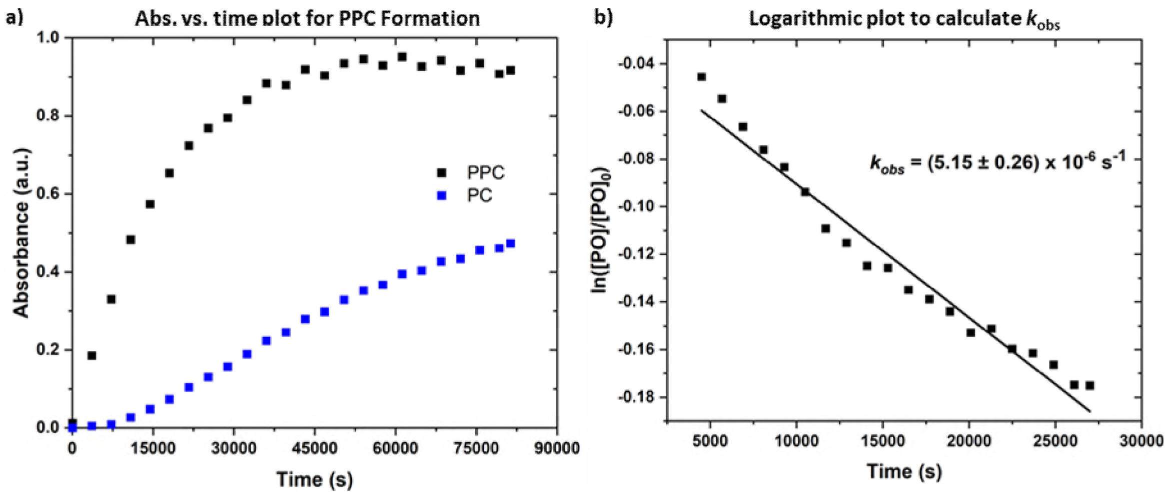

Figure S32 a) Absorbance versus time plot for PPC formation for $\mathbf{2_{Cl}}$. b) Semi-logarithmic concentration vs. time plot, used to calculate k_{obs} for $\mathbf{2_{Cl}}$.

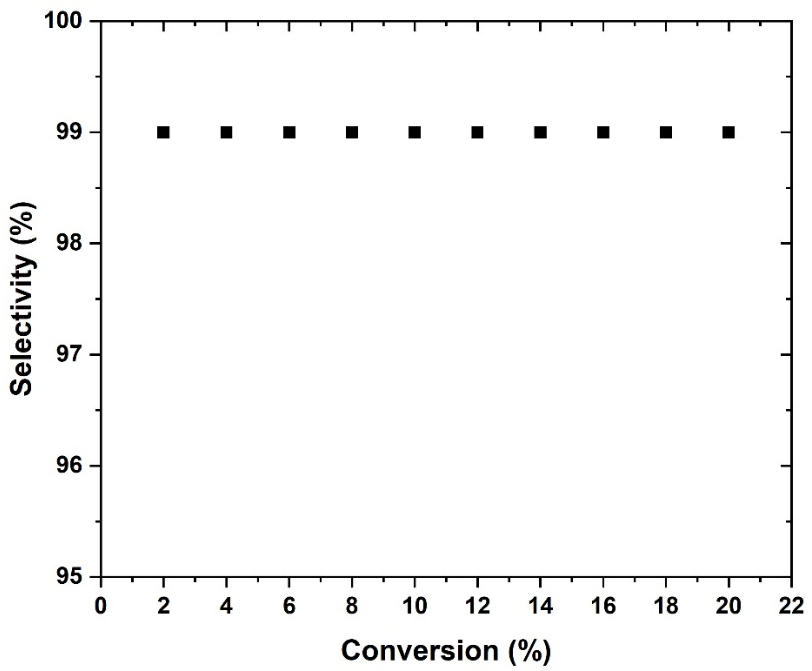

Figure S33 Conversion vs. Selectivity plot of PO/CO_2 ROCOP using $\mathbf{2_H}$ (0-20% conversion).

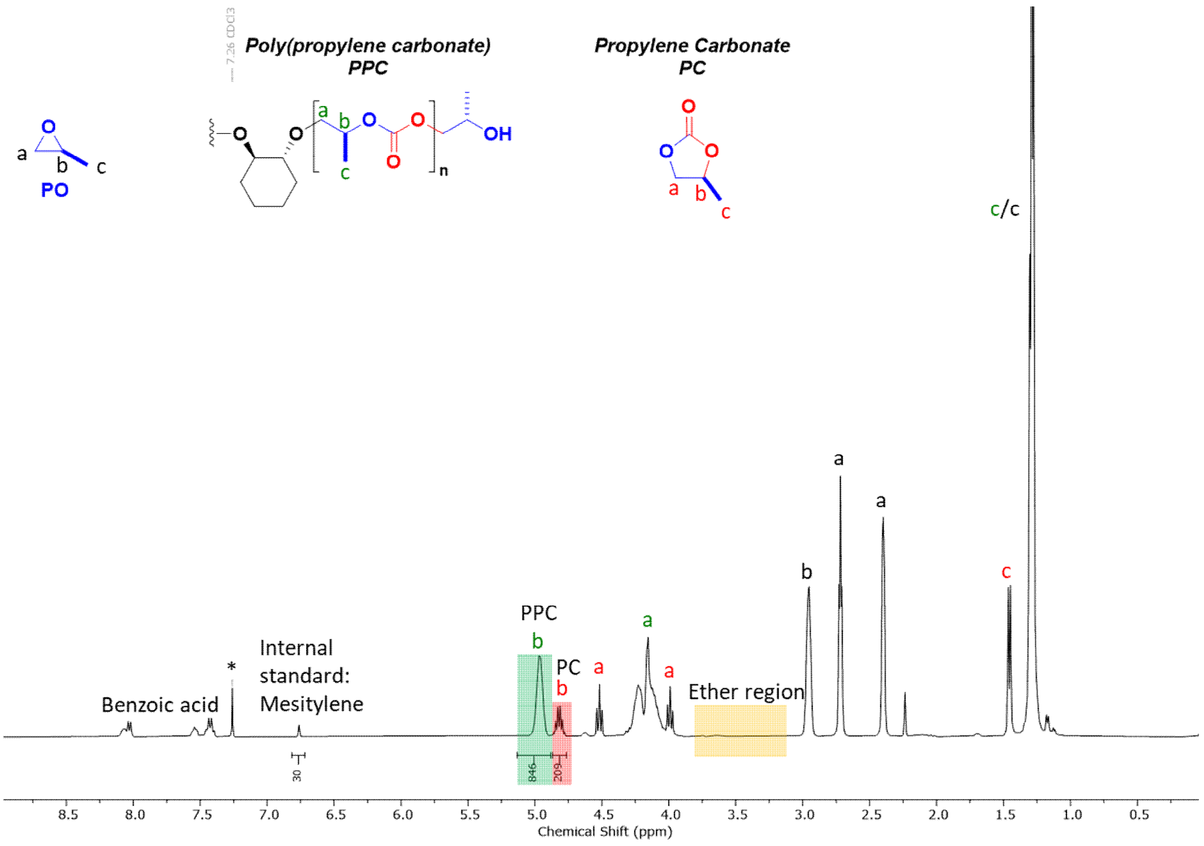

Figure S34 Exemplar ^{1}H NMR Spectrum of an aliquot removed from PO/CO_2 ROCOP using **2**, used to determine conversion and selectivity.

Table S3 CO_2/PO ROCOP Data Using Catalysts **1**-**2**$_R$.[a]

Cat.	Time (h)	Conv. (%)[b]	CO_2 Selec. (%)[c]	PPC Selec. (%)[c]	TON[d]	TOF_{PPC} (h^{-1})[e]	$k_{p,\ PPC}$ *10^3 (dm^3 mol^{-1} s^{-1})[f]	M_n [Đ] (g mol^{-1})[g]
**1**[h]	4.0	34	>99	98	1360 ± 68	333 ± 17	11.20 ± 0.56	5300 [1.08]
**2**$_H$	2.5	24	>99	>99	973 ± 49	389 ± 19	9.40 ± 0.47	4000 [1.06]
**2**$_{Me}$	11.7	34	>99	85	1402 ± 70	101 ± 6	4.40 ± 0.22	5500 [1.15]
**2**$_{OMe}$	7.5	37	>99	96	1494 ± 75	191 ± 10	7.25 ± 0.36	8900 [1.11]
**2**$_F$	13	30	>99	80	1196 ± 60	73 ± 4	1.78 ± 0.09	4400 [1.11]
**2**$_{Cl}$	12	24	>99	75	962 ± 48	62 ± 3	1.44 ± 0.07	4200 [1.13]

[a]Reaction conditions: Catalyst (0.025 mol %, 3.5 mM), PO (6 mL, 14 M), 1,2-cyclohexanediol (0.5 mol %, 70 mM), 20 bar CO_2, 50 °C. All data and errors from experiments run in triplicate. [b]PO conversion determined from the relative integrals in the ^{1}H NMR spectrum of PPC (4.92 ppm, 1H), CC (4.77 ppm, 1H), and PPO (3.46−3.64 ppm, 3H) using mesitylene as an internal standard (6.70 ppm). [c]PPC selectivity determined by the relative integrals in the ^{1}H NMR spectrum of PPC (4.92 ppm, 1H) compared with PC (4.77 ppm, 1H) and PPO (3.46−3.64 ppm, 3H). [d]Turnover number (TON) = number of moles of PO consumed/ number of moles catalyst. [e]Turnover frequency of PPC (TOF_{PPC}) = (TON*PPC selectivity)/time (h). [f]$k_p = k_{obs}/[cat]^1$; k_{obs} determined as the gradient of the plot of $\ln[PO]_t/[PO]_0$ vs time over the conversion range of 10% to 20%. [g]Determined by GPC analysis, in THF, calibrated with narrow-M_n polystyrene standards; dispersity values in parentheses. [h]Previously reported.[1]

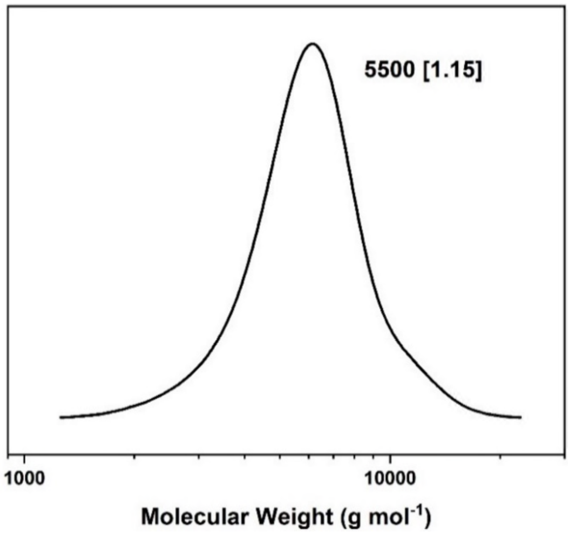

Figure S35 GPC trace of PO/CO_2 ROCOP for **2**$_{Me}$ (Table 1).

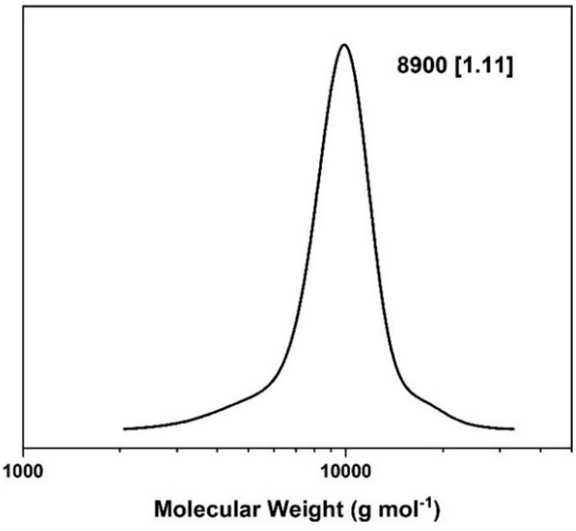

Figure S36 GPC trace of PO/CO_2 ROCOP for **2**$_{OMe}$ (Table 1).

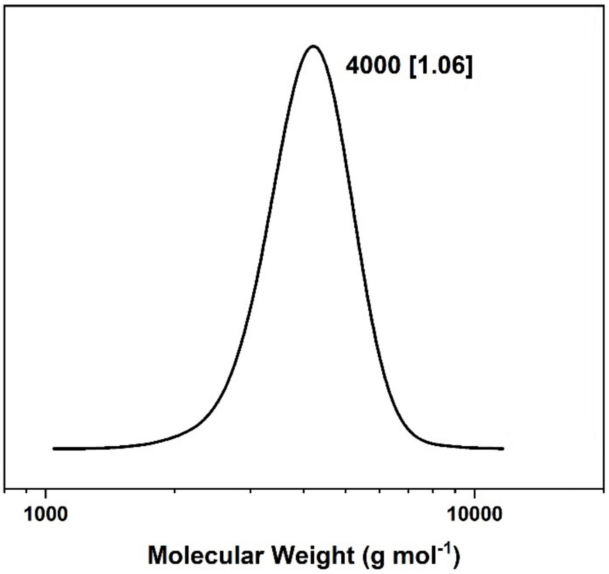

Figure S37 GPC trace of PO/CO_2 ROCOP for $\mathbf{2_H}$ (Table 1)

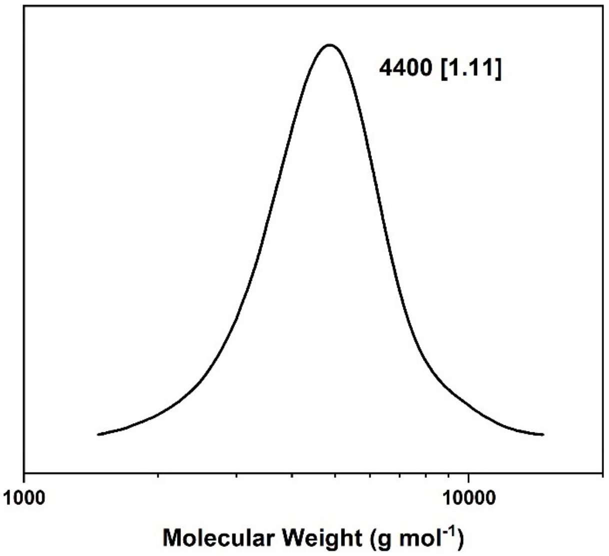

Figure S38 GPC trace of PO/CO_2 ROCOP for $\mathbf{2_F}$ (Table 1).

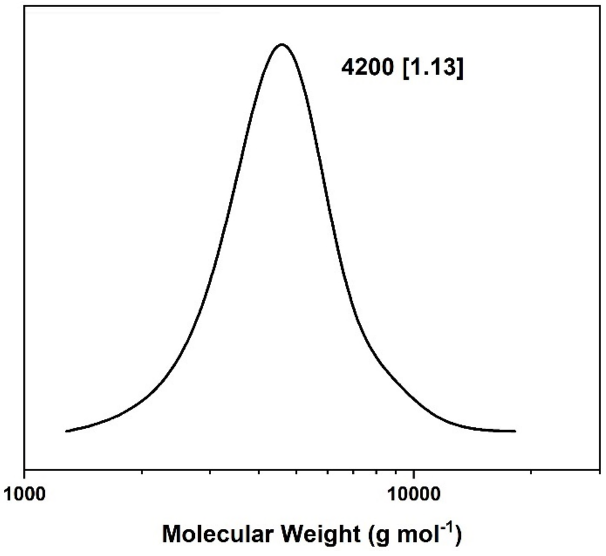

Figure S39 GPC trace of PO/CO_2 ROCOP for $\mathbf{2_{Cl}}$ (Table 1).

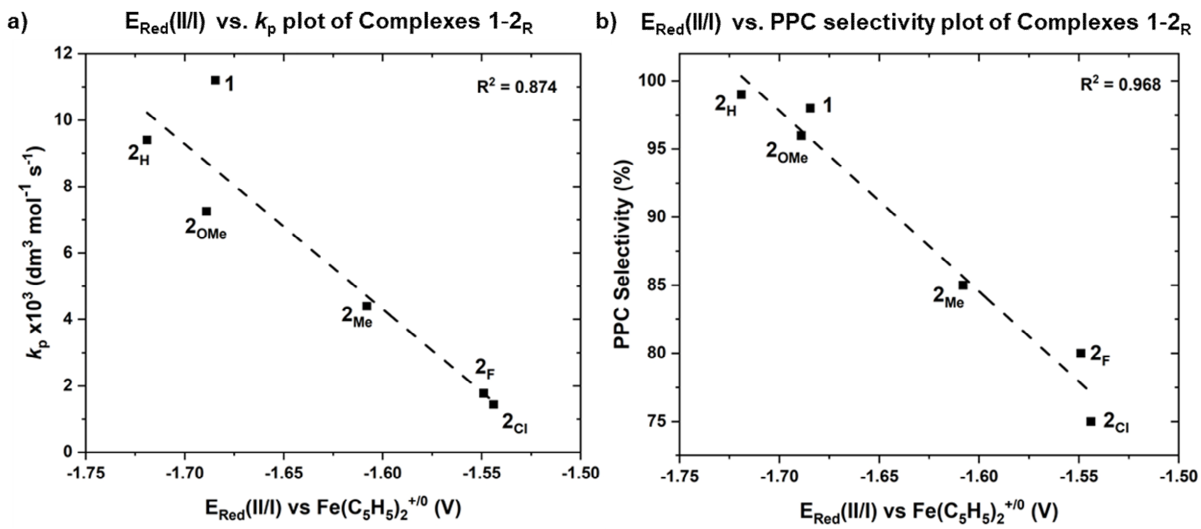

Figure S40 a) E_{Red}(II/I) vs. k_p plot of catalysts $\mathbf{2_R}$. b) E_{Red}(II/I) vs. PPC selectivity plot of catalysts $\mathbf{2_R}$.

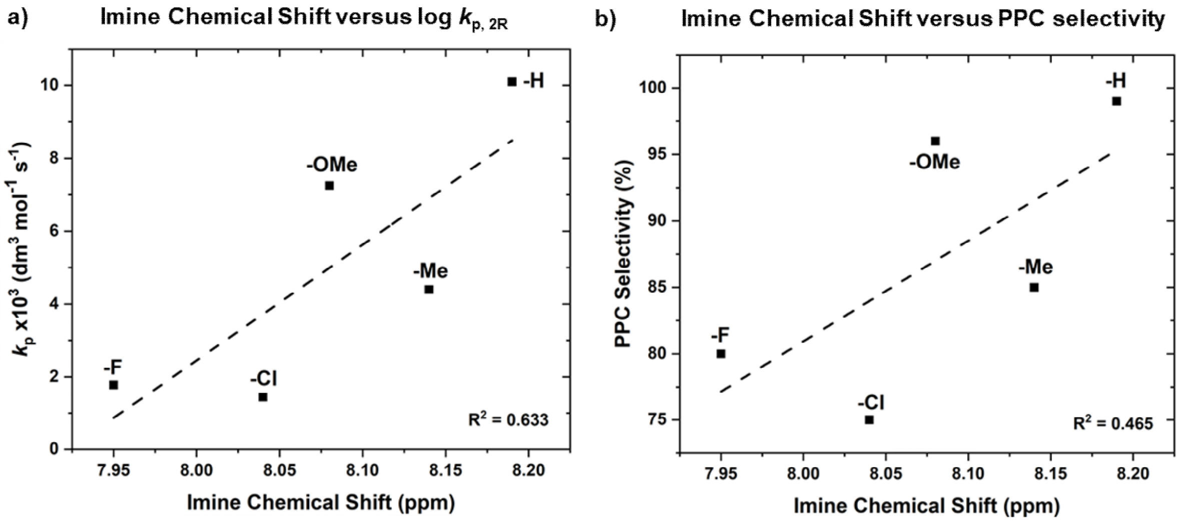

Figure S41 a) Imine Chemical Shift versus k_p. b) Imine Chemical Shift versus PPC selectivity.

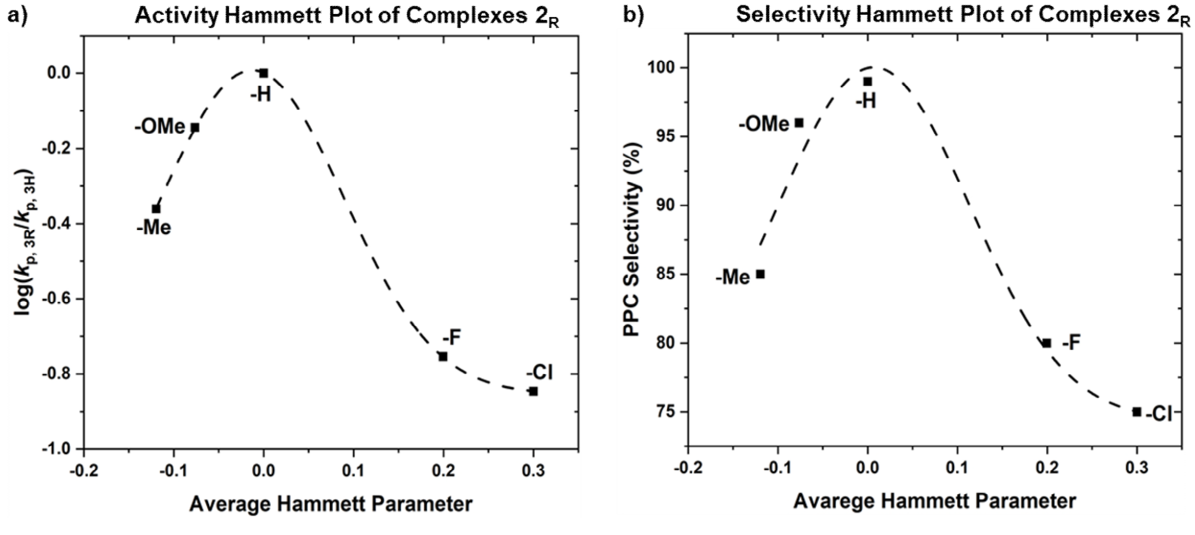

Figure S42 a) Activity Hammett plot of complexes $\mathbf{2_R}$. b) Selectivity Hammett plot of complexes $\mathbf{2_R}$.

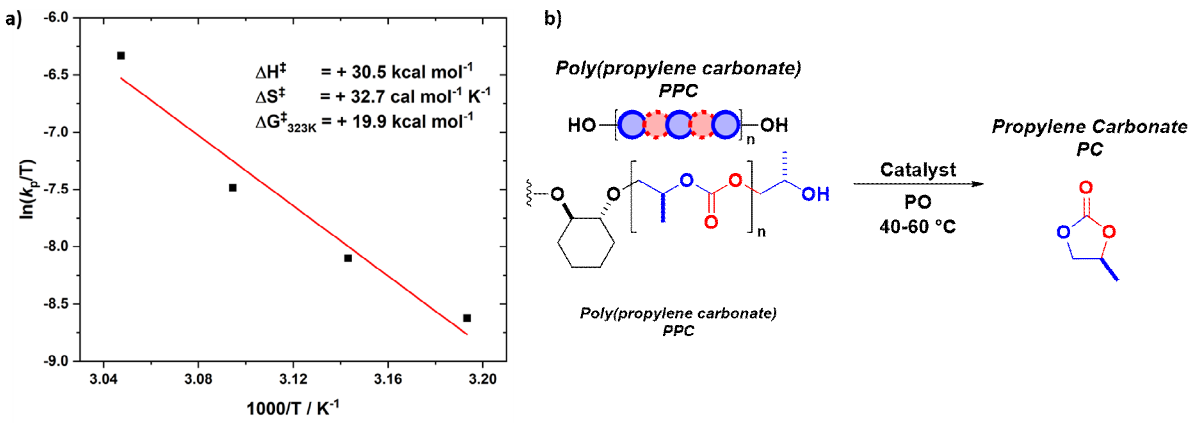

Figure S43 a) Eyring Plot for PPC backbiting to PC, using $\mathbf{2}_{Cl}$. Experiments were conducted from 45 to 60 °C, [$\mathbf{2}_{Cl}$] = 3 mM; $[PPC]_0$ = 300 mM, neat PO (14 M) and were analyzed to full conversion, where $[PPC]_0$ is the monomer repeat unit concentration. b) Reaction scheme illustrating conversion PPC to PC.

X-ray crystallography Experimental Section

Single crystal X-ray diffraction data were collected using a Rigaku Oxford Diffraction SuperNova in-house diffractometer, fitted with an Oxford CryoSystems 700 Series CryoStream.[2] Suitable crystals were chosen and mounted on a 200 µm MiTeGen loop, using perfluoropolyether oil (Fomblin®), at 298 K. Typically, a hemisphere of data was collected to a resolution of 0.8 Å at 150 K.

The CrysAlisPro software was used for data collection, as well as peak hunting, indexing reflections in reciprocal space, integration of the raw frames and application of corrections including interframe scaling, Lorentz, flood field and dark current corrections.

The structures were solved using the SHELXT program[3,4] and least-square refined using the SHELXL program[5] within the Olex2 system suite.[6]

X-ray crystal Data for $\mathbf{2}_H$

Crystal data for $\mathbf{2}_H$: $C_{30}H_{30}CoKN_2O_{10}$, 2(H_2O), M = 712.62, monoclinic, C12/c1 (no. 15), a = 12.7759(7), b = 16.4857(10), c = 15.3281(9) Å, α = 90, β = 108.438(6), γ = 90°, V = 3062.7(3) Å^3, Z = 4, D_c = 1.545 g cm^{-3}, μ(Cu-Kα) = 6.205 mm^{-1}, T = 150 K, clear brown blocks, Rigaku Oxford Diffraction SuperNova diffractometer; 3166 independent measured reflections (R_{int} = 0.0753), F^2 refinement, R_1(obs) = 0.0581, wR_2(all) = 0.1624, 2297 independent observed absorption-corrected reflections [$|F_o| > 4\sigma(|F_o|)$, completeness to θ_{full}(152.8°) = 99.9%], 213 parameters. CCDC 2222739.

The molecule sits across a plane of symmetry that bisects the molecule along the Co1 – K1 interatomic vector, giving 0.5 molecular equivalents per asymmetric unit cell. There is one molecule of H_2O per asymmetric unit which was freely refined anisotropically with associated riding protons that result in an H-bond to the coordinated acetate.

Density Functional Theory Calculations

Computational Methods

DFT calculations were run using Gaussian16 (Revision C.01).[7] The reaction free energies were calculated using the previously benchmarked level of theory for similar polymerisation processes.[8] The hybrid exchange-correlation ωB97X-D functional, which includes D2 dispersion corrections described by Grimme was used.[9,10] Reaction profiles are plotted using thermal parameters obtained directly from frequency calculations of optimised structures using basis set 1 (BS1), as well as from SCF energies obtained using basis set 2 (BS2) corrected using thermal parameters from BS1. NBO analysis was performed using NBO 6.0 with BS1.[11]

Basis set 1 (BS1) was constructed as follows. The split valence 6-31+g(d,p) basis sets were used for carbon and hydrogen. This lower basis set was chosen as these elements do not bind directly to either catalytic metal center, but extra diffuse functions were added to capture more mid- and long-range interactions, for instance with growing polymer chains. The

triple-ζ 6-311+g(d) basis set was used for potassium and all heteroatoms. Cobalt centers were described with the Stuttgart SDD ECP and associated basis sets.

Basis set 2 (BS2) was constructed as follows. Aldrich's quadruple-ζ basis set def2-QZVPP was used on all atoms without employing any ECPs for metal atoms.[12]

The geometry optimization calculations were performed without symmetry constraints and an improved numerical integration grid using a pruned grid with 99 radial shells and 590 angular points per shell ([int=ultrafine]) was used. It should be noted that the inclusion of empirical dispersion was deemed unnecessary due to the built-in dispersive corrections associated with the functional used. All structures are optimized using the self-consistent reaction field (SCRF) approach with conductor-like polarisable continuum model (CPCM).[13] In polymerization reactions, the propene oxide (PO) monomer also serves as the solvent but is not implemented as a standard solvent in Gaussian16. A CPCM model for PO was therefore implemented by using the built-in non-polar parameters of tetrahydrofuran, modified with the polar parameters for PO (ε = 16, n^2 = 1.867) ([SCRF=(cpcm,solvent=tetrahydrofuran),read] and [eps=16/epsinf=1.867]).

Free enthalpies were corrected by two methods: i) more crudely using the built-in Gaussian16 utility *freqchk* to adjust calculated frequency and thermochemistry data using a temperature of 323.15 K, a pressure of 19.74 atm (20 bar), and a frequency scale factor = 1.0; ii) more accurately using the Goodvibes software,[14] with Grimme's quasiharmonic approximation applied with a frequency cut-off values of 100.0 wavenumbers and a frequency scale factor = 1.0. To account for experimental conditions, a temperature of 323.15 K and concentrations of 14.1156 mol L^{-1} (for neat PO), 4.787 mol L^{-1} (for CO_2 dissolved in PO at 50 bar and 313.15 K)[15] and 0.003 mol L^{-1} (for any metal complex species) were also applied.

All intermediates and transition states were characterised by normal coordinate analysis revealing either precisely zero or one imaginary frequency, respectively. In the case of transition states, the imaginary frequency corresponds to the mode of the intended reaction step. Selected intermediates were shown to be linked via the associated calculated transition state using intrinsic reaction coordinate (IRC) calculations.

An activation strain analysis (ASA) was performed using input files from the AutoDIAS python tool[16] for the PO ring-opening step (rate-determining step)[8] using compound $\mathbf{2_{Cl}}$ (reaction sequence: **I** to $\mathbf{TS_{I\text{-}II}}$ to **II**). The complex was fragmented at each M–R bond (fragment1 = PO and methyl carbonate moieties (anion); fragment2 = macrocyclic ligand and both coordinated metals (cation); Figure S52). Energy was plotted as a function of bond distance for the i) breaking C–O bond in PO; and b) forming O–C bond between PO and methyl carbonate. The transition state is noted by dashed lines in Figure S52. The total (E_{tot}), interaction (E_{int}), and distortion/strain (E_{strain}) energies (in kcal mol^{-1}) across the entire reaction coordinate are considered.

Full coordinates for all the calculated stationary points are included as part of the supplementary information (.xyz). Full coordinates for all structures, together with computed energies and vibrational frequency data, are available via the corresponding Gaussian16 output files and calculation spreadsheet, stored in the open-access digital repository, https://figshare.com/s/cb3b36a0663a579f2d0e (note this link is currently private but will be updated to a public DOI before publication).

Modelling Propagation

Key intermediates and transition states during propagation were calculated and analysed. The propagation sequence for the ROCOP of *R*-PO and carbon dioxide was previously modelled and analysed in detail for a similar catalyst that differs only in the identity of the bridging moiety between the phenoxide groups (ethyl), discussed here as complex **1**.[8] The new catalysts ($\mathbf{2_R}$) are assumed to proceed via the same mechanism, where the rate determining step involves PO coordination and activation at Co(III) (Figure S44). The starting point of the calculations are structures ***0_phenR*** (R = H, F, Cl, OMe, Me) which all adopt bowl-shape conformations and the acetate ligand of the pre-catalyst complexes ($\mathbf{2_R}$) is replaced by one methyl carbonate ligand, modelling a growing polymer chain.

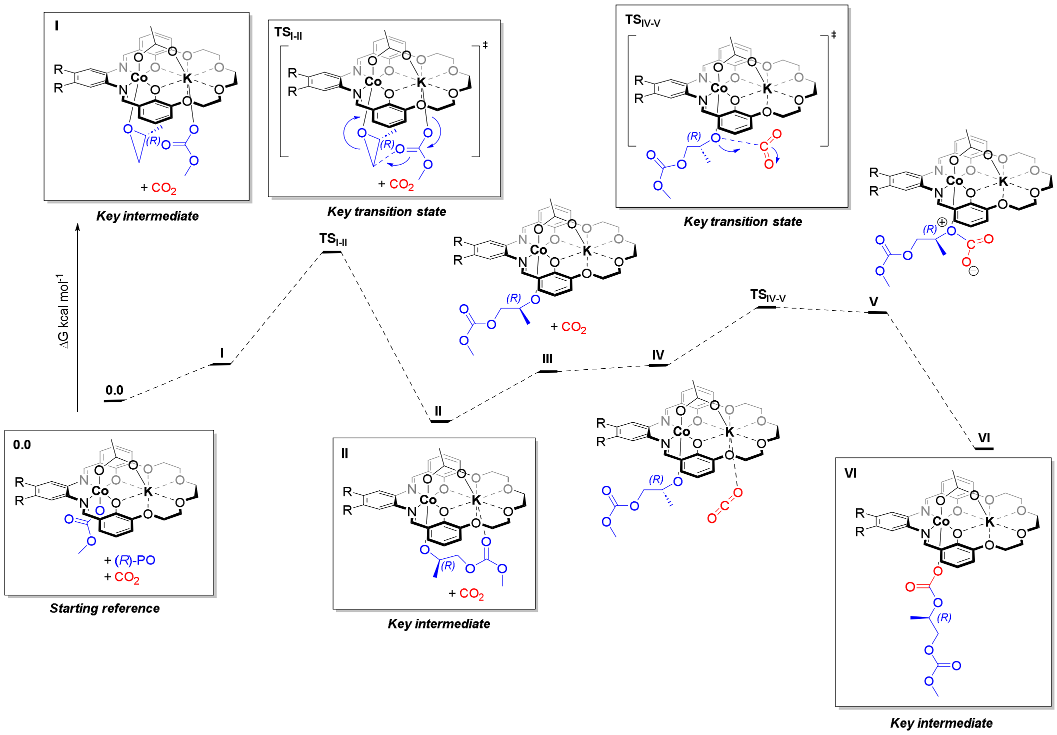

Figure S44 Previously calculated lowest energy pathway for the propagation reactions in ROCOP of *R*-PO and carbon dioxide. Key intermediates and transition states that are analysed are highlighted.

Table S4 Computed Gibbs free energies of key intermediates and transition states in the propagation reactions occurring during the ROCOP of *R*-PO and carbon dioxide using **2**$_{\mathbf{H}}$.

Structure	BS1		BS2			
	G (Hartree)[a]	ΔΔG (kcal mol^{-1})[a]	G (Hartree)[b]	ΔΔG (kcal mol^{-1})[b]	G (Hartree)[c]	ΔΔG (kcal mol^{-1})[c]
***R*-PO**	-193.025528		-193.082349		-193.078971	
**CO_2**	-188.582760		-188.626873		-188.624676	
**0_phenH**	-2843.428084		-4081.027449		-4081.029665	
**Reference (R-PO+0_phenH)**	-3036.453612	0.00	-4274.109798	0.00	-4274.108636	0.00
**Reference (*R*-PO+0_phenH+CO_2)**	-3225.036372	0.00	-4462.736671	0.00	-4462.733312	0.00
**I**	-3036.440194	8.42	-4274.091573	11.44	-4274.093466	9.52
**TS$_{I\text{-}II}$**	-3036.414599	24.48	-4274.065645	27.71	-4274.069134	24.79
**II**	-3036.456448	-1.78	-4274.108240	0.98	-4274.110738	-1.32
**TS$_{IV\text{-}V}$**	-3225.019372	10.67	-4462.710419	16.47	-4462.711356	13.78
**VI**	-3225.039994	-2.27	-4462.733990	1.68	-4462.732991	0.20

[a]Calculations performed using ωB97X-D, 6-31+g(d,p)/6-311+g(d)/SDD basis set and ECP, and modified cpcm solvation model (solvent=THF, ε=16, n^2=1.867). [b]Single point calculations using ωB97X-D and def2-QZVPP and adjusted using BS1 thermal corrections. [c]*goodvibes* free energy correction applied at 323.15 K and 14.1156 M (*R*-PO), 4.787 M (CO_2) and 0.003 M (metal complexes) to BS2 SCF energy.

Table S5 Computed Gibbs free energies of key intermediates and transition states in the propagation reactions occurring during the ROCOP of *R*-PO and carbon dioxide using $\mathbf{2_F}$.

Structure	BS1		BS2			
	G (Hartree)[a]	ΔΔG (kcal mol^{-1})[a]	G (Hartree)[b]	ΔΔG (kcal mol^{-1})[b]	G (Hartree)[c]	ΔΔG (kcal mol^{-1})[c]
***R*-PO**	-193.025528		-193.082349		-193.078971	
**CO_2**	-188.582760		-188.626873		-188.624676	
**0_phenF**	-3041.921843		-4279.557570		-4279.559633	
**Reference (R-PO+0_phenF)**	-3234.947371	0.00	-4472.639919	0.00	-4472.638604	0.00
**Reference (*R*-PO+0_phenF+CO_2)**	-3423.530131	0.00	-4661.266792	0.00	-4661.263280	0.00
**I**	-3234.934193	8.27	-4472.622043	11.22	-4472.623390	9.55
**TS_{I-II}**	-3234.911878	22.27	-4472.599327	25.47	-4472.601119	23.52
**II**	-3234.951480	-2.58	-4472.639638	0.18	-4472.641564	-1.86
**TS_{IV-V}**	-3423.511235	11.86	-4661.238388	17.82	-4661.239942	14.64
**VI**	-3423.534287	-2.61	-4661.264609	1.37	-4661.263175	0.07

[a]Calculations performed using ωB97X-D, 6-31+g(d,p)/6-311+g(d)/SDD basis set and ECP, and modified cpcm solvation model (solvent=THF, ε=16, n^2=1.867). [b]Single point calculations using ωB97X-D and def2-QZVPP and adjusted using BS1 thermal corrections. [c]*goodvibes* free energy correction applied at 323.15 K and 14.1156 M (*R*-PO), 4.787 M (CO_2) and 0.003 M (metal complexes) to BS2 SCF energy.

Table S6 Computed Gibbs free energies of key intermediates and transition states in the propagation reactions occurring during the ROCOP of *R*-PO and carbon dioxide using $\mathbf{2_{Cl}}$.

Structure	BS1		BS2			
	G (Hartree)[a]	ΔΔG (kcal mol^{-1})[a]	G (Hartree)[b]	ΔΔG (kcal mol^{-1})[b]	G (Hartree)[c]	ΔΔG (kcal mol^{-1})[c]
***R*-PO**	-193.025528		-193.082349		-193.078971	
**CO_2**	-188.582760		-188.626873		-188.624676	
**0_phenCl**	-3762.659519		-5000.293162		-5000.294866	
**Reference (R-PO+0_phenCl)**	-3955.685047	0.00	-5193.375511	0.00	-5193.373837	0.00
**Reference (*R*-PO+0_phenCl+CO_2)**	-4144.267807	0.00	-5382.002384	0.00	-5381.998513	0.00
**I**	-3955.671687	8.38	-5193.357356	11.39	-5193.358372	9.70
**TS_{I-II}**	-3955.649207	22.49	-5193.332176	27.19	-5193.336109	23.67
**II**	-3955.689640	-2.88	-5193.375785	-0.17	-5193.377068	-2.03
**TS_{IV-V}**	-4144.250143	11.08	-5381.974516	17.49	-5381.975515	14.43
**VI**	-4144.271029	-2.02	-5381.999127	2.04	-5381.997740	0.49

[a]Calculations performed using ωB97X-D, 6-31+g(d,p)/6-311+g(d)/SDD basis set and ECP, and modified cpcm solvation model (solvent=THF, ε=16, n^2=1.867). [b]Single point calculations using ωB97X-D and def2-QZVPP and adjusted using BS1 thermal corrections. [c]*goodvibes* free energy correction applied at 323.15 K and 14.1156 M (*R*-PO), 4.787 M (CO_2) and 0.003 M (metal complexes) to BS2 SCF energy.

Table S7 Computed Gibbs free energies of key intermediates and transition states in the propagation reactions occurring during the ROCOP of *R*-PO and carbon dioxide using $\mathbf{2_{OMe}}$.

Structure	BS1		BS2			
	G (Hartree)[a]	ΔΔG (kcal mol^{-1})[a]	G (Hartree)[b]	ΔΔG (kcal mol^{-1})[b]	G (Hartree)[c]	ΔΔG (kcal mol^{-1})[c]
***R*-PO**	-193.025528		-193.082349		-193.078971	
**CO_2**	-188.582760		-188.626873		-188.624676	
**0_phenOMe**	-3072.393273		-4310.047461		-4310.048915	
**Reference (R-PO+0_phenOMe)**	-3265.418801	0.00	-4503.129810	0.00	-4503.127886	0.00
**Reference (*R*-PO+0_phenOMe+CO_2)**	-3454.001561	0.00	-4691.756683	0.00	-4691.752562	0.00
**I**	-3265.405819	8.15	-4503.112031	11.16	-4503.113221	9.20
**TS_{I-II}**	-3265.378493	25.29	-4503.084068	28.70	-4503.087555	25.31
**II**	-3265.421274	-1.55	-4503.127936	1.18	-4503.129932	-1.28
**TS_{IV-V}**	-3453.984813	10.51	-4691.730410	16.49	-4691.731028	13.51
**VI**	-3454.001382	0.11	-4691.749472	4.52	-4691.748330	2.66

[a]Calculations performed using ωB97X-D, 6-31+g(d,p)/6-311+g(d)/SDD basis set and ECP, and modified cpcm solvation model (solvent=THF, ε=16, n^2=1.867). [b]Single point calculations using ωB97X-D and def2-QZVPP and adjusted using BS1 thermal corrections. [c]*goodvibes* free energy correction applied at 323.15 K and 14.1156 M (*R*-PO), 4.787 M (CO_2) and 0.003 M (metal complexes) to BS2 SCF energy.

Table S8 Computed Gibbs free energies of key intermediates and transition states in the propagation reactions occurring during the ROCOP of *R*-PO and carbon dioxide using using $\mathbf{2_{Me}}$.

Structure	BS1		BS2			
	G (Hartree)[a]	ΔΔG (kcal mol^{-1})[a]	G (Hartree)[b]	ΔΔG (kcal mol^{-1})[b]	G (Hartree)[c]	ΔΔG (kcal mol^{-1})[c]
***R*-PO**	-193.025528		-193.082349		-193.078971	
**CO_2**	-188.582760		-188.626873		-188.624676	
**0_phenMe**	-2921.996613		-4159.623075		-4159.624978	
**Reference (R-PO+0_phenMe)**	-3115.022141	0.00	-4352.705424	0.00	-4352.703949	0.00
**Reference (*R*-PO+0_phenMe+CO_2)**	-3303.604901	0.00	-4541.332297	0.00	-4541.328625	0.00
**I**	-3115.008578	8.51	-4352.687047	11.53	-4352.688840	9.48
**TS_{I-II}**	-3114.982839	24.66	-4352.661042	27.85	-4352.664350	24.85
**II**	-3115.023970	-1.15	-4352.702914	1.58	-4352.705490	-0.97
**TS_{IV-V}**	-3303.587718	10.78	-4541.305600	16.75	-4541.307239	13.42
**VI**	-3303.606651	-1.10	-4541.327360	3.10	-4541.326967	1.04

[a]Calculations performed using ωB97X-D, 6-31+g(d,p)/6-311+g(d)/SDD basis set and ECP, and modified cpcm solvation model (solvent=THF, ε=16, n^2=1.867). [b]Single point calculations using ωB97X-D and def2-QZVPP and adjusted using BS1 thermal corrections. [c]*goodvibes* free energy correction applied at 323.15 K and 14.1156 M (*R*-PO), 4.787 M (CO_2) and 0.003 M (metal complexes) to BS2 SCF energy.

Comparison of catalyst activity against key structural, kinetic and thermodynamic data

The experimentally determined catalyst activity is compared against key structural (Co–O bond length and Wiberg Bond Index (WBI); Figures S45 and S46), kinetic (PO ring-opening (**TS_{I-II}**) and CO_2 insertion (**TS_{IV-V}**); Figures S47 and S48) and thermodynamic (alkoxide **II**, carbonate **VI** and difference (**VI–II**); Figures S49 and S50) data. In general, the differences for all structural parameters were very small (Table S9), approaching the accuracy limit for the DFT methods employed. For instance, there was no detectable difference in NPA charge on Co (1.33 (BS1), 1.19 (BS2)) or K (0.93 (BS1), 0.93 (BS2)) for any complex in the $\mathbf{2_R}$ series. The small calculated differences suggest different trends may be observed if other functionals were employed, and additionally do not reproduce the kinetic trends observed experimentally. For instance, comparing the most

extreme cases determined experimentally: the difference (1.11 kcal mol^{-1}) between **ΔΔGTS$_{I-II}$** for **2$_H$** (24.79 kcal mol^{-1}) and **2$_{Cl}$** (23.67 kcal mol^{-1}) can be estimated to give a rate difference of x5.7. Although this is similar to the observed difference in rate (x6.3), the calculations predict that **2$_{Cl}$** should be faster than **2$_H$**, inverting the observed trend. In general, the calculations encompass the relative rates of reaction observed (experimentally **2$_H$** (fastest) = 6.3 x **2$_{Cl}$** (slowest); computationally **2$_F$** (fastest) = 16.1 x **2$_{OMe}$** (slowest)), but fail to predict the trends i.e. the two slowest catalysts (**2$_F$** and **2$_{Cl}$**) are predicted to be considerably faster than others.

Table S9 Comparison of calculated ranges for various parameters to differentiate catalysts **2$_R$**

Parameter	Largest calculated difference between 2$_R$
Co–O bond length for coordinated PO in ***0_phenR*** (Å)	0.00149
Co–O WBI for coordinated PO (a.u.)	0.0024
NPA charge on Co (a.u.)	0.00
**TS$_{I-II}$** ΔG (kcal mol^{-1})	1.79
**TS$_{IV-V}$** ΔG (kcal mol^{-1})	2.12
**II** ΔG (kcal mol^{-1})	1.06
**VI** ΔG (kcal mol^{-1})	2.59
Energy difference between **II** and **VI** ΔΔG (kcal mol^{-1})	2.42

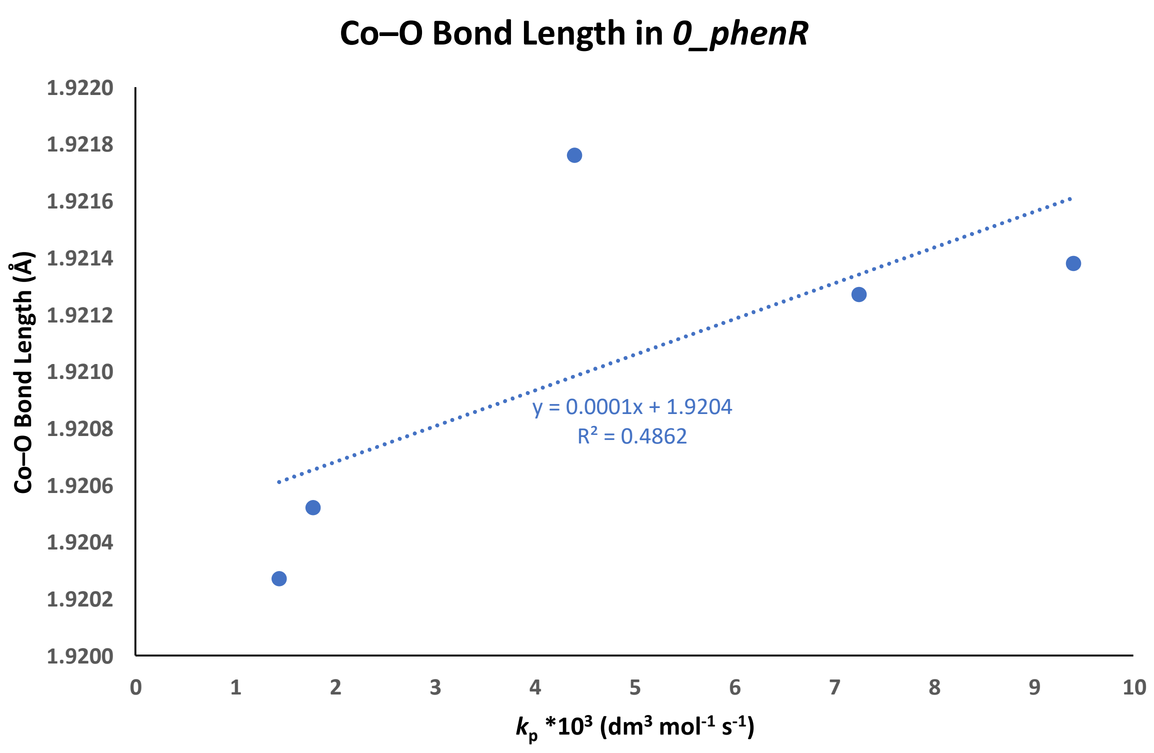

Figure S45 Comparison of Co–O bond length in ***0_phenR*** against propagation rate coefficient.

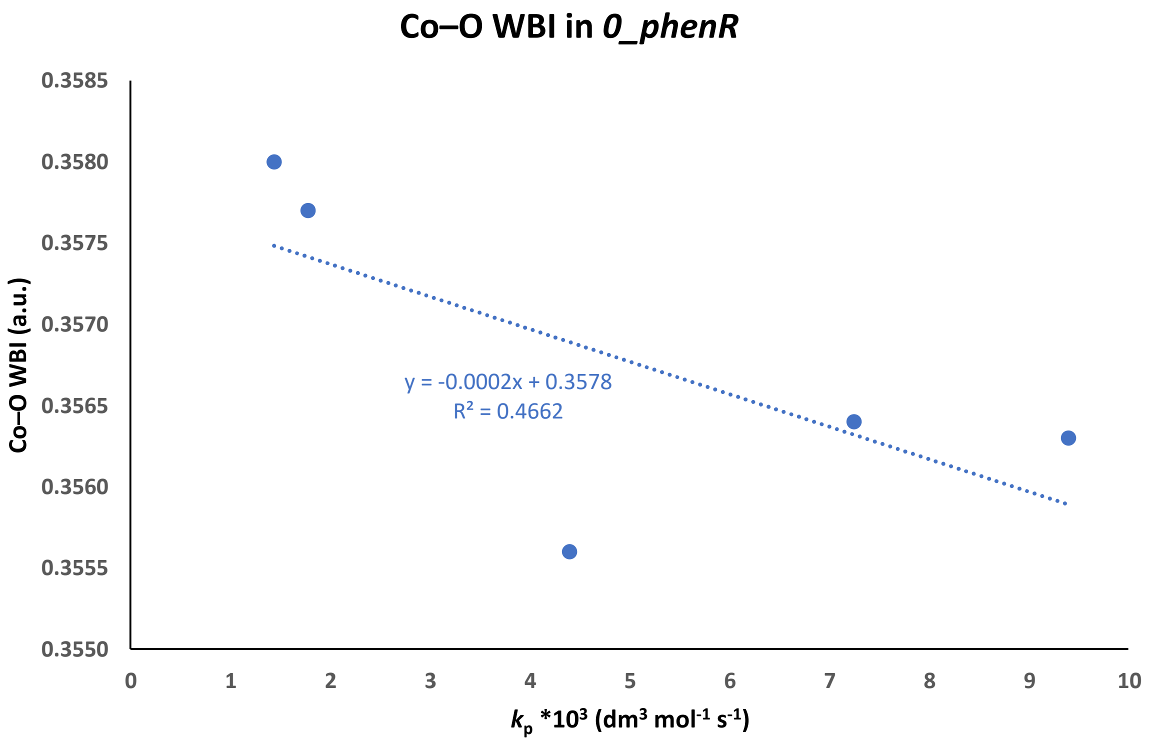

Figure S46 Comparison of Co–O WBIs in ***0_phenR*** against propagation rate coefficient.

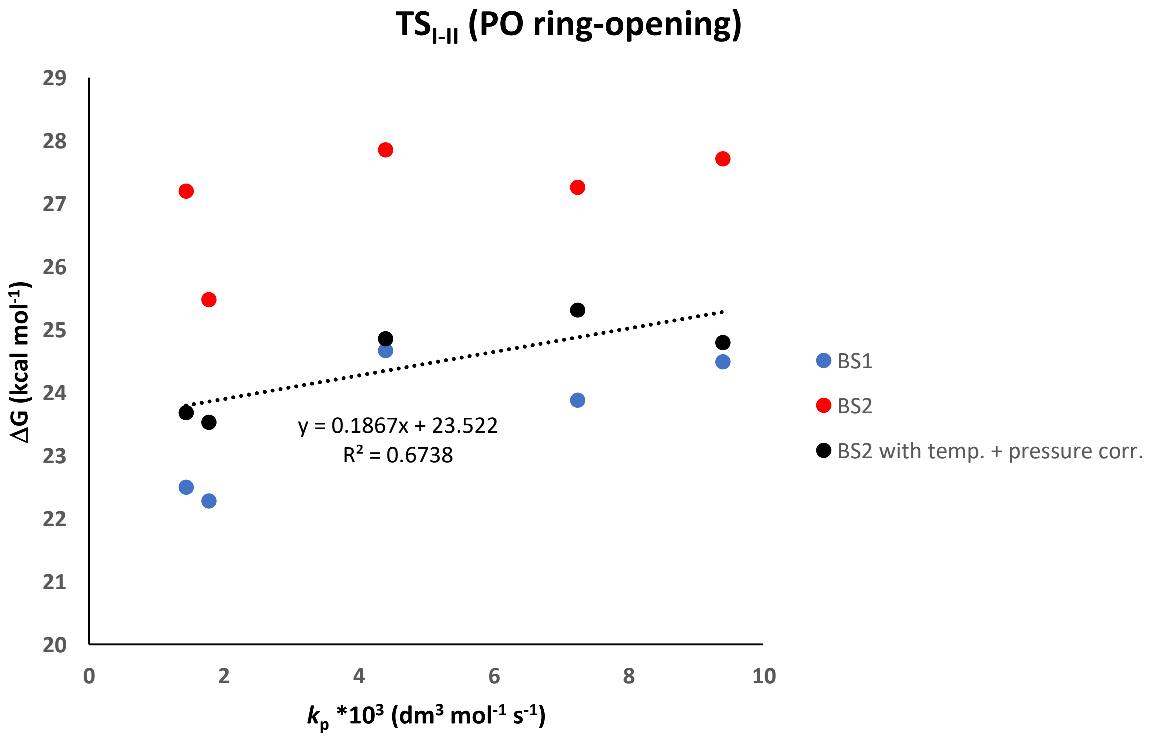

Figure S47 Comparison of PO ring-opening transition state barrier (**TS_{I-II}**) against propagation rate coefficient.

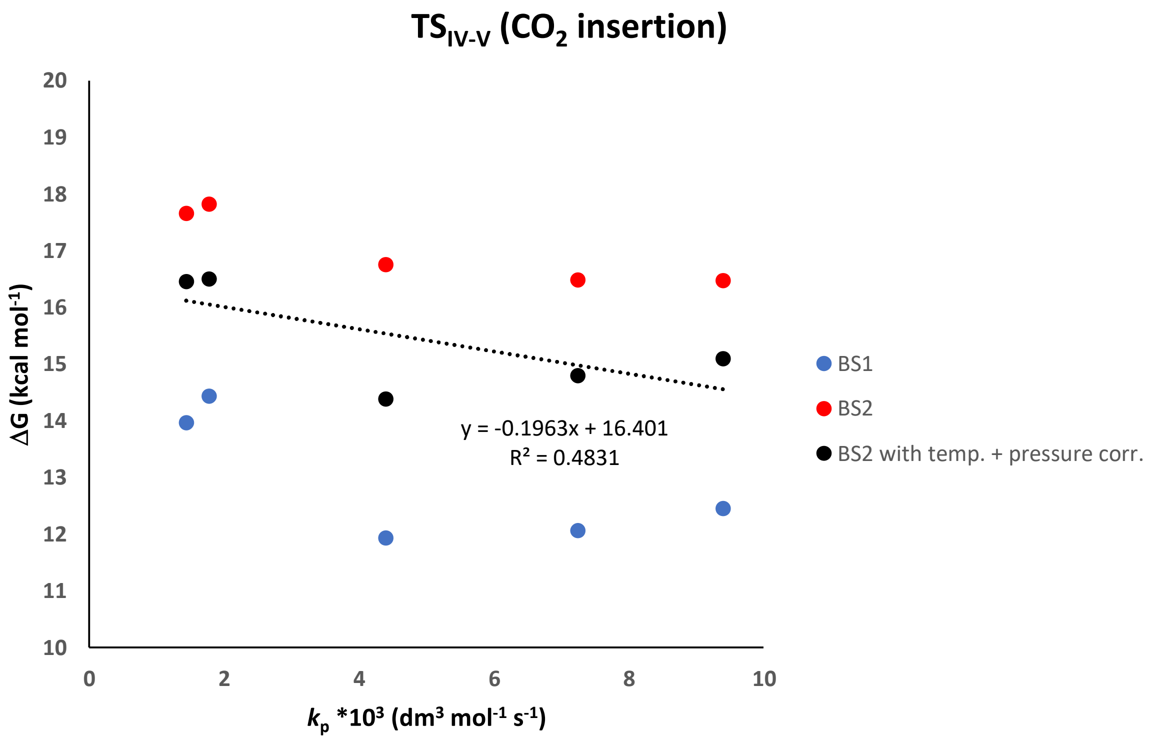

Figure S48 Comparison of CO_2 insertion transition state barrier (**TS$_{IV-V}$**) against propagation rate coefficient.

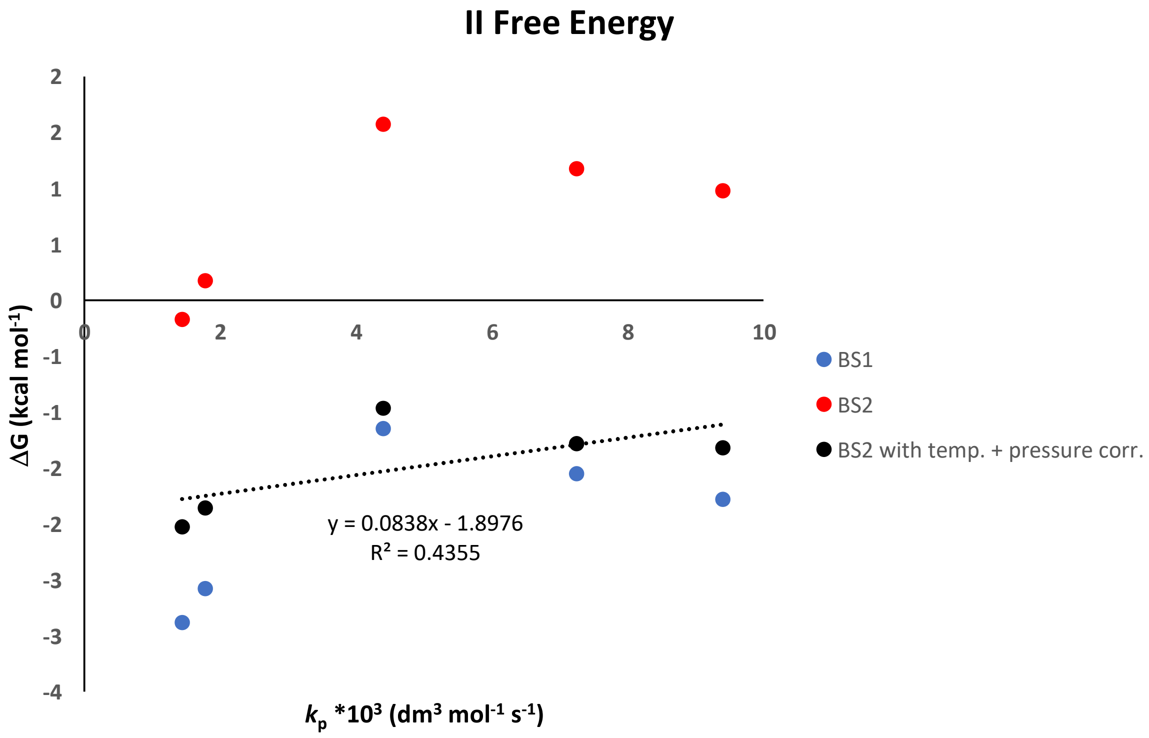

Figure S49 Comparison of free energy for **II** against propagation rate coefficient.

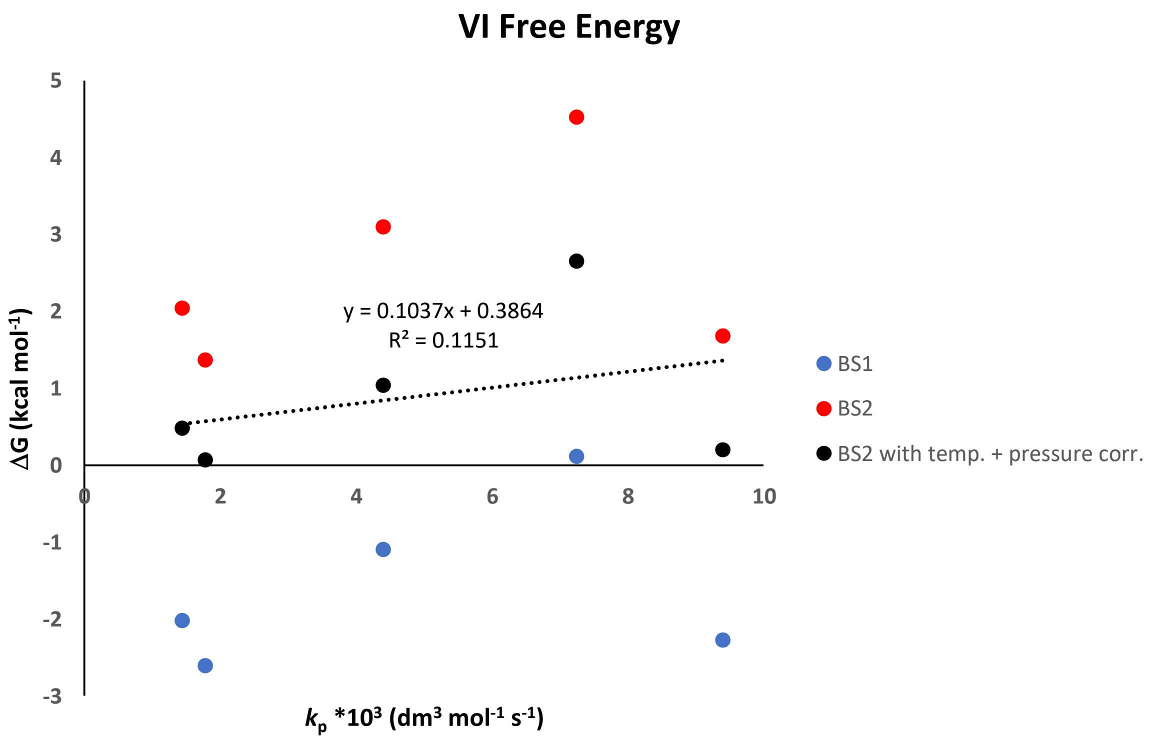

Figure S50 Comparison of free energy for **VI** against propagation rate coefficient.

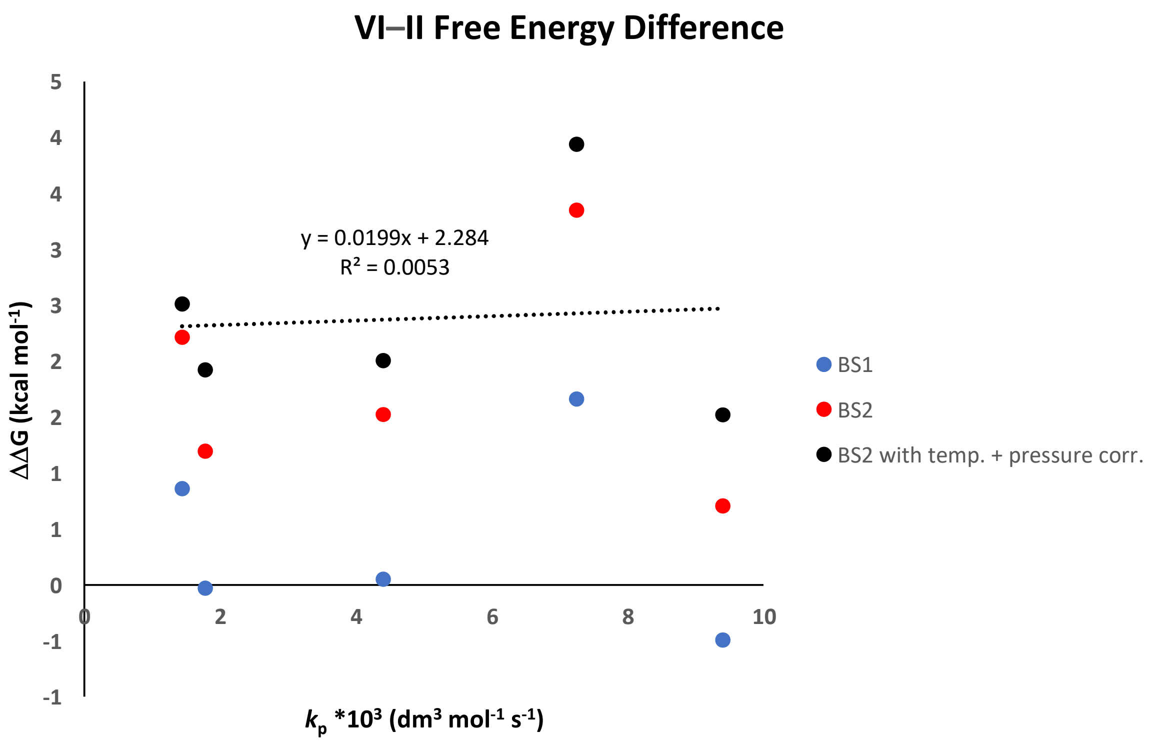

Figure S51 Comparison of free energy difference between **II** and **VI** against propagation rate coefficient.

Activation Strain Analysis

Relief of ring strain is clearly an important thermodynamic driving force for the activation of 3-membered cyclic monomers such as PO. As the rate-determining step in the copolymerisation of PO and carbon dioxide specifically involves PO ring-opening, it is interesting to consider the relative proportion that relief of ring strain contributes to the activation energy required to surmount the transition state geometry. In order to address this question, Activation Strain Analysis (ASA) was performed.[8,16-18] This approach interrogates energy changes on the potential energy surface (PES) around transition states. The simplest analysis involves deconvoluting the total energy of the system into the (stabilising) interaction energy $\Delta E_{int}(\zeta)$ between two fragments and the (destabilising) strain energy $\Delta E_{strain}(\zeta)$ required to distort these fragments along the reaction coordinate. Although ASA is typically applied to bimolecular reactions (making fragment choice straightforward), there are examples of its use for intramolecular processes.[19] In these cases, fragment choice is critical for obtaining meaningful information. Here, the interaction of the polymer chain (methyl carbonate) and PO monomer fragment ($[C_3H_6O+C_2H_3O_3]^-$) with the macrocyclic metal fragment ($[LCoK]^+$) was considered (Figure S51). Hence, our ASA calculations should provide insight into the strain required to distort the growing polymer chain moieties (polymer and monomer) and bimetallic catalyst fragments to the TS geometry, and the difference in interaction energies (i.e. the binding energy) between the catalyst and both moieties of the growing polymer as the TS is approached.

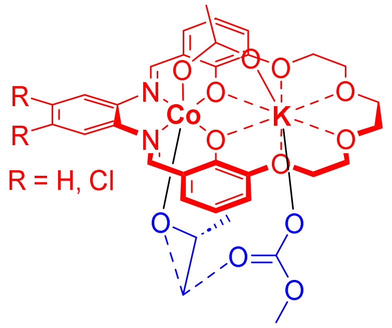

Figure S52 Fragmentation used during Activation Strain Analysis. Blue = fragment1, red = fragment2, black bonds represent the fragmentation points and the dotted blue lines represent the breaking and forming bonds during the transition state.

Activation Strain Analysis on the potential energy surface associated with the rate-determining ring-opening of PO for both $\mathbf{2_H}$ and $\mathbf{2_{Cl}}$ suggests that the interaction and distortion components for both catalysts are very similar. This analysis aligns well with the similar TS energies calculated for both catalysts, supporting the conclusion that altering the macrocyclic ligand backbone at the peripheral phenyl group does not have a large impact on the (calculated) TS.

Plotting the total energy (E_{tot}, black line), interaction energy (E_{int}, red line), strain energy for fragment 1 ($E_{strain,1}$, dark blue) and fragment 2 ($E_{strain,2}$, light blue) against the bond distance for either the breaking bond (Figures S53 ($\mathbf{2_H}$) and S55 ($\mathbf{2_{Cl}}$)) or the newly forming bond (Figures S54 ($\mathbf{2_H}$) and S56 ($\mathbf{2_{Cl}}$)) allows visualisation of the different contributions to the transition state energy. As the fragments traverse the potential energy surface from their initial geometry (**I**) over the transition state geometry ($\mathbf{TS_{I\text{-}II}}$) and end at the final geometry (**II**), E_{int} steadily decreases: $\Delta E_{int}(\mathbf{TS_{I\text{-}II}}\text{–}\mathbf{I})$ = -11.4 ($\mathbf{2_H}$) and -11.6 ($\mathbf{2_{Cl}}$) kcal mol^{-1} and $\Delta E_{int}(\mathbf{II}\text{–}\mathbf{TS_{I\text{-}II}})$ = -14.5 ($\mathbf{2_H}$) and -14.8 ($\mathbf{2_{Cl}}$) kcal mol^{-1}, suggesting the growing polymer increasingly interacts more strongly with the bimetallic catalyst during PO ring-opening and insertion. Overall, the ring-opened intermediate (**II**) interacts more strongly with the catalyst than the PO-coordinated intermediate (**I**) by -26.0 ($\mathbf{2_H}$) and -26.5 ($\mathbf{2_{Cl}}$) kcal mol^{-1}. The incorporation of pendent chlorine groups on the ligand appears to stabilise the growing polymer chain slightly more than the protio analogue.

The strain energy for the bimetallic fragment ($E_{strain,2}$) remains almost constant ($\Delta E_{strain,2}(\mathbf{II}\text{–}\mathbf{I})$ = +4.3 ($\mathbf{2_H}$) and +4.2 ($\mathbf{2_{Cl}}$) kcal mol^{-1}) over the reaction coordinate and sits around 0 kcal mol^{-1}. Intuitively this is reasonable as the macrocyclic ligand is likely to have almost no associated ring strain and hardly changes geometry over the course of the reaction step. Bond lengths and angles within this fragment remain almost constant at each geometry (**I**, $\mathbf{TS_{I\text{-}II}}$ and **II**). On the other hand, the strain energy associated with distorting the [PO + methyl carbonate] fragment increases to a maximum just after the transition state geometry ($\Delta E_{strain,1}$(max) = +25.2 ($\mathbf{2_H}$) and +25.3 ($\mathbf{2_{Cl}}$) kcal mol^{-1}, $\Delta E_{strain,1}(\mathbf{TS_{I\text{-}II}}\text{–}\mathbf{I})$ = +23.2 ($\mathbf{2_H}$ and $\mathbf{2_{Cl}}$) kcal mol^{-1}), before relaxing again as the geometry for **II** is approached. The overall strain energy increases over the reaction coordinate ($\Delta E_{strain,1}(\mathbf{II}\text{–}\mathbf{I})$ = +8.4 kcal mol^{-1} ($\mathbf{2_H}$ and $\mathbf{2_{Cl}}$)). These results suggest that different substituents on the macrocyclic ligand do not alter the strain barrier required to ring open PO when coordinated.

Taken together this suggests the energy associated with the $\mathbf{TS_{I\text{-}II}}$ transition state barrier is associated with overcoming the required strain energy to distort the growing polymer chain to the TS geometry, and this geometry is consistently strained

across ligand variations. Any benefit from the stronger stabilising interaction of the growing polymer chain with the bimetallic catalyst imparted from altering the substituents on the ligand is marginal compared with the strain energy. It is reasonable to conclude that, at least this computational model, will calculate rate determining transition state barrier heights based primarily on variations in polymer chain geometry rather than electronic contributions from varying the ligand backbone. Although modelling this polymerisation system appears to approach the accuracy limit for standard DFT methods, perhaps more advanced, but time consuming, computational methods (e.g. Complete Active Space Self Consistent Field (CASSCF) calculations) might be more insightful.

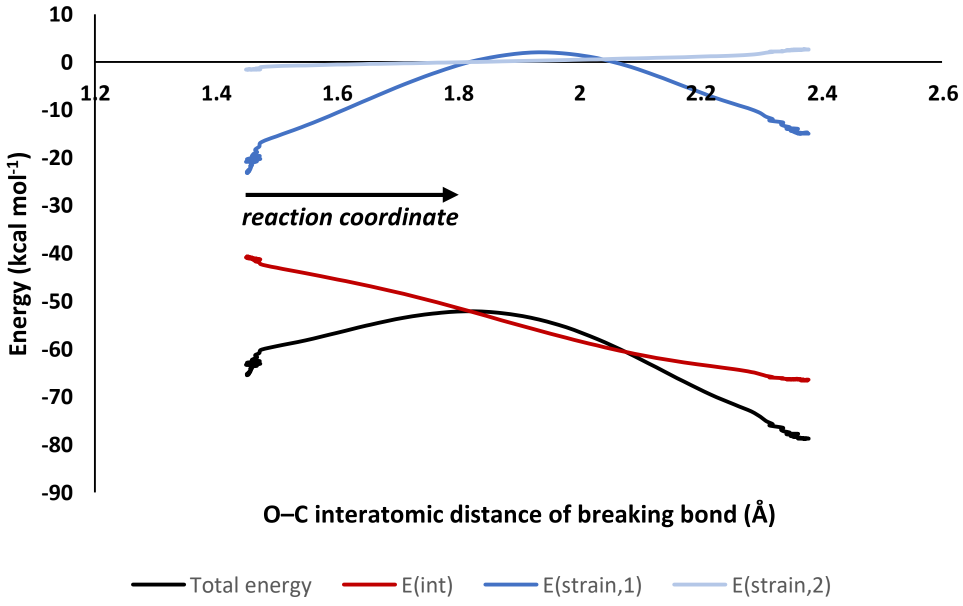

Figure S53 Activation Strain Analysis during approach of $\mathbf{TS_{I\text{-}II}}$ for $\mathbf{2_H}$ plotted as a function of the breaking bond.

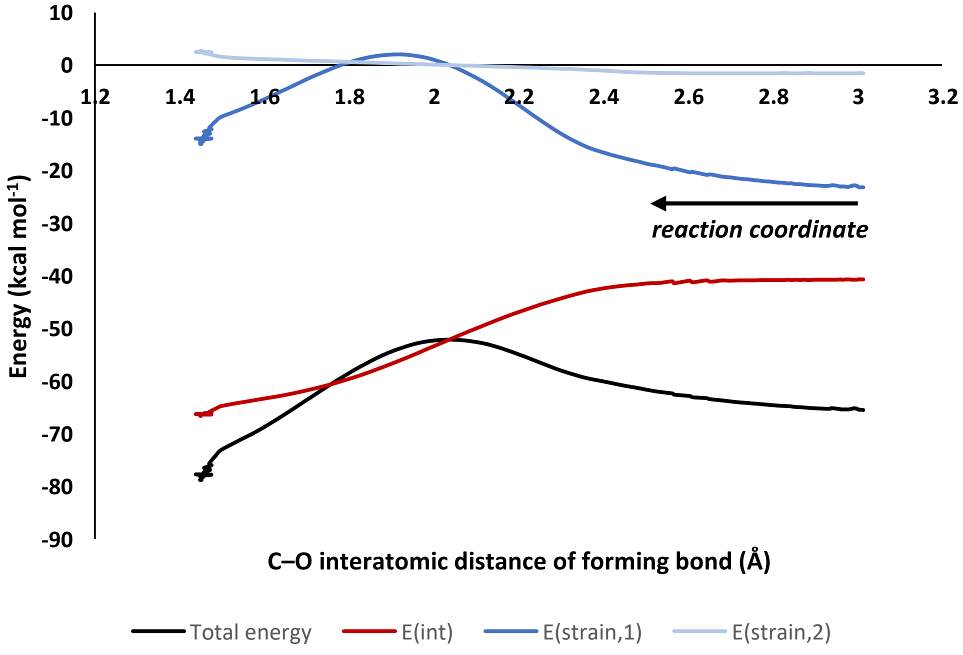

Figure S54 Activation Strain Analysis during approach of $\mathbf{TS_{I\text{-}II}}$ for $\mathbf{2_H}$ plotted as a function of the forming bond.

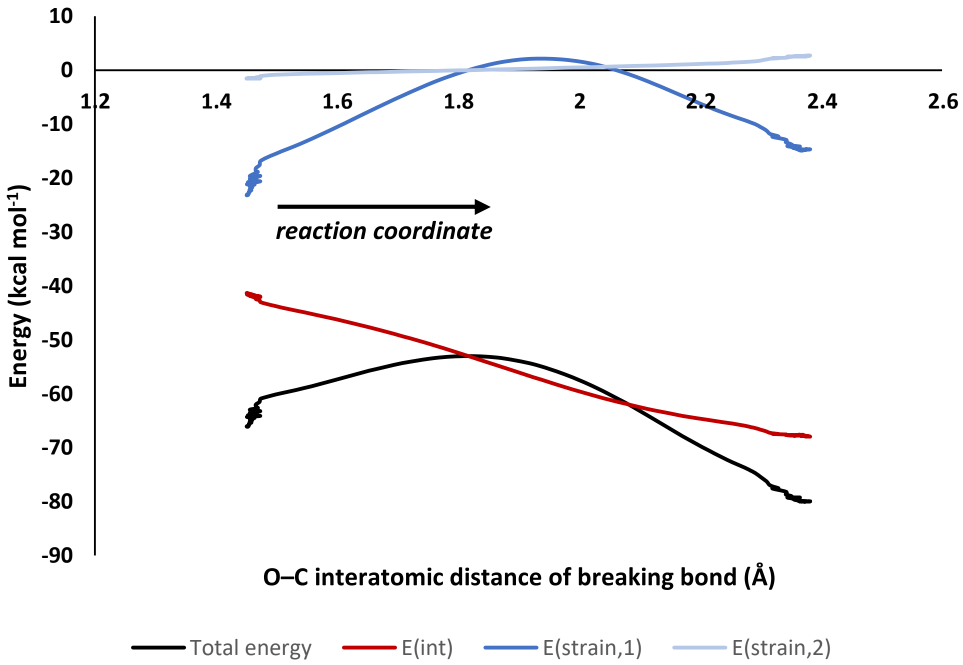

Figure S55 Activation Strain Analysis during approach of $\mathbf{TS_{I\text{-}II}}$ for $\mathbf{2_{Cl}}$ plotted as a function of the breaking bond.

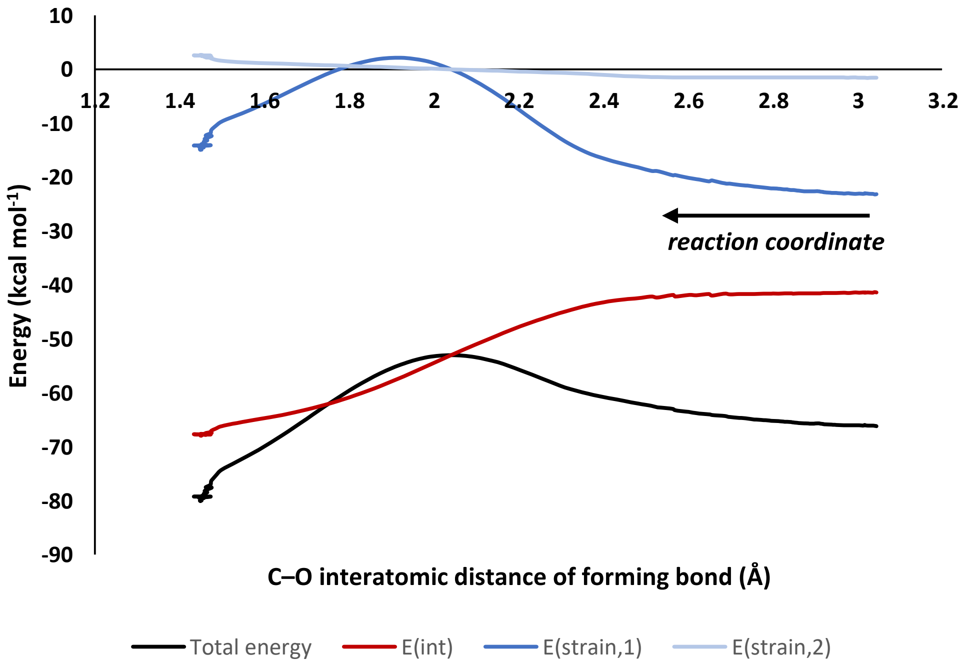

Figure S56 Activation Strain Analysis during approach of $\mathbf{TS_{I\text{-}II}}$ for $\mathbf{2_{Cl}}$ plotted as a function of the forming bond.

Computational coordinates

1_phen_bowl.log

SCF (wB97x) = SCF
E(SCF)+ZPE(0 K)= -2843.349580
H(298 K)= -2843.307155
G(298 K)= -2843.424572
Lowest Frequency = 19.6697cm-1

Co	2.871279	11.755593	10.729421
K	2.963717	8.226498	10.641220
O	1.719842	11.724004	12.253131
O	1.834126	10.428293	9.839561
O	0.626795	8.219833	9.163566
N	1.781379	13.170050	10.027052
O	1.262406	5.841264	10.547925
O	1.500865	9.513221	12.642909
C	0.616007	10.554745	9.446926
C	0.577828	13.033912	9.579492
H	0.002823	13.933271	9.356195
C	-0.079002	11.788560	9.331699
C	2.395857	14.432780	10.181925
C	-0.100039	9.366445	9.065323
C	-1.428381	11.824066	8.891658
H	-1.929229	12.785313	8.816281
C	2.001470	15.607452	9.541682
H	1.171640	15.608826	8.843546
C	1.271049	10.696516	12.892003
C	-2.088917	10.668160	8.563410
H	-3.121054	10.690539	8.232077
C	2.704513	16.786016	9.774263
C	-0.022472	6.985974	8.905558
H	-0.351471	6.938888	7.859412
H	-0.895649	6.876660	9.561196
C	-1.411852	9.429516	8.646817
H	-1.944647	8.526081	8.372756
C	0.379967	11.083865	14.063433
H	-0.030254	10.194492	14.543297
H	-0.434401	11.726490	13.717891
H	0.962673	11.654823	14.792497
C	1.948262	4.686431	10.999236
H	1.620120	3.800480	10.439157
H	1.667600	4.553858	12.047824
O	4.038785	10.450460	11.473334
O	5.103196	8.267690	12.395163
N	3.827761	13.178352	11.589241
O	3.884774	5.811449	11.800002
C	4.724425	10.578534	12.553479
C	4.554199	13.050165	12.649711
H	4.897513	13.953564	13.155254
C	4.972911	11.807855	13.219823
C	3.509704	14.437133	11.033637
C	5.319043	9.397650	13.120315
C	5.724092	11.840646	14.424433
H	5.892138	12.797597	14.910949
C	4.225661	15.615349	11.243739
H	5.116318	15.620725	11.862391
C	6.232238	10.686895	14.964409
H	6.797830	10.706614	15.889260
C	3.812252	16.790210	10.622503
C	5.619354	7.037732	12.874473
H	5.173212	6.794859	13.847394
H	6.709707	7.095954	12.985464
C	6.032554	9.455150	14.297699
H	6.454561	8.553737	14.728041
C	3.458627	4.801781	10.906243
H	3.904426	3.834033	11.180167
H	3.777423	5.040455	9.879592
O	4.020556	11.960315	9.199848
O	4.433899	9.796940	8.657160
C	4.535469	11.009962	8.519044
C	0.952340	5.861616	9.168252
H	1.856491	5.991481	8.555881
H	0.472905	4.919181	8.868598
C	5.286927	5.964390	11.864334
H	5.763928	5.025735	12.180852
H	5.689520	6.238283	10.877571
O	5.295786	11.527529	7.507850
C	5.938449	10.589295	6.654417
H	6.485983	11.182921	5.921851
H	6.633587	9.957942	7.214869
H	5.208044	9.953579	6.146172
H	4.373555	17.704906	10.780491
H	2.401426	17.697442	9.270151

1_phenCl.log

SCF (wB97x) = SCF
E(SCF)+ZPE(0 K)= -3762.578890
H(298 K)= -3762.533550
G(298 K)= -3762.659519
Lowest Frequency = 14.4022cm-1

Co	2.443124	6.405992	23.104670
K	5.701149	7.589704	21.835370
O	1.372433	6.370684	21.510989
O	3.180258	8.089234	22.663869
O	3.853223	5.627331	22.086039
O	4.566582	10.121523	21.867494
O	3.399070	6.239802	24.760904
O	6.000736	5.051597	20.743349
O	8.043970	6.976125	20.377621
O	6.997807	9.700205	20.513515
O	5.486443	6.995870	24.384523
O	0.369769	8.399727	21.535511
N	1.657833	4.715399	23.547596
N	1.036377	7.144842	24.154932
C	4.200054	4.388389	22.098938
C	5.391213	4.015817	21.381858
C	2.939388	9.193630	23.277747
C	3.707561	10.348242	22.898308
C	0.627721	7.304769	21.054857
C	1.040097	8.343123	24.639052
H	0.270103	8.614349	25.361696
C	3.978259	2.008496	22.723320
H	3.414360	1.234811	23.236850
C	4.604146	6.523314	25.104182
C	1.824616	10.629928	24.946036
H	1.074627	10.733873	25.725258
C	5.841506	2.714051	21.373434
H	6.748193	2.449758	20.841182
C	3.490885	3.343701	22.754716
C	2.611965	11.695394	24.594070
H	2.510796	12.652184	25.093838
C	2.241145	3.570506	23.408400
H	1.750023	2.683530	23.809525
C	5.354484	11.196745	21.385416
H	6.062806	11.522268	22.158136
H	4.716058	12.044602	21.106417
C	1.965606	9.373475	24.297045
C	3.558005	11.551236	23.551896
H	4.164076	12.406241	23.273707
C	7.182121	4.787785	20.003682
H	6.980421	4.050733	19.215985
H	7.964246	4.398602	20.667718
C	5.133251	1.697092	22.055425
H	5.507971	0.679990	22.034184

C	7.763088	9.226240	19.422769
H	8.363892	10.042468	18.994809
H	7.099284	8.847646	18.631093
C	8.703662	8.142493	19.917069
H	9.410070	7.889528	19.114336
H	9.272655	8.523162	20.769391
C	7.642491	6.075641	19.363063
H	8.488163	5.847396	18.699208
H	6.831344	6.497853	18.753657
C	6.092414	10.723635	20.155724
H	5.375169	10.353367	19.408653
H	6.631054	11.578738	19.722555
C	4.885371	6.217468	26.568026
H	4.229649	6.822752	27.200737
H	5.926411	6.431603	26.812375
H	4.665533	5.167030	26.777632
O	0.086124	6.921676	19.861237
C	-0.782201	7.857176	19.233328
H	-0.257560	8.788634	19.003292
H	-1.112629	7.380138	18.310344
H	-1.645483	8.079810	19.866636
C	0.390366	4.868039	24.141114
C	-0.519798	3.841401	24.374744
C	0.053053	6.190495	24.469009
C	-1.752689	4.124988	24.951163
H	-0.301312	2.817738	24.096714
C	-1.192334	6.473957	25.022054
C	-2.089617	5.442663	25.274466
H	-1.493644	7.490835	25.241392
Cl	-2.862825	2.815546	25.224114
Cl	-3.636032	5.837125	25.962808

1_phenF.log

SCF (wB97x) = SCF
E(SCF)+ZPE(0 K)= -3041.842902
H(298 K)= -3041.798346
G(298 K)= -3041.921843
Lowest Frequency = 15.7752cm-1

Co	2.442263	6.406042	23.104815
K	5.700146	7.589362	21.836266
O	1.372028	6.370249	21.510539
O	3.179248	8.089138	22.662363
O	3.852543	5.628335	22.085309
O	4.567161	10.121387	21.866361
O	3.398582	6.240309	24.761197
O	6.000488	5.052910	20.741912
O	8.046082	6.975951	20.381189
O	7.000596	9.700235	20.516308
O	5.486351	6.995977	24.385580
O	0.369698	8.399475	21.532294
N	1.656919	4.715277	23.547644
N	1.035563	7.144710	24.155719
C	4.198412	4.388628	22.095371
C	5.389076	4.016335	21.377759
C	2.937561	9.194678	23.274876
C	3.705940	10.348862	22.895452
C	0.629500	7.304705	21.052143
C	1.036829	8.343945	24.635033
H	0.264523	8.617043	25.354581
C	3.972879	2.008017	22.712932
H	3.407607	1.233766	23.224163
C	4.603460	6.523687	25.104799
C	1.818822	10.632586	24.938102
H	1.067081	10.737330	25.715575
C	5.837746	2.713781	21.365635
H	6.744232	2.449726	20.832923
C	3.488018	3.343559	22.748121
C	2.606502	11.698360	24.586419
H	2.503646	12.655755	25.084726
C	2.238132	3.570853	23.403808
H	1.745669	2.682993	23.801465
C	5.355107	11.196599	21.384591
H	6.061980	11.523380	22.158124
H	4.716657	12.043846	21.103742
C	1.962092	9.375605	24.291671
C	3.554712	11.553126	23.546848
H	4.161086	12.407897	23.268610
C	7.182150	4.789101	20.002852
H	6.980410	4.053759	19.213544
H	7.963254	4.397698	20.666808
C	5.127763	1.696258	22.044304
H	5.500670	0.678528	22.020408
C	7.768419	9.226782	19.427172
H	8.370549	10.043100	19.001235
H	7.106461	8.849000	18.633564
C	8.707350	8.142299	19.922952
H	9.415478	7.889545	19.121649
H	9.274574	8.522099	20.776838
C	7.644704	6.077449	19.364848
H	8.490832	5.849338	18.701506
H	6.834675	6.501490	18.755221
C	6.095379	10.723088	20.156488
H	5.379604	10.352094	19.408354
H	6.634377	11.578200	19.723754
C	4.884244	6.218030	26.568867
H	4.228446	6.823504	27.201329
H	5.925242	6.432063	26.813527
H	4.664253	5.167649	26.778629
O	0.092552	6.922575	19.855870
C	-0.772579	7.858889	19.224838
H	-0.246600	8.790365	18.997928
H	-1.099047	7.382765	18.299962
H	-1.638649	8.081381	19.854395
C	0.390388	4.867019	24.145658
C	-0.514960	3.831591	24.384173
C	0.053352	6.189123	24.474645
C	-1.732504	4.129514	24.964523
H	-0.312302	2.803029	24.111462
C	-1.190210	6.478673	25.039004
C	-2.067433	5.441344	25.288567
H	-1.506192	7.489546	25.266378
F	-2.620060	3.157241	25.195552
F	-3.267411	5.692030	25.820637

1_phenMe.log

SCF (wB97x) = SCF
E(SCF)+ZPE(0 K)= -2921.915745
H(298 K)= -2921.869547
G(298 K)= -2921.996613
Lowest Frequency = 13.6038cm-1

Co	2.432372	6.445729	23.204130
K	5.678307	7.582515	21.870460
O	1.340866	6.363775	21.624560
O	3.167028	8.114133	22.699137
O	3.829708	5.637162	22.187616
O	4.552347	10.118439	21.827783
O	3.412748	6.327338	24.852786
O	5.955284	5.018416	20.826890
O	8.002058	6.922048	20.384136
O	6.965123	9.652493	20.456731
O	5.500696	7.062190	24.436860
O	0.348825	8.398239	21.578132
N	1.650942	4.770267	23.707631
N	1.042330	7.218759	24.251878
C	4.168501	4.394713	22.219482
C	5.345862	3.999303	21.493789
C	2.936058	9.239213	23.281888
C	3.702730	10.378823	22.859849

C	0.596908	7.282801	21.140831
C	1.051352	8.430281	24.692571
H	0.288105	8.725859	25.412931
C	3.935524	2.031537	22.889363
H	3.372092	1.272172	23.424649
C	4.622011	6.611465	25.175787
C	1.841141	10.727895	24.912739
H	1.098402	10.857725	25.695237
C	5.786514	2.693214	21.501525
H	6.683426	2.412116	20.961323
C	3.461089	3.369678	22.903878
C	2.626893	11.782365	24.520255
H	2.531053	12.754356	24.991160
C	2.221034	3.621183	23.577103
H	1.727819	2.745378	23.999692
C	5.334038	11.177183	21.302874
H	6.052282	11.526805	22.055798
H	4.692776	12.016631	21.005236
C	1.974211	9.452927	24.303700
C	3.562329	11.603866	23.475641
H	4.167571	12.448175	23.164830
C	7.122541	4.730336	20.075229
H	6.904470	3.976821	19.307610
H	7.913235	4.351220	20.735104
C	5.079353	1.696099	22.210770
H	5.443916	0.675006	22.203253
C	7.717064	9.146327	19.371472
H	8.315814	9.948500	18.914879
H	7.043608	8.748845	18.597419
C	8.659654	8.073298	19.884819
H	9.356441	7.796350	19.081454
H	9.239208	8.475238	20.720098
C	7.579783	6.000204	19.397664
H	8.413561	5.752250	18.725812
H	6.760904	6.412411	18.791719
C	6.056433	10.665818	20.079322
H	5.329674	10.273836	19.352759
H	6.590581	11.506537	19.613488
C	4.918077	6.338336	26.643755
H	4.273839	6.963155	27.269306
H	5.963296	6.550509	26.871756
H	4.693068	5.294795	26.880743
O	0.039453	6.855319	19.967868
C	-0.828180	7.770518	19.310809
H	-0.297891	8.685371	19.031762
H	-1.176211	7.256493	18.414426
H	-1.680466	8.030667	19.944770
C	0.392849	4.943955	24.327132
C	-0.515707	3.927425	24.623572
C	0.063416	6.270388	24.618363
C	-1.740878	4.211935	25.221431
H	-0.288431	2.896982	24.370637
C	-1.172008	6.564426	25.196210
C	-2.074931	5.552285	25.511565
H	-1.456156	7.594424	25.385996
C	-3.405157	5.891677	26.131793
H	-4.234587	5.555624	25.499972
H	-3.524368	5.401028	27.103965
H	-3.507742	6.968720	26.281295
C	-2.708457	3.099022	25.529572
H	-2.942730	3.063930	26.599118
H	-3.656467	3.239321	24.998776
H	-2.299020	2.128521	25.240676

1_phenOMe.log

SCF (wB97x) = SCF
E(SCF)+ZPE(0 K)= -3072.309455
H(298 K)= -3072.261195
G(298 K)= -3072.393273
Lowest Frequency = 13.6590cm-1

Co	2.438158	6.440646	23.207362
K	5.681061	7.584135	21.870961
O	1.346080	6.357770	21.628831
O	3.169933	8.109164	22.703244
O	3.835555	5.634893	22.188736
O	4.550321	10.116317	21.829681
O	3.419520	6.320474	24.854926
O	5.959394	5.020946	20.823500
O	8.004017	6.927309	20.382725
O	6.963548	9.656237	20.457503
O	5.506126	7.057757	24.436785
O	0.350608	8.390479	21.584978
N	1.658087	4.762192	23.708648
N	1.048083	7.210159	24.257556
C	4.175229	4.393161	22.217502
C	5.352145	4.000138	21.489218
C	2.934161	9.235397	23.283083
C	3.697784	10.376144	22.859591
C	0.600581	7.276288	21.145901
C	1.050119	8.423356	24.693035
H	0.283578	8.718970	25.409883
C	3.946051	2.027912	22.884001
H	3.384444	1.267000	23.418969
C	4.629066	6.605724	25.176636
C	1.830315	10.724736	24.906787
H	1.085166	10.854314	25.687020
C	5.793903	2.694665	21.494318
H	6.690318	2.415306	20.952416
C	3.469807	3.365883	22.901292
C	2.613514	11.780902	24.513253
H	2.513105	12.753858	24.981202
C	2.231079	3.613981	23.575799
H	1.742495	2.735939	23.999136
C	5.328000	11.177033	21.302732
H	6.045123	11.530564	22.054887
H	4.683604	12.013611	21.003819
C	1.969514	9.448675	24.301953
C	3.551929	11.602635	23.471639
H	4.155108	12.448033	23.159845
C	7.126421	4.735456	20.070224
H	6.908316	3.983214	19.301396
H	7.917910	4.355868	20.728839
C	5.088901	1.695292	22.203172
H	5.454857	0.674739	22.193299
C	7.717276	9.152398	19.372397
H	8.315392	9.955919	18.917356
H	7.045130	8.754981	18.597178
C	8.660710	8.079919	19.885295
H	9.358461	7.804698	19.082174
H	9.239106	8.481463	20.721557
C	7.581897	6.007039	19.394757
H	8.415379	5.761109	18.721801
H	6.762171	6.419579	18.790199
C	6.051762	10.666624	20.079604
H	5.326055	10.271900	19.353472
H	6.583299	11.508565	19.612996
C	4.927212	6.331678	26.643949
H	4.283760	6.956031	27.270765
H	5.972712	6.543957	26.870533
H	4.702742	5.287955	26.880629
O	0.044791	6.849458	19.972201
C	-0.824618	7.763864	19.316195
H	-0.296256	8.680470	19.039315
H	-1.170730	7.250660	18.418617
H	-1.677943	8.020677	19.950087
C	0.402505	4.933584	24.330490
C	-0.494454	3.899941	24.619773
C	0.075468	6.258110	24.625475
C	-1.714678	4.188301	25.222127
H	-0.257789	2.880225	24.346620

C	-1.156368	6.548395	25.219122
C	-2.040867	5.530330	25.527544
H	-1.466833	7.565143	25.431485
O	-2.654851	3.269341	25.519906
O	-3.260239	5.842552	26.052539
C	-2.386930	1.905005	25.236970
H	-3.265360	1.354964	25.570957
H	-2.244015	1.745503	24.162511
H	-1.505520	1.554602	25.785425
C	-3.410585	5.537121	27.439398
H	-4.421105	5.840855	27.713002
H	-3.286298	4.465206	27.620189
H	-2.683282	6.102710	28.033030

1_phen_z.log

SCF (wB97x) = SCF
E(SCF)+ZPE(0 K)= -2843.351295
H(298 K)= -2843.308526
G(298 K)= -2843.428084
Lowest Frequency = 16.6599cm-1

Co	2.439581	6.407582	23.100247
K	5.695762	7.589325	21.832867
O	1.366991	6.371348	21.506523
O	3.176613	8.091724	22.655182
O	3.850080	5.629961	22.077878
O	4.569614	10.124131	21.865628
O	3.400684	6.243482	24.755832
O	6.001474	5.053492	20.738611
O	8.047908	6.976706	20.379938
O	7.002833	9.701490	20.516373
O	5.489508	6.997705	24.381915
O	0.363911	8.400632	21.521364
N	1.656015	4.718514	23.546647
N	1.035530	7.144717	24.153207
C	4.199294	4.390626	22.093001
C	5.390810	4.017830	21.377806
C	2.940222	9.195759	23.273604
C	3.710548	10.349375	22.897703
C	0.621908	7.302560	21.047530
C	1.040819	8.341189	24.635736
H	0.269851	8.612730	25.357237
C	3.977511	2.013427	22.719202
H	3.413210	1.240155	23.233151
C	4.604962	6.525630	25.099930
C	1.829862	10.628136	24.945141
H	1.080289	10.731250	25.725034
C	5.842110	2.715705	21.369863
H	6.749187	2.451450	20.838198
C	3.490805	3.347488	22.749460
C	2.619051	11.694681	24.596208
H	2.519158	12.649997	25.099200
C	2.238750	3.575996	23.405529
H	1.746377	2.689038	23.805133
C	5.359844	11.198771	21.387445
H	6.067408	11.522013	22.161921
H	4.723583	12.048457	21.108778
C	1.968426	9.374308	24.293475
C	3.564000	11.551996	23.554069
H	4.171762	12.406389	23.277572
C	7.184525	4.789443	20.002555
H	6.985176	4.052804	19.213770
H	7.964836	4.399474	20.668372
C	5.133544	1.700098	22.051739
H	5.507530	0.682620	22.031917
C	7.769084	9.227661	19.426409
H	8.370882	10.043747	18.999483
H	7.106068	8.849790	18.633703
C	8.708509	8.143177	19.921297
H	9.416095	7.890656	19.119413
H	9.276340	8.523143	20.774725
C	7.647671	6.077317	19.363910
H	8.494593	5.848903	18.701646
H	6.838235	6.500851	18.753127
C	6.099694	10.726840	20.158410
H	5.383479	10.358809	19.409217
H	6.640574	11.581803	19.727676
C	4.885357	6.219151	26.564221
H	4.230126	6.825207	27.196759
H	5.926540	6.431863	26.809402
H	4.664066	5.168972	26.773739
O	0.077775	6.913033	19.855788
C	-0.790335	7.845552	19.223876
H	-0.264842	8.774579	18.985766
H	-1.124216	7.362931	18.304967
H	-1.651660	8.074477	19.857678
C	0.385284	4.867261	24.145862
C	-0.524081	3.835880	24.384840
C	0.048016	6.189323	24.473557
C	-1.754622	4.123504	24.966138
H	-0.296092	2.814384	24.102188
C	-1.198091	6.474376	25.033801
C	-2.090982	5.438911	25.288962
H	-2.461778	3.320682	25.145296
H	-1.490156	7.495851	25.249747
H	-3.061150	5.663204	25.719286

2_phenCl_PO.log

SCF (wB97x) = SCF
E(SCF)+ZPE(0 K)= -3955.584554
H(298 K)= -3955.533544
G(298 K)= -3955.671687
Lowest Frequency = 16.9482cm-1

Co	2.483475	6.297041	23.139212
K	5.563113	7.577077	21.571003
O	4.755661	7.852700	19.000322
O	3.120899	8.017467	22.666059
O	3.868142	5.532362	22.107263
O	4.604779	10.069262	22.045289
O	3.428302	6.207527	24.762644
O	6.072178	5.009065	20.832845
O	8.112632	6.973504	20.460092
O	7.096169	9.746139	20.698033
O	5.474498	6.976885	24.212000
O	3.509331	9.622001	18.381044
N	1.724649	4.596244	23.573447
N	1.012455	7.015934	24.121379
C	4.288845	4.313166	22.186581
C	5.504870	3.974178	21.500216
C	2.915695	9.099088	23.349400
C	3.718201	10.251637	23.052933
C	3.824737	8.421361	18.392988
C	0.982801	8.205604	24.629533
H	0.182023	8.453953	25.326114
C	4.171366	1.955372	22.911019
H	3.638221	1.178203	23.450944
C	4.655648	6.535744	25.012257
C	1.773649	10.469856	25.057148
H	1.003597	10.546575	25.819287
C	6.013092	2.693641	21.548275
H	6.935840	2.452317	21.032919
C	3.623494	3.266932	22.879610
C	2.590260	11.535248	24.780982
H	2.490974	12.468098	25.324277
C	2.349122	3.465897	23.489232
H	1.871859	2.575924	23.899325
C	5.403334	11.164623	21.616943
H	6.099762	11.453721	22.414532
H	4.764622	12.022187	21.370782

C 1.919083 9.245858 24.350160
C 3.565557 11.423295 23.765120
H 4.197345 12.277603 23.550223
C 7.257417 4.782703 20.083851
H 7.068933 4.039031 19.299163
H 8.056493 4.418223 20.741624
C 5.346487 1.673720 22.265363
H 5.768840 0.675551 22.288704
C 7.764300 9.240650 19.555585
H 8.334694 10.044612 19.065224
H 7.024600 8.849546 18.842793
C 8.735088 8.156534 19.987326
H 9.401640 7.921085 19.145471
H 9.345974 8.526799 20.815377
C 7.668694 6.089118 19.444853
H 8.485318 5.884465 18.737842
H 6.819132 6.517755 18.895248
C 6.148458 10.746635 20.371840
H 5.434870 10.365898 19.627814
H 6.658516 11.631869 19.962510
C 5.016962 6.326046 26.473279
H 4.418099 6.997905 27.095136
H 6.075894 6.529330 26.635071
H 4.784065 5.301755 26.775318
O 3.050110 7.538339 17.645100
C 1.911992 8.071185 16.989818
H 2.188946 8.841165 16.263307
H 1.439049 7.233080 16.474362
H 1.206528 8.503600 17.707595
C 0.427818 4.721783 24.110825
C -0.462288 3.673150 24.320429
C 0.037674 6.037304 24.400744
C -1.728329 3.931415 24.833947
H -0.203011 2.651991 24.069815
C -1.238264 6.296468 24.889580
C -2.117844 5.244238 25.117455
H -1.576432 7.308592 25.075426
C 1.812581 6.488727 20.191557
C 0.876373 7.440740 20.790705
H 2.869297 6.729055 20.146012
H 1.448587 5.750531 19.483333
O 1.313254 6.268962 21.532203
H 1.312375 8.355587 21.181878
C -0.587821 7.466793 20.474661
H -0.778821 8.225407 19.710108
H -0.927047 6.497456 20.101343
H -1.163192 7.728822 21.367034
Cl -3.704909 5.605043 25.723430
Cl -2.812764 2.596577 25.076388

2_phenF_PO.log

SCF (wB97x) = SCF
E(SCF)+ZPE(0 K)= -3234.848721
H(298 K)= -3234.798489
G(298 K)= -3234.934193
Lowest Frequency = 16.9465cm-1

Co 2.483145 6.297272 23.139736
K 5.563365 7.577236 21.572420
O 4.763580 7.858280 18.998404
O 3.120921 8.017290 22.665123
O 3.867434 5.533742 22.106068
O 4.605594 10.069518 22.043942
O 3.428615 6.207172 24.762828
O 6.071511 5.010545 20.829959
O 8.114579 6.972982 20.465057
O 7.099464 9.746403 20.701994
O 5.475318 6.976335 24.213177
O 3.510314 9.622377 18.378396
N 1.723868 4.596351 23.573180
N 1.012177 7.015737 24.122841
C 4.286662 4.313266 22.181332
C 5.501883 3.974492 21.493866
C 2.914040 9.100611 23.346185
C 3.716283 10.252980 23.049206
C 3.830150 8.422899 18.391236
C 0.979467 8.207046 24.624800
H 0.176064 8.457625 25.317574
C 4.164467 1.953647 22.896806
H 3.629657 1.175538 23.433775
C 4.655827 6.535153 25.012835
C 1.766572 10.473953 25.047274
H 0.994431 10.551761 25.807221
C 6.007908 2.692659 21.537049
H 6.930239 2.451583 21.020842
C 3.619729 3.266154 22.870445
C 2.582954 11.539864 24.770881
H 2.481163 12.473809 25.311865
C 2.345543 3.465923 23.482594
H 1.866443 2.574923 23.888387
C 5.403622 11.164983 21.615180
H 6.098083 11.456528 22.413606
H 4.764427 12.021348 21.366065
C 1.915215 9.248780 24.343829
C 3.560885 11.426508 23.758158
H 4.192462 12.280839 23.542760
C 7.257115 4.784322 20.081646
H 7.068366 4.043228 19.294561
H 8.054843 4.416526 20.739227
C 5.339267 1.671643 22.250033
H 5.759377 0.672430 22.269754
C 7.772042 9.241589 19.561918
H 8.345248 10.045595 19.074896
H 7.035108 8.851993 18.845483
C 8.739984 8.156051 19.996463
H 9.409928 7.921105 19.157137
H 9.347661 8.524687 20.827597
C 7.671327 6.091767 19.446737
H 8.489000 5.887900 18.740692
H 6.823312 6.522926 18.896751
C 6.151854 10.745819 20.372347
H 5.440337 10.363443 19.627198
H 6.662213 11.630893 19.963019
C 5.016576 6.325647 26.474123
H 4.419948 7.000223 27.095222
H 6.076132 6.525880 26.635699
H 4.780434 5.302489 26.777441
O 3.058153 7.536360 17.644689
C 1.917783 8.064598 16.989599
H 2.191544 8.835327 16.262678
H 1.447795 7.224495 16.474690
H 1.210929 8.494702 17.707430
C 0.428114 4.720966 24.115741
C -0.456697 3.663542 24.331007
C 0.038249 6.036246 24.406944
C -1.707986 3.936604 24.849124
H -0.213300 2.637018 24.085395
C -1.236375 6.301612 24.907794
C -2.095360 5.243615 25.133752
H -1.589588 7.307141 25.102243
C 1.815405 6.494740 20.193180
C 0.875298 7.442837 20.792257
H 2.871637 6.737631 20.151500
H 1.455339 5.757714 19.481742
O 1.312944 6.270124 21.531734
H 1.307991 8.357670 21.187081
C -0.587842 7.466567 20.471218
H -0.777582 8.226419 19.707563
H -0.923788 6.497387 20.094519
H -1.166962 7.725627 21.362031
F -2.577417 2.944399 25.054410

F -3.325086 5.467792 25.602669

2_phenMe_PO.log

SCF (wB97x) = SCF
E(SCF)+ZPE(0 K)= -3114.922294
H(298 K)= -3114.870601
G(298 K)= -3115.008578
Lowest Frequency = 17.6991cm-1

Co	2.517309	6.216844	23.194203
K	5.534255	7.558022	21.564869
O	4.690564	7.864817	18.998818
O	3.111722	7.954182	22.714323
O	3.888829	5.484101	22.118207
O	4.565021	10.034419	22.095654
O	3.506758	6.133552	24.793624
O	6.075856	5.006750	20.792965
O	8.085665	6.999849	20.411311
O	7.042846	9.755016	20.711194
O	5.526877	6.941841	24.202173
O	3.466516	9.665896	18.427263
N	1.797659	4.500623	23.632761
N	1.058457	6.902240	24.216860
C	4.333505	4.270572	22.179456
C	5.539035	3.957132	21.464720
C	2.911274	9.025695	23.418360
C	3.695294	10.190432	23.123777
C	3.746702	8.456413	18.434740
C	1.016300	8.086823	24.727670
H	0.220025	8.318731	25.435063
C	4.270140	1.908028	22.885367
H	3.760916	1.117815	23.429763
C	4.732433	6.481762	25.017181
C	1.788460	10.358033	25.165165
H	1.032041	10.415236	25.942727
C	6.070254	2.684306	21.489403
H	6.984965	2.463064	20.951152
C	3.701884	3.209166	22.877712
C	2.588489	11.438020	24.889923
H	2.488807	12.361538	25.449067
C	2.434406	3.383440	23.519436
H	1.976094	2.481519	23.925287
C	5.352759	11.142580	21.681750
H	6.056989	11.418344	22.477382
H	4.707336	12.002303	21.461643
C	1.934445	9.147235	24.439707
C	3.544740	11.351475	23.855528
H	4.163409	12.215402	23.641071
C	7.249158	4.805326	20.019626
H	7.056074	4.069446	19.228585
H	8.065465	4.442768	20.657112
C	5.436355	1.648983	22.211526
H	5.873993	0.657053	22.217498
C	7.695768	9.276163	19.548782
H	8.248537	10.094477	19.061880
H	6.947528	8.888657	18.842897
C	8.686193	8.196451	19.945076
H	9.338970	7.981261	19.087086
H	9.308912	8.560927	20.766892
C	7.632820	6.125341	19.391495
H	8.438344	5.940787	18.666376
H	6.768412	6.552021	18.863813
C	6.085828	10.757339	20.419183
H	5.366150	10.390794	19.673899
H	6.586784	11.654721	20.025068
C	5.130927	6.271180	26.468960
H	4.534935	6.929650	27.107755
H	6.189628	6.491657	26.608562
H	4.922361	5.241619	26.771019
O	2.908463	7.591614	17.735549
C	1.751248	8.152015	17.139752
H	2.008966	8.899032	16.382691
H	1.220382	7.321812	16.669781
H	1.105704	8.620911	17.890178
C	0.505769	4.601279	24.199679
C	-0.368580	3.538192	24.424272
C	0.101944	5.902228	24.507368
C	-1.633418	3.754123	24.966331
H	-0.084269	2.526259	24.154850
C	-1.170958	6.129326	25.028741
C	-2.042190	5.070334	25.271783
H	-1.508880	7.141309	25.227956
C	1.777911	6.475906	20.274750
C	0.802909	7.352753	20.924455
H	2.819002	6.774320	20.221796
H	1.442085	5.747172	19.543312
O	1.314184	6.179337	21.612304
H	1.196263	8.274216	21.344497
C	-0.665825	7.311700	20.631449
H	-0.909916	8.084913	19.897208
H	-0.959041	6.338985	20.228866
H	-1.239184	7.510500	21.541386
C	-3.416138	5.334320	25.829509
H	-4.195714	4.980411	25.146192
H	-3.562610	4.811330	26.780777
H	-3.574243	6.401123	26.001590
C	-2.565319	2.592952	25.194331
H	-2.856576	2.522034	26.247839
H	-3.487550	2.705596	24.613972
H	-2.097493	1.648674	24.907720

2_phenOMe_PO.log

SCF (wB97x) = SCF
E(SCF)+ZPE(0 K)= -3265.316262
H(298 K)= -3265.262396
G(298 K)= -3265.405819
Lowest Frequency = 17.6621cm-1

Co	2.520624	6.221727	23.199718
K	5.538324	7.558747	21.566416
O	4.689160	7.863408	19.002308
O	3.117425	7.958092	22.719180
O	3.889415	5.488182	22.123385
O	4.570950	10.036269	22.095959
O	3.509720	6.139000	24.798848
O	6.073779	5.007154	20.794780
O	8.086594	6.996120	20.409513
O	7.047231	9.752843	20.709110
O	5.530462	6.944269	24.205401
O	3.468122	9.666463	18.430444
N	1.797967	4.507168	23.640218
N	1.061626	6.911054	24.222126
C	4.329156	4.271937	22.178927
C	5.532686	3.956890	21.462156
C	2.918546	9.030096	23.421981
C	3.703133	10.194162	23.125041
C	3.745829	8.456404	18.438782
C	1.024111	8.095997	24.734411
H	0.231911	8.330215	25.445603
C	4.254709	1.906296	22.871769
H	3.741858	1.115478	23.411851
C	4.736138	6.486046	25.021385
C	1.799922	10.365378	25.170208
H	1.045020	10.423915	25.949118
C	6.058424	2.681475	21.480051
H	6.972095	2.459071	20.940541
C	3.692919	3.210001	22.871531
C	2.600358	11.443976	24.892548
H	2.502770	12.368104	25.451010
C	2.427089	3.387034	23.516651

H	1.964146	2.484494	23.915817
C	5.359800	11.143146	21.680265
H	6.065165	11.418484	22.475012
H	4.715178	12.003458	21.460199
C	1.943325	9.153559	24.445071
C	3.554780	11.355591	23.856215
H	4.173873	12.218799	23.640135
C	7.244504	4.803434	20.018106
H	7.047325	4.069159	19.226594
H	8.061400	4.437707	20.653024
C	5.419922	1.645446	22.196542
H	5.852869	0.651441	22.197262
C	7.698782	9.272715	19.546444
H	8.252251	10.090143	19.058874
H	6.949577	8.886005	18.841145
C	8.688141	8.191865	19.942393
H	9.339965	7.975592	19.083942
H	9.311955	8.555850	20.763586
C	7.629882	6.123018	19.390197
H	8.433458	5.937263	18.663232
H	6.765234	6.551742	18.864580
C	6.090983	10.755946	20.417240
H	5.370026	10.389571	19.673100
H	6.592464	11.652460	20.021840
C	5.134932	6.276439	26.473160
H	4.538593	6.934697	27.111829
H	6.193521	6.497639	26.612387
H	4.927187	5.246819	26.775595
O	2.905021	7.592676	17.741463
C	1.748546	8.154927	17.145983
H	2.007335	8.899529	16.386896
H	1.214779	7.325148	16.678567
H	1.105329	8.627317	17.896191
C	0.509785	4.613512	24.210472
C	-0.363339	3.548970	24.450335
C	0.105511	5.913332	24.514860
C	-1.615543	3.781422	24.991421
H	-0.107765	2.525202	24.202087
C	-1.166129	6.162948	25.038291
C	-2.032044	5.101984	25.283656
H	-1.491914	7.179575	25.215756
C	1.777647	6.475171	20.279926
C	0.807438	7.357940	20.928818
H	2.819771	6.769472	20.223830
H	1.437399	5.745235	19.551739
O	1.315651	6.184757	21.619546
H	1.205354	8.279283	21.344830
C	-0.662168	7.321668	20.639619
H	-0.905014	8.093189	19.903198
H	-0.960282	6.348689	20.241346
H	-1.232494	7.526053	21.550242
O	-3.284285	5.234280	25.759914
O	-2.472186	2.734938	25.157148
C	-3.758522	6.533791	26.078789
H	-3.789370	7.171391	25.188524
H	-4.768825	6.394838	26.460151
H	-3.136716	7.000824	26.850491
C	-2.677096	2.333372	26.512939
H	-3.107579	3.148320	27.102193
H	-3.372804	1.494917	26.481844
H	-1.729959	2.009104	26.958687

2_phen_PO.log

SCF (wB97x) = SCF
E(SCF)+ZPE(0 K)= -3036.357599
H(298 K)= -3036.309276
G(298 K)= -3036.440194
Lowest Frequency = 15.5722cm-1

Co	2.480110	6.295678	23.134644
K	5.555521	7.574591	21.559263
O	4.773038	7.860826	18.977268
O	3.115544	8.017103	22.654104
O	3.861804	5.530918	22.095400
O	4.604293	10.069266	22.035673
O	3.432956	6.210557	24.755431
O	6.070057	5.006913	20.824225
O	8.115626	6.969626	20.465846
O	7.102129	9.742405	20.701752
O	5.480574	6.972121	24.198243
O	3.524895	9.631084	18.364354
N	1.723639	4.596986	23.574512
N	1.012532	7.012988	24.120631
C	4.287471	4.312749	22.180860
C	5.503931	3.973497	21.496405
C	2.914770	9.098998	23.340448
C	3.718577	10.250409	23.045327
C	3.838460	8.429903	18.376052
C	0.982769	8.202496	24.623502
H	0.179973	8.452626	25.317115
C	4.175159	1.959906	22.915409
H	3.643536	1.183987	23.458832
C	4.660839	6.536297	25.001177
C	1.777794	10.467191	25.050838
H	1.008668	10.543611	25.814080
C	6.015604	2.693851	21.549025
H	6.938565	2.452400	21.034083
C	3.625463	3.269177	22.878801
C	2.595538	11.533302	24.775885
H	2.497551	12.465132	25.321305
C	2.348433	3.469337	23.490219
H	1.870443	2.579496	23.899754
C	5.408117	11.162999	21.614374
H	6.101408	11.448263	22.416158
H	4.773585	12.023395	21.366999
C	1.920864	9.245196	24.342442
C	3.568934	11.422104	23.759280
H	4.201539	12.275864	23.544609
C	7.259595	4.780578	20.082798
H	7.076304	4.037050	19.296648
H	8.054766	4.415777	20.745194
C	5.351357	1.676502	22.270300
H	5.774982	0.678907	22.298127
C	7.776633	9.238487	19.562444
H	8.351532	10.042622	19.077577
H	7.040858	8.850108	18.844150
C	8.742908	8.151986	19.998168
H	9.413830	7.916461	19.159772
H	9.349783	8.520048	20.830170
C	7.675969	6.087035	19.447127
H	8.496278	5.882152	18.744417
H	6.830218	6.517679	18.893223
C	6.159126	10.746224	20.372416
H	5.449041	10.369303	19.623155
H	6.674033	11.631055	19.968242
C	5.025078	6.331828	26.462612
H	4.429129	7.007622	27.083053
H	6.084835	6.533212	26.621646
H	4.790456	5.309334	26.769493
O	3.056373	7.546905	17.635728
C	1.913087	8.080312	16.989958
H	2.184389	8.848844	16.259777
H	1.434465	7.242043	16.479999
H	1.214682	8.514618	17.713508
C	0.422498	4.718818	24.114870
C	-0.467755	3.665845	24.327579
C	0.032273	6.034288	24.403970
C	-1.732738	3.929295	24.842810
H	-0.198826	2.646929	24.073452
C	-1.246001	6.295752	24.896686
C	-2.121763	5.239516	25.124736

H -2.425868 3.110053 25.000545
H -1.575065 7.312629 25.079136
H -3.118323 5.442714 25.501491
C 1.807488 6.521908 20.194561
C 0.847895 7.441254 20.807511
H 2.858698 6.786002 20.158784
H 1.463638 5.789816 19.470153
O 1.308207 6.265869 21.527837
H 1.261761 8.358003 21.217915
C -0.615115 7.440690 20.484737
H -0.819509 8.208153 19.732564
H -0.931011 6.470780 20.092799
H -1.200145 7.673841 21.378891

3_phenCl_TS_2.log

SCF (wB97x) = SCF
E(SCF)+ZPE(0 K)= -3955.563485
H(298 K)= -3955.513262
G(298 K)= -3955.649207
Lowest Frequency = -540.2044cm-1

Co 2.471471 6.319677 23.216804
K 5.633991 7.565025 21.737505
O 3.154174 8.024532 22.729958
O 3.899487 5.543864 22.238101
O 4.579680 10.069778 21.985485
O 3.430401 6.259065 24.873681
O 6.062305 4.987452 20.925300
O 8.092495 6.935430 20.488896
O 7.068114 9.707542 20.652711
O 5.481491 7.046848 24.375786
N 1.719186 4.630067 23.700772
N 1.018689 7.067168 24.201551
C 4.275111 4.313934 22.284490
C 5.476580 3.952412 21.579533
C 2.920215 9.131184 23.352011
C 3.698410 10.285414 22.994998
C 0.990072 8.273089 24.667506
H 0.195202 8.544318 25.362706
C 4.101011 1.946870 22.965075
H 3.552366 1.173631 23.495423
C 4.640757 6.597239 25.154978
C 1.755969 10.564105 24.991645
H 0.984658 10.665377 25.749880
C 5.952093 2.660046 21.599633
H 6.864459 2.403904 21.072725
C 3.585745 3.272684 22.964162
C 2.556084 11.628205 24.666286
H 2.443144 12.583283 25.166723
C 2.325944 3.492138 23.598816
H 1.845194 2.606438 24.014637
C 5.370634 11.153476 21.519938
H 6.063487 11.477137 22.307338
H 4.728362 11.998189 21.240268
C 1.917256 9.310247 24.342465
C 3.530150 11.485900 23.651648
H 4.144768 12.340919 23.393745
C 7.222881 4.743505 20.145889
H 7.004978 4.000326 19.368183
H 8.034809 4.369842 20.782664
C 5.262520 1.644857 22.304315
H 5.657446 0.635181 22.306150
C 7.756337 9.182961 19.530560
H 8.335406 9.978950 19.037561
H 7.029229 8.781965 18.811167
C 8.720391 8.106942 19.996144
H 9.394523 7.852665 19.165787
H 9.323856 8.493996 20.821876
C 7.631250 6.042336 19.489587
H 8.436409 5.828307 18.772290
H 6.775830 6.469448 18.948352
C 6.126085 10.699145 20.293936
H 5.426592 10.298371 19.548465
H 6.638635 11.572296 19.862706
C 4.976223 6.400757 26.626196
H 4.380113 7.093787 27.227797
H 6.035631 6.587046 26.806346
H 4.717707 5.386312 26.940665
C 0.434086 4.772416 24.258823
C 0.050321 6.096515 24.522573
C -0.454337 3.731787 24.513593
H -0.201833 2.703490 24.286427
C -1.710213 4.004506 25.044019
C -2.092968 5.324319 25.300850
C -1.216430 6.368463 25.028830
H -1.550508 7.385172 25.196146
O 4.707527 7.828121 19.120993
C 3.759853 8.287423 18.470540
O 2.543506 7.948595 18.514452
O 4.087462 9.291963 17.599687
C 3.036859 9.892463 16.850844
H 2.298059 10.356061 17.510493
H 3.512078 10.656160 16.234067
H 2.536793 9.158862 16.212795
H 1.319030 8.307318 21.354663
C 1.042050 7.368012 20.875000
C 2.106052 6.741368 20.100842
O 1.335392 6.220369 21.660924
C -0.368849 7.380148 20.341997
H 3.127447 6.848432 20.425377
H 1.846431 5.923237 19.442726
H -0.483609 8.182129 19.606641
H -0.613166 6.425945 19.865844
H -1.072887 7.555706 21.161010
Cl -3.668122 5.703564 25.928787
Cl -2.790915 2.676925 25.343060

3_phenF_TS_2.log

SCF (wB97x) = SCF
E(SCF)+ZPE(0 K)= -3234.827526
H(298 K)= -3234.778092
G(298 K)= -3234.911878
Lowest Frequency = -540.0194cm-1

Co 2.471666 6.319145 23.217242
K 5.635187 7.563662 21.738736
O 3.154618 8.023445 22.727898
O 3.900162 5.543985 22.238395
O 4.581623 10.068688 21.982843
O 3.430845 6.259877 24.874393
O 6.062346 4.986944 20.923603
O 8.094942 6.932765 20.490828
O 7.072098 9.705609 20.654260
O 5.482042 7.048216 24.377298
N 1.719430 4.629686 23.702089
N 1.018639 7.066683 24.201973
C 4.274043 4.312921 22.281309
C 5.474431 3.951174 21.575106
C 2.918827 9.131898 23.347017
C 3.697158 10.285650 22.989543
C 0.986821 8.274202 24.661222
H 0.189267 8.547582 25.352532
C 4.095427 1.944832 22.954710
H 3.545347 1.171109 23.482927
C 4.640524 6.599465 25.156252
C 1.748925 10.567680 24.979205
H 0.975305 10.670235 25.734949
C 5.947693 2.657659 21.590963
H 6.859458 2.401449 21.063047
C 3.583297 3.271368 22.958088

C	2.549154	11.632046	24.653644
H	2.433771	12.588213	25.151484
C	2.323817	3.491893	23.595469
H	1.841732	2.605527	24.008448
C	5.372994	11.152165	21.517771
H	6.064133	11.477013	22.306210
H	4.731029	11.996395	21.235902
C	1.913412	9.312664	24.334029
C	3.526265	11.487973	23.642669
H	4.141140	12.342710	23.384478
C	7.222378	4.742615	20.143712
H	7.003313	4.001391	19.364437
H	8.033734	4.366208	20.779623
C	5.256335	1.642009	22.292540
H	5.648939	0.631404	22.291310
C	7.762300	9.181163	19.533330
H	8.342968	9.977001	19.041954
H	7.036410	8.781183	18.812135
C	8.724605	8.104128	20.000141
H	9.400196	7.849942	19.170918
H	9.326725	8.490179	20.827321
C	7.632912	6.041957	19.489838
H	8.438101	5.828053	18.772517
H	6.778294	6.471136	18.948986
C	6.130930	10.697314	20.293543
H	5.432866	10.296514	19.546776
H	6.644488	11.570293	19.863130
C	4.974495	6.407471	26.628503
H	4.387036	7.111756	27.225646
H	6.036005	6.583040	26.807141
H	4.704166	5.398374	26.949977
C	0.435433	4.771379	24.265298
C	0.051455	6.095343	24.528723
C	-0.447539	3.722251	24.526292
H	-0.210495	2.688634	24.305548
C	-1.688993	4.009902	25.059893
C	-2.070031	5.324055	25.316607
C	-1.214088	6.373989	25.045810
H	-1.563242	7.384555	25.220673
O	4.711304	7.828740	19.119368
C	3.762977	8.290659	18.471829
O	2.545850	7.954999	18.519003
O	4.090600	9.294795	17.600410
C	3.039360	9.898446	16.855029
H	2.303739	10.363632	17.517113
H	3.514732	10.661234	16.237240
H	2.535461	9.166518	16.218068
H	1.313383	8.306051	21.357372
C	1.041180	7.366438	20.875494
C	2.109478	6.745154	20.102940
O	1.336310	6.218834	21.660583
C	-0.368121	7.374702	20.338095
H	3.129380	6.855531	20.430916
H	1.854713	5.926662	19.443398
H	-0.482814	8.176746	19.602781
H	-0.608079	6.420070	19.860569
H	-1.075413	7.547823	21.154828
F	-3.290748	5.562965	25.803932
F	-2.554650	3.023290	25.309079

3_phenMe_TS_3.log

SCF (wB97x) = SCF
E(SCF)+ZPE(0 K)= -3114.900409
H(298 K)= -3114.850362
G(298 K)= -3114.982839
Lowest Frequency = -539.7308cm-1

Co	2.467923	6.315699	23.206689
K	5.633526	7.558648	21.738746
O	3.153922	8.021837	22.718259
O	3.898508	5.541786	22.225694
O	4.589887	10.067223	21.984185
O	3.428367	6.254372	24.865418
O	6.065171	4.985133	20.915412
O	8.104025	6.927268	20.500181
O	7.085608	9.702092	20.667748
O	5.481787	7.043906	24.377536
N	1.714742	4.626111	23.689145
N	1.015165	7.063187	24.191560
C	4.270330	4.308728	22.263911
C	5.471461	3.947291	21.560109
C	2.918355	9.130831	23.338810
C	3.700191	10.283432	22.987515
C	0.980739	8.269869	24.646528
H	0.179563	8.543527	25.333495
C	4.082871	1.939822	22.923490
H	3.528760	1.164940	23.446037
C	4.635850	6.594143	25.151975
C	1.744108	10.564706	24.966336
H	0.966960	10.667141	25.718651
C	5.942060	2.651973	21.569438
H	6.854749	2.396457	21.042725
C	3.575520	3.266582	22.933068
C	2.547481	11.629975	24.646704
H	2.430466	12.585275	25.146007
C	2.313778	3.489016	23.573589
H	1.826823	2.601841	23.978955
C	5.384864	11.150187	21.525774
H	6.072472	11.472877	22.318291
H	4.745945	11.996218	21.242079
C	1.909744	9.311543	24.320916
C	3.528838	11.486211	23.641126
H	4.146562	12.340083	23.386806
C	7.226764	4.741153	20.138752
H	7.008671	4.004680	19.354586
H	8.034970	4.358733	20.775208
C	5.245661	1.635498	22.262785
H	5.634376	0.623313	22.257677
C	7.780461	9.179593	19.549005
H	8.365180	9.975686	19.062800
H	7.057513	8.783295	18.822788
C	8.738272	8.099226	20.017317
H	9.418079	7.846919	19.190909
H	9.336423	8.481382	20.849185
C	7.643792	6.042500	19.493054
H	8.451407	5.830124	18.777941
H	6.792616	6.476559	18.950665
C	6.148611	10.696912	20.304608
H	5.453736	10.299567	19.553020
H	6.666591	11.569751	19.879136
C	4.964223	6.404517	26.626303
H	4.395282	7.130036	27.216187
H	6.029654	6.555074	26.804905
H	4.667235	5.406125	26.957221
C	0.428722	4.769678	24.258324
C	0.046508	6.088085	24.520140
C	-0.461642	3.730386	24.529710
H	-0.194847	2.703852	24.301000
C	-1.719984	3.984977	25.069801
C	-2.107239	5.317597	25.326831
C	-1.220776	6.352311	25.038925
H	-1.543018	7.375623	25.203024
O	4.721966	7.838637	19.109480
C	3.772262	8.312444	18.473096
O	2.552975	7.984706	18.525699
O	4.099896	9.322384	17.608195
C	3.047036	9.940274	16.876961
H	2.319361	10.403291	17.549268
H	3.522663	10.706327	16.263407
H	2.533704	9.218141	16.236337
H	1.304119	8.307495	21.359315

C 1.040108 7.370068 20.868446
C 2.115894 6.763270 20.094152
O 1.336120 6.218875 21.646138
C -0.366442 7.375731 20.323114
H 3.132585 6.873682 20.431534
H 1.868915 5.947632 19.428095
H -0.482139 8.183942 19.594659
H -0.598556 6.424208 19.835493
H -1.078997 7.536954 21.137775
C -3.473279 5.624290 25.882748
H -3.620311 5.144340 26.856421
H -3.614933 6.699650 26.010803
H -4.263345 5.253735 25.220574
C -2.666490 2.846599 25.348566
H -2.951951 2.820043 26.405779
H -3.590965 2.949177 24.769808
H -2.213507 1.885456 25.095832

3_phenOMe_TS_3.log

SCF (wB97x) = SCF
E(SCF)+ZPE(0 K)= -3265.291636
H(298 K)= -3265.239069
G(298 K)= -3265.378493
Lowest Frequency = -539.9178cm-1

Co 2.460330 6.352898 23.206278
K 5.645138 7.549339 21.739073
O 3.167555 8.047085 22.710168
O 3.884296 5.556489 22.235626
O 4.627169 10.070554 21.964464
O 3.412753 6.290068 24.868489
O 6.043397 4.966576 20.928701
O 8.106201 6.880501 20.504463
O 7.120273 9.668061 20.653997
O 5.477097 7.049683 24.379984
N 1.684541 4.674799 23.695670
N 1.013077 7.124396 24.180169
C 4.236752 4.318253 22.274894
C 5.433621 3.938352 21.573081
C 2.941608 9.163078 23.321081
C 3.737142 10.304421 22.963183
C 0.990236 8.334866 24.627737
H 0.189742 8.621382 25.310400
C 4.011769 1.951755 22.934254
H 3.444938 1.185442 23.455685
C 4.624275 6.615993 25.155701
C 1.777340 10.622874 24.933798
H 0.998886 10.739682 25.682577
C 5.884133 2.636198 21.583923
H 6.793815 2.366605 21.059045
C 3.524586 3.286627 22.942881
C 2.593462 11.676155 24.608488
H 2.485573 12.636651 25.099757
C 2.266318 3.527881 23.580388
H 1.765229 2.647800 23.983935
C 5.434787 11.141377 21.499143
H 6.124732 11.462185 22.290344
H 4.805504 11.992167 21.208347
C 1.931442 9.362633 24.298464
C 3.576630 11.513669 23.607115
H 4.204414 12.358874 23.348598
C 7.200829 4.704318 20.151435
H 6.971602 3.968476 19.369893
H 8.004173 4.312753 20.788432
C 5.170833 1.630212 22.276101
H 5.544330 0.612334 22.271697
C 7.812412 9.131510 19.540212
H 8.408243 9.918047 19.051983
H 7.087158 8.740276 18.813589
C 8.755745 8.042110 20.016937
H 9.434509 7.777169 19.193618
H 9.356374 8.421391 20.848327
C 7.634684 5.997683 19.500792
H 8.439578 5.771936 18.786708
H 6.789170 6.440649 18.956776
C 6.195003 10.670675 20.282393
H 5.496689 10.275681 19.532750
H 6.723253 11.534573 19.851371
C 4.946836 6.430825 26.631705
H 6.012902 6.573993 26.812441
H 4.641539 5.436056 26.966011
H 4.381619 7.162988 27.216866
C 0.400947 4.836915 24.261282
C 0.033037 6.164649 24.512232
C -0.493492 3.806196 24.548569
H -0.257413 2.766090 24.355969
C -1.743909 4.090579 25.083596
C -2.124996 5.428776 25.302867
C -1.233816 6.455037 25.016212
H -1.572738 7.474013 25.165305
O 4.741235 7.818979 19.108498
C 3.800401 8.293724 18.459641
O 2.577874 7.977868 18.507889
O 4.143697 9.289604 17.584577
C 3.101871 9.908366 16.838410
H 2.375878 10.389628 17.499684
H 3.589448 10.659861 16.216292
H 2.583814 9.182661 16.205715
H 1.330154 8.345725 21.337397
C 1.052861 7.407754 20.855077
C 2.120614 6.777143 20.088904
O 1.331442 6.259274 21.643770
C -0.353017 7.428430 20.308596
H 3.138735 6.877002 20.425573
H 1.862609 5.959304 19.429780
H -0.456718 8.231629 19.572862
H -0.598298 6.475940 19.829427
H -1.063698 7.607141 21.121250
O -3.350105 5.747851 25.810003
O -2.602379 3.057395 25.317788
C -4.452278 5.522418 24.927527
H -4.336319 6.122398 24.018214
H -4.536490 4.463469 24.666258
H -5.343701 5.842244 25.466625
C -2.897278 2.808841 26.694356
H -3.388723 3.671517 27.153495
H -3.567343 1.949602 26.710866
H -1.977961 2.569596 27.240056

3_phen_TS_3.log

SCF (wB97x) = SCF
E(SCF)+ZPE(0 K)= -3036.336016
H(298 K)= -3036.289357
G(298 K)= -3036.414599
Lowest Frequency = -539.8543cm-1

Co 2.468817 6.316635 23.212400
K 5.629429 7.560646 21.731252
O 3.151159 8.022152 22.718983
O 3.897619 5.541381 22.230739
O 4.581231 10.067748 21.976888
O 3.433592 6.260911 24.868406
O 6.063172 4.983458 20.920152
O 8.096206 6.930018 20.490000
O 7.073108 9.703055 20.651869
O 5.484593 7.049770 24.370175
N 1.719178 4.629048 23.702450
N 1.018187 7.062976 24.198997
C 4.275394 4.311202 22.279834
C 5.476719 3.948936 21.576134

C	2.918640	9.130134	23.341221
C	3.697845	10.283497	22.985495
C	0.988452	8.269127	24.658357
H	0.191848	8.541849	25.350948
C	4.101964	1.947353	22.964147
H	3.553389	1.174896	23.495938
C	4.642395	6.601690	25.149416
C	1.754042	10.562637	24.978035
H	0.981840	10.664268	25.735463
C	5.953350	2.656328	21.597357
H	6.865752	2.399881	21.070603
C	3.587294	3.271992	22.961471
C	2.554692	11.627929	24.653591
H	2.440690	12.582973	25.154013
C	2.325757	3.493594	23.599510
H	1.844506	2.608225	24.015402
C	5.374629	11.150560	21.514836
H	6.065396	11.473311	22.304541
H	4.734558	11.996453	21.233433
C	1.915947	9.309531	24.330293
C	3.529680	11.485291	23.641061
H	4.145152	12.339800	23.383433
C	7.225646	4.738774	20.144683
H	7.010229	3.995559	19.366214
H	8.035726	4.364730	20.783695
C	5.264072	1.642988	22.303267
H	5.658325	0.632974	22.307013
C	7.762818	9.177638	19.531168
H	8.343170	9.973067	19.038706
H	7.036641	8.776843	18.810664
C	8.725498	8.101248	19.998671
H	9.401289	7.846804	19.169676
H	9.327395	8.488159	20.825627
C	7.637004	6.037236	19.489505
H	8.443991	5.822755	18.774356
H	6.783511	6.464997	18.945728
C	6.133641	10.696373	20.291005
H	5.436020	10.297344	19.542859
H	6.648971	11.569097	19.862149
C	4.976933	6.413618	26.622377
H	4.395434	7.125295	27.216700
H	6.039844	6.582519	26.799373
H	4.699414	5.408217	26.949217
C	0.431462	4.767615	24.268044
C	0.046725	6.091505	24.529049
C	-0.454621	3.722098	24.532151
H	-0.191638	2.695671	24.303387
C	-1.708036	3.999149	25.067966
C	-2.091564	5.316750	25.321091
C	-1.221107	6.365228	25.042725
H	-1.547035	7.386606	25.204582
O	4.709508	7.829798	19.107685
C	3.758489	8.299862	18.470298
O	2.540033	7.969636	18.524535
O	4.083780	9.307928	17.602443
C	3.029430	9.921514	16.869709
H	2.300945	10.384877	17.540892
H	3.503296	10.686824	16.253876
H	2.517491	9.196472	16.231266
H	1.299967	8.302314	21.361182
C	1.034192	7.363237	20.874496
C	2.107501	6.752814	20.099824
O	1.332967	6.214543	21.655446
C	-0.373888	7.366844	20.333347
H	3.125504	6.865321	20.432584
H	1.858504	5.935059	19.437147
H	-0.491249	8.171975	19.601775
H	-0.607568	6.413350	19.850384
H	-1.084178	7.531827	21.149193
H	-3.079096	5.531006	25.715313
H	-2.396032	3.184174	25.265761

4_phen_alkoxy.log

SCF (wB97x) = SCF
E(SCF)+ZPE(0 K)= -3036.375913
H(298 K)= -3036.328674
G(298 K)= -3036.456448
Lowest Frequency = 11.5816cm-1

Co	2.534561	6.208101	23.217021
K	5.769481	7.421400	21.925993
O	3.225973	7.901999	22.648434
O	4.002430	5.452126	22.270353
O	4.636322	9.949604	21.786492
O	3.499868	6.267225	24.928704
O	6.008868	4.917005	20.723244
O	8.186695	6.689210	20.707671
O	7.328499	9.488926	20.935373
O	5.559860	7.081015	24.533836
N	1.839801	4.521290	23.790800
N	1.077542	6.972812	24.184030
C	4.218553	4.203617	22.060057
C	5.328894	3.847228	21.217120
C	2.864878	9.060871	23.075301
C	3.610249	10.215612	22.647515
C	0.927150	8.231367	24.421499
H	0.100663	8.545845	25.058139
C	3.791597	1.789802	22.319850
H	3.186658	0.996390	22.750560
C	4.660814	6.670602	25.278814
C	1.441436	10.607403	24.371998
H	0.593367	10.747867	25.036593
C	5.637284	2.529502	20.960450
H	6.478197	2.274826	20.324996
C	3.450290	3.139794	22.603179
C	2.189786	11.682401	23.966449
H	1.953998	12.686009	24.302125
C	2.305926	3.372154	23.432712
H	1.789669	2.472950	23.767370
C	5.541309	11.007357	21.496316
H	6.041963	11.322610	22.420975
H	5.003566	11.865931	21.074564
C	1.761378	9.293874	23.941074
C	3.283864	11.478391	23.097363
H	3.865182	12.339372	22.789382
C	7.163919	4.684892	19.934680
H	6.901786	4.126765	19.026546
H	7.902132	4.110111	20.508546
C	4.862433	1.485842	21.518624
H	5.122445	0.455203	21.304375
C	8.245846	9.036884	19.957778
H	8.977415	9.826523	19.728785
H	7.708932	8.797540	19.028230
C	8.997671	7.827306	20.480947
H	9.803659	7.584455	19.773380
H	9.448412	8.067277	21.447456
C	7.738730	6.025219	19.539809
H	8.577314	5.854783	18.849775
H	6.972472	6.616375	19.019852
C	6.542713	10.562939	20.458352
H	6.020817	10.264405	19.542811
H	7.182686	11.428492	20.229012
C	4.887218	6.640549	26.785997
H	4.360682	7.488644	27.236573
H	5.949616	6.723460	27.020617
H	4.475659	5.727256	27.222053
C	0.658577	4.663527	24.553994
C	-0.079878	3.613137	25.106471
C	0.223829	5.987037	24.730442
C	-1.254687	3.877894	25.799472
H	0.246866	2.584958	25.005115

C	-0.970963	6.242563	25.408486
C	-1.705985	5.190857	25.941347
H	-1.822642	3.056188	26.222545
H	-1.344249	7.253778	25.521100
H	-2.631790	5.396976	26.467753
O	4.783627	8.107932	18.975726
C	3.789637	8.797653	19.032750
O	2.589670	8.404199	19.408080
C	2.450006	7.052408	19.898179
C	1.233294	7.000500	20.830552
H	3.342547	6.779504	20.454680
H	2.338436	6.387047	19.034609
O	1.390811	5.966376	21.740516
H	1.176037	7.983128	21.328249
C	-0.066090	6.800798	20.051528
H	-0.186315	7.577989	19.287995
H	-0.063388	5.823562	19.555055
H	-0.924382	6.843575	20.728741
O	3.826079	10.080720	18.685568
C	2.695049	10.921149	18.955340
H	2.464097	10.911155	20.023386
H	3.002501	11.918330	18.645105
H	1.825478	10.596948	18.381350

4_phenCl_alkoxy.log

SCF (wB97x) = SCF
E(SCF)+ZPE(0 K)= -3955.603882
H(298 K)= -3955.553931
G(298 K)= -3955.689640
Lowest Frequency = 6.1600cm-1

Co	2.536793	6.209793	23.219772
K	5.774964	7.423499	21.928376
O	3.225378	7.903455	22.651728
O	4.003452	5.451907	22.276261
O	4.637737	9.949856	21.792461
O	3.496368	6.270737	24.931950
O	6.009002	4.917447	20.729480
O	8.184787	6.692069	20.698394
O	7.326210	9.490026	20.928199
O	5.555783	7.084062	24.535448
N	1.841996	4.521410	23.794043
N	1.077128	6.976704	24.183534
C	4.221458	4.204122	22.069668
C	5.332599	3.847879	21.226650
C	2.868783	9.060796	23.084169
C	3.616184	10.215452	22.657778
C	0.931930	8.235944	24.431144
H	0.109007	8.550983	25.072184
C	3.799820	1.787733	22.335677
H	3.196643	0.994088	22.768144
C	4.658514	6.673275	25.281633
C	1.454666	10.608233	24.394213
H	0.609676	10.748801	25.062519
C	5.643240	2.530478	20.974604
H	6.484334	2.275534	20.339578
C	3.454915	3.138940	22.615565
C	2.205161	11.680879	23.990147
H	1.974966	12.684081	24.330644
C	2.310302	3.369777	23.439159
H	1.794006	2.470052	23.772380
C	5.542577	11.007860	21.500237
H	6.047870	11.320016	22.423352
H	5.003056	11.867623	21.083424
C	1.768530	9.294374	23.955725
C	3.295341	11.476328	23.115037
H	3.878228	12.336869	22.808969
C	7.160537	4.685326	19.934940
H	6.893775	4.124662	19.029818
H	7.902489	4.113138	20.506424
C	4.870907	1.485552	21.536097
H	5.134179	0.455337	21.324180
C	8.238438	9.038567	19.945430
H	8.967875	9.828840	19.712015
H	7.696368	8.798223	19.019171
C	8.994199	7.830088	20.465280
H	9.795836	7.586546	19.753085
H	9.450770	8.071344	21.428714
C	7.731261	6.025528	19.534176
H	8.566252	5.854484	18.839992
H	6.961593	6.614821	19.017176
C	6.538626	10.565038	20.456608
H	6.012406	10.268495	19.542929
H	7.177706	11.430894	20.226040
C	4.885768	6.639690	26.788189
H	4.344433	7.475560	27.243848
H	5.946684	6.738884	27.022846
H	4.490030	5.716799	27.218761
C	0.659478	4.667539	24.544857
C	-0.082884	3.621578	25.090582
C	0.223839	5.991603	24.718571
C	-1.263265	3.882144	25.773843
H	0.236595	2.591333	24.999032
C	-0.975090	6.244980	25.381813
C	-1.717440	5.197150	25.909937
H	-1.359300	7.251255	25.491846
O	4.781574	8.106185	18.982420
C	3.785978	8.793941	19.033923
O	2.585580	8.398643	19.406846
C	2.448185	7.047956	19.899710
C	1.232930	6.996520	20.833869
H	3.342286	6.777316	20.454913
H	2.336187	6.380530	19.037882
O	1.393475	5.963620	21.745447
H	1.175346	7.979626	21.330476
C	-0.067710	6.793451	20.058216
H	-0.190612	7.569363	19.293906
H	-0.064482	5.815535	19.563230
H	-0.924491	6.835999	20.737291
O	3.820529	10.075948	18.683577
C	2.685854	10.914227	18.945013
H	2.449277	10.906867	20.011822
H	2.992308	11.911241	18.633372
H	1.820190	10.585888	18.367518
Cl	-3.206358	5.560164	26.730678
Cl	-2.154024	2.544644	26.437134

4_phenF_alkoxy.log

SCF (wB97x) = SCF
E(SCF)+ZPE(0 K)= -3234.868006
H(298 K)= -3234.818860
G(298 K)= -3234.951480
Lowest Frequency = 10.3550cm-1

Co	2.536654	6.209113	23.219268
K	5.773506	7.422730	21.927621
O	3.226455	7.902357	22.651901
O	4.003151	5.451963	22.274948
O	4.637203	9.949195	21.790124
O	3.497451	6.267868	24.931402
O	6.008058	4.917883	20.726779
O	8.185557	6.690466	20.704499
O	7.328283	9.489659	20.933767
O	5.556437	7.082560	24.534920
N	1.841129	4.520710	23.792159
N	1.077790	6.975498	24.185198
C	4.219182	4.203889	22.064908
C	5.329492	3.848082	21.221120
C	2.866156	9.060769	23.079556
C	3.612292	10.215374	22.651696

C	0.927857	8.234856	24.424922
H	0.101646	8.550714	25.061376
C	3.793703	1.788321	22.324957
H	3.189450	0.994535	22.755741
C	4.659036	6.671707	25.281007
C	1.444259	10.608974	24.378776
H	0.596725	10.749971	25.043814
C	5.637976	2.530682	20.964965
H	6.478556	2.276186	20.329063
C	3.451440	3.138924	22.608365
C	2.193599	11.682432	23.973447
H	1.959587	12.686205	24.309698
C	2.307862	3.370033	23.435446
H	1.791933	2.470394	23.769787
C	5.541971	11.007350	21.498864
H	6.044165	11.321668	22.422947
H	5.003235	11.866030	21.078775
C	1.762842	9.294732	23.946410
C	3.287129	11.477500	23.103153
H	3.869041	12.338233	22.795744
C	7.161159	4.686002	19.934643
H	6.896122	4.127670	19.027548
H	7.901002	4.111568	20.506648
C	4.864076	1.485975	21.523781
H	5.125139	0.455739	21.309141
C	8.243701	9.037899	19.954117
H	8.974751	9.827619	19.723880
H	7.704843	8.798739	19.025665
C	8.996506	7.828241	20.475619
H	9.800647	7.584949	19.766147
H	9.449650	8.068122	21.441013
C	7.734508	6.026344	19.537909
H	8.571201	5.855714	18.845666
H	6.966822	6.617304	19.019866
C	6.541466	10.563758	20.458786
H	6.018046	10.265844	19.543927
H	7.180748	11.429681	20.229017
C	4.886079	6.640559	26.787746
H	4.353444	7.483722	27.240310
H	5.947951	6.730413	27.021964
H	4.481266	5.723337	27.221883
C	0.661014	4.666080	24.548538
C	-0.078061	3.611752	25.092815
C	0.226782	5.990084	24.725758
C	-1.244754	3.887599	25.776062
H	0.223550	2.575652	25.003016
C	-0.967943	6.250975	25.402124
C	-1.691515	5.197692	25.922689
H	-1.362052	7.251467	25.530561
O	4.784340	8.106689	18.981756
C	3.789393	8.795310	19.034525
O	2.588975	8.400969	19.408176
C	2.450327	7.049837	19.899654
C	1.234199	6.998394	20.832740
H	3.343609	6.778017	20.455549
H	2.338629	6.383341	19.037044
O	1.393280	5.965380	21.744252
H	1.176297	7.981509	21.329294
C	-0.065662	6.795970	20.055497
H	-0.187441	7.572101	19.291212
H	-0.062210	5.818169	19.560249
H	-0.923241	6.838641	20.733571
O	3.824875	10.077506	18.684767
C	2.691253	10.916680	18.947834
H	2.455773	10.908870	20.014892
H	2.998333	11.913608	18.636504
H	1.824701	10.589538	18.370980
F	-2.836269	5.426340	26.573988
F	-1.966050	2.891869	26.300382

4_phenMe_alkoxy.log

SCF (wB97x) = SCF
E(SCF)+ZPE(0 K)= -3114.939982
H(298 K)= -3114.889405
G(298 K)= -3115.023970
Lowest Frequency = 13.6673cm-1

Co	2.573322	6.139901	23.281856
K	5.763389	7.396735	21.931516
O	3.237978	7.847845	22.723933
O	4.022618	5.410459	22.287129
O	4.609869	9.919010	21.857178
O	3.581385	6.177882	24.970460
O	5.986353	4.917866	20.673174
O	8.154219	6.701188	20.640738
O	7.286373	9.490867	20.939249
O	5.624287	7.016424	24.539451
N	1.906089	4.437169	23.844265
N	1.135696	6.878818	24.298794
C	4.238296	4.166987	22.044748
C	5.325963	3.834137	21.164113
C	2.874980	8.998277	23.172868
C	3.600051	10.164889	22.743592
C	0.976346	8.132099	24.552926
H	0.161381	8.430114	25.212147
C	3.831668	1.747022	22.267786
H	3.243191	0.941716	22.699279
C	4.747820	6.583144	25.298503
C	1.465297	10.514662	24.517406
H	0.630431	10.638886	25.201721
C	5.634480	2.523495	20.871901
H	6.458531	2.286796	20.207985
C	3.491422	3.088418	22.587505
C	2.194468	11.602336	24.109036
H	1.955935	12.599426	24.461862
C	2.369090	3.298053	23.454966
H	1.869151	2.389750	23.790474
C	5.500584	10.987486	21.563024
H	6.018900	11.293411	22.481084
H	4.947826	11.848035	21.165310
C	1.788492	9.209977	24.064590
C	3.271576	11.419563	23.215051
H	3.838059	12.289685	22.905081
C	7.121570	4.708775	19.850443
H	6.838919	4.169027	18.937332
H	7.877125	4.125609	20.392422
C	4.881235	1.464861	21.430334
H	5.140103	0.440073	21.188307
C	8.186132	9.062960	19.934814
H	8.908725	9.861000	19.706471
H	7.631944	8.838660	19.011671
C	8.954654	7.847559	20.418729
H	9.746549	7.622524	19.689648
H	9.424772	8.071246	21.379901
C	7.679688	6.060435	19.470344
H	8.501113	5.909805	18.755497
H	6.897392	6.658595	18.983314
C	6.482122	10.566078	20.496857
H	5.942016	10.277306	19.588834
H	7.110449	11.439851	20.266421
C	5.014467	6.524963	26.798411
H	4.510441	7.371683	27.276613
H	6.083768	6.593347	27.005028
H	4.604854	5.609311	27.231157
C	0.747148	4.562646	24.644093
C	0.026837	3.503634	25.202300
C	0.310016	5.874265	24.854657
C	-1.131569	3.725623	25.940259
H	0.360583	2.480143	25.069846
C	-0.864683	6.100422	25.575675
C	-1.592077	5.047461	26.121178

H -1.238042 7.109218 25.716420
O 4.701236 8.124629 19.014725
C 3.704954 8.806893 19.109083
O 2.517725 8.399657 19.509273
C 2.398977 7.038592 19.979118
C 1.207277 6.961856 20.941508
H 3.307092 6.761766 20.507744
H 2.269314 6.387768 19.106993
O 1.396095 5.913592 21.828860
H 1.155976 7.935492 21.457160
C -0.110571 6.765818 20.193012
H -0.256447 7.555143 19.446557
H -0.113875 5.797324 19.679634
H -0.951082 6.790043 20.893133
O 3.724546 10.095580 18.781619
C 2.597178 10.924805 19.097354
H 2.397975 10.896840 20.171503
H 2.889668 11.928540 18.793824
H 1.712944 10.604218 18.544125
C -2.855157 5.319710 26.895797
H -3.715983 4.819667 26.438606
H -2.777543 4.947618 27.923242
H -3.068881 6.390011 26.939004
C -1.887946 2.564014 26.530334
H -1.956897 2.649552 27.620325
H -2.913435 2.523270 26.147233
H -1.400887 1.615005 26.295320

4_phenOMe_alkoxy.log

SCF (wB97x) = SCF
E(SCF)+ZPE(0 K)= -3265.334150
H(298 K)= -3265.281453
G(298 K)= -3265.421274
Lowest Frequency = 12.6188cm-1

Co 2.576342 6.147051 23.287199
K 5.766962 7.396636 21.929267
O 3.241752 7.853590 22.726487
O 4.022008 5.414424 22.292268
O 4.617359 9.921209 21.857806
O 3.586099 6.187155 24.974364
O 5.980499 4.915988 20.674101
O 8.151188 6.695967 20.629042
O 7.289812 9.487282 20.929276
O 5.628415 7.024478 24.538542
N 1.908012 4.446523 23.854052
N 1.140024 6.889814 24.306533
C 4.236501 4.169467 22.053076
C 5.321207 3.833927 21.170368
C 2.884964 9.004016 23.179198
C 3.612588 10.169037 22.749005
C 0.988445 8.143778 24.565220
H 0.179016 8.445472 25.229638
C 3.830412 1.750550 22.285040
H 3.243124 0.946648 22.720729
C 4.753617 6.591921 25.299745
C 1.487575 10.523443 24.534449
H 0.656475 10.649522 25.222975
C 5.628820 2.522232 20.881011
H 6.450693 2.283526 20.215143
C 3.491330 3.092780 22.601378
C 2.219080 11.608664 24.125036
H 1.986508 12.605936 24.481241
C 2.370184 3.305509 23.470242
H 1.870495 2.398769 23.810723
C 5.510197 10.987517 21.561408
H 6.033121 11.290530 22.477797
H 4.958219 11.850119 21.167139
C 1.803268 9.218142 24.076735
C 3.291271 11.423534 23.225178
H 3.859757 12.292183 22.914801
C 7.112051 4.703800 19.847092
H 6.824908 4.162406 18.936370
H 7.869252 4.120976 20.387120
C 4.877473 1.465620 21.445116
H 5.135335 0.440015 21.205481
C 8.183131 9.056725 19.920169
H 8.905700 9.853332 19.686815
H 7.623184 8.832206 19.000573
C 8.952346 7.840693 20.401314
H 9.739886 7.613447 19.668220
H 9.428062 8.064795 21.359634
C 7.670249 6.053955 19.461909
H 8.488172 5.900889 18.743584
H 6.886601 6.652456 18.977483
C 6.486212 10.564553 20.490872
H 5.941645 10.277670 19.584962
H 7.115558 11.437040 20.258359
C 5.023330 6.533438 26.799028
H 4.515162 7.376358 27.279509
H 6.092599 6.607434 27.003775
H 4.619757 5.614712 27.230998
C 0.751056 4.577918 24.652118
C 0.030052 3.518585 25.215202
C 0.312991 5.887754 24.860785
C -1.120981 3.756370 25.942682
H 0.338785 2.485675 25.105012
C -0.866006 6.134773 25.574889
C -1.591198 5.079075 26.113897
H -1.230571 7.146077 25.693789
O 4.699527 8.124259 19.016131
C 3.704142 8.807749 19.110979
O 2.516991 8.402394 19.513347
C 2.396973 7.041856 19.984241
C 1.207104 6.968004 20.949092
H 3.305750 6.763714 20.511085
H 2.264387 6.390691 19.112839
O 1.396048 5.920394 21.837311
H 1.158396 7.942287 21.463821
C -0.112656 6.773035 20.203768
H -0.258931 7.561749 19.456762
H -0.118534 5.803982 19.691525
H -0.951573 6.799314 20.905714
O 3.724728 10.096040 18.782109
C 2.599001 10.927045 19.099047
H 2.401126 10.899705 20.173446
H 2.892606 11.930245 18.794844
H 1.713597 10.607573 18.547045
O -2.745165 5.213930 26.798250
O -1.828125 2.699204 26.435220
C -3.253649 6.518810 27.025021
H -3.488683 7.021139 26.080103
H -4.168144 6.384763 27.600793
H -2.544213 7.123490 27.600964
C -1.740906 2.525871 27.850123
H -2.135667 3.399649 28.377114
H -2.343111 1.649394 28.089680
H -0.700660 2.349152 28.146511

9_phen_carbonate.log

SCF (wB97x) = SCF
E(SCF)+ZPE(0 K)= -3224.949277
H(298 K)= -3224.898516
G(298 K)= -3225.039994
Lowest Frequency = 3.7270cm-1

Co 2.747889 6.169157 23.406496
K 5.877718 7.424927 21.922420
O 3.375904 7.856497 22.826598
O 4.152230 5.394973 22.374787

O	4.624600	9.900763	21.849549
O	3.781718	6.155225	25.024335
O	6.284115	4.840836	20.997436
O	8.207707	6.841295	20.429857
O	7.026646	9.514137	20.431744
O	5.812709	6.980910	24.508886
N	2.065353	4.476427	23.985283
N	1.350432	6.903106	24.469843
C	4.568081	4.178946	22.459382
C	5.752352	3.820465	21.725541
C	3.107550	8.985068	23.385386
C	3.799121	10.149012	22.903455
C	1.313584	8.125630	24.880158
H	0.557116	8.403095	25.614708
C	4.495314	1.841421	23.248084
H	3.991052	1.076700	23.832458
C	4.984470	6.514943	25.294590
C	1.994546	10.464277	25.013702
H	1.273531	10.579898	25.818342
C	6.271938	2.545415	21.785485
H	7.172684	2.292557	21.237715
C	3.939525	3.147973	23.209657
C	2.710240	11.544336	24.562891
H	2.580620	12.524071	25.008921
C	2.699506	3.356361	23.894973
H	2.265160	2.473997	24.365826
C	5.339576	10.979742	21.272468
H	6.057946	11.387304	21.995493
H	4.649945	11.776860	20.966696
C	2.172154	9.179565	24.435956
C	3.616541	11.382619	23.489650
H	4.165339	12.247220	23.133204
C	7.454614	4.589892	20.236942
H	7.270532	3.789974	19.508510
H	8.278066	4.290788	20.897912
C	5.642677	1.543333	22.558929
H	6.069506	0.547210	22.591403
C	7.771861	9.005056	19.342911
H	8.311606	9.818826	18.836081
H	7.097883	8.540682	18.607433
C	8.786677	8.005648	19.867142
H	9.474086	7.734987	19.053806
H	9.366992	8.471161	20.668202
C	7.821636	5.852789	19.494215
H	8.655427	5.624028	18.815637
H	6.968023	6.188675	18.889068
C	6.056508	10.465874	20.046610
H	5.333065	10.013049	19.352843
H	6.533874	11.317025	19.539937
C	5.341238	6.317586	26.760876
H	4.695045	6.945752	27.381135
H	6.384452	6.579261	26.941831
H	5.164866	5.278237	27.051019
C	0.806066	4.602840	24.613099
C	-0.049561	3.548288	24.934129
C	0.417322	5.925613	24.874499
C	-1.277707	3.815342	25.530496
H	0.217184	2.523358	24.702420
C	-0.826923	6.188480	25.449107
C	-1.665733	5.131302	25.786251
H	-1.159450	7.207576	25.611700
O	1.613913	5.981260	21.863768
C	0.758395	6.816642	21.420575
O	0.458874	7.926119	21.836135
O	0.140260	6.290256	20.313489
C	-0.926875	7.040257	19.734347
H	-0.689761	8.106959	19.789596
C	-0.932706	6.601383	18.276382
C	-2.237358	6.754896	20.451938
H	-1.192314	5.542216	18.184600
H	0.046343	6.772705	17.821296
O	-1.919740	7.398318	17.608993
H	-2.467720	5.685215	20.411598
H	-2.166371	7.063569	21.497721
H	-3.055204	7.307731	19.982209
C	-2.122250	7.134855	16.324844
O	-1.548956	6.290620	15.678306
O	-3.060137	7.955552	15.872970
C	-3.411291	7.812269	14.490944
H	-3.801226	6.810558	14.300013
H	-4.181459	8.559542	14.311589
H	-2.542882	8.001045	13.856761
H	-2.635110	5.337325	26.227301
H	-1.943665	2.994199	25.773116

9_phenCl_carbonate.log

SCF (wB97x) = SCF
E(SCF)+ZPE(0 K)= -4144.176915
H(298 K)= -4144.123631
G(298 K)= -4144.271029
Lowest Frequency = 2.8147cm-1

Co	2.598075	6.246203	23.127498
K	5.894139	7.441132	21.986165
O	3.326417	7.928279	22.667085
O	4.067442	5.455554	22.210631
O	4.727978	9.956864	21.889480
O	3.468108	6.155025	24.834111
O	6.301292	4.873422	21.022965
O	8.327207	6.826337	20.690623
O	7.231932	9.534757	20.676376
O	5.563341	6.925824	24.538751
N	1.814766	4.558880	23.586231
N	1.123118	6.994507	24.071584
C	4.441344	4.227393	22.304300
C	5.679828	3.852828	21.674483
C	3.038743	9.045709	23.236220
C	3.810914	10.200333	22.864795
C	1.078978	8.206926	24.516499
H	0.260732	8.487755	25.180155
C	4.237451	1.874336	23.027203
H	3.662216	1.110722	23.543199
C	4.649505	6.470211	25.229158
C	1.815312	10.513109	24.798317
H	1.022222	10.628441	25.531933
C	6.159605	2.563977	21.753656
H	7.101270	2.298690	21.286605
C	3.718937	3.196668	22.967723
C	2.606000	11.579650	24.457471
H	2.464349	12.549172	24.921593
C	2.425304	3.421308	23.531636
H	1.924032	2.540599	23.934399
C	5.524571	11.030112	21.416949
H	6.186857	11.389050	22.215236
H	4.889256	11.859143	21.080322
C	2.009090	9.240268	24.196524
C	3.609662	11.419401	23.473178
H	4.218463	12.274955	23.202799
C	7.528879	4.606763	20.363716
H	7.388691	3.831792	19.599451
H	8.280075	4.265818	21.087311
C	5.436404	1.562129	22.442024
H	5.834756	0.555005	22.490610
C	8.054065	9.034300	19.640065
H	8.660512	9.844621	19.208964
H	7.433313	8.614445	18.834344
C	8.988408	7.986923	20.217515
H	9.735884	7.717603	19.458527
H	9.510289	8.409378	21.080252
C	7.997230	5.877250	19.694630
H	8.881053	5.640537	19.085748

H 7.211285 6.257338 19.027087
C 6.332264 10.533170 20.241655
H 5.659976 10.130444 19.469964
H 6.880690 11.383602 19.811542
C 4.854908 6.226748 26.717008
H 4.149118 6.836107 27.288915
H 5.874770 6.477735 27.010438
H 4.650506 5.178844 26.953492
C 0.503570 4.711102 24.075375
C -0.413567 3.681169 24.258986
C 0.127628 6.038221 24.336195
C -1.694648 3.967423 24.717894
H -0.162418 2.653353 24.027648
C -1.163354 6.324021 24.769317
C -2.070509 5.289918 24.971307
H -1.491454 7.344028 24.927855
O 1.599734 6.130848 21.487985
C 0.775917 6.985877 21.019794
O 0.510003 8.111397 21.414362
O 0.155689 6.461556 19.914794
C -0.893857 7.226305 19.322046
H -0.632800 8.288168 19.359726
C -0.908119 6.762377 17.872031
C -2.209623 6.980928 20.045013
H -1.190043 5.707473 17.798753
H 0.074921 6.904580 17.415547
O -1.877175 7.567868 17.189157
H -2.463175 5.916012 20.021771
H -2.131056 7.304160 21.085967
H -3.015763 7.544031 19.567369
C -2.080019 7.287422 15.908479
O -1.520267 6.422053 15.278266
O -3.000405 8.118049 15.439451
C -3.348996 7.958149 14.058513
H -3.757722 6.961115 13.883067
H -4.103650 8.717159 13.863287
H -2.474552 8.118959 13.424930
Cl -3.676658 5.686738 25.503252
Cl -2.814013 2.654476 24.927891

9_phenF_carbonate_t1.log

SCF (wB97x) = SCF
E(SCF)+ZPE(0 K)= -3423.440939
H(298 K)= -3423.388342
G(298 K)= -3423.534287
Lowest Frequency = 2.4793cm-1

Co 2.761014 6.177138 23.386079
K 5.915278 7.428870 21.944095
O 3.400315 7.863222 22.820834
O 4.174213 5.401816 22.371246
O 4.661876 9.905392 21.859878
O 3.771506 6.154327 25.016722
O 6.316731 4.845447 21.013686
O 8.249569 6.841469 20.464492
O 7.074043 9.516549 20.460842
O 5.809503 6.977967 24.527738
N 2.065257 4.483108 23.949512
N 1.350939 6.913180 24.433456
C 4.583355 4.183908 22.454719
C 5.774205 3.824254 21.731621
C 3.125042 8.991203 23.376207
C 3.823978 10.154681 22.902949
C 1.309764 8.136356 24.845541
H 0.544057 8.414759 25.570211
C 4.493537 1.841702 23.232081
H 3.980230 1.076123 23.807216
C 4.972004 6.511591 25.302692
C 1.991954 10.472506 24.990767
H 1.260631 10.589032 25.785797
C 6.288158 2.547364 21.792302
H 7.193963 2.293500 21.253482
C 3.942396 3.151100 23.193215
C 2.714892 11.550669 24.549275
H 2.581112 12.530680 24.993335
C 2.696310 3.359606 23.862805
H 2.253384 2.476464 24.324358
C 5.382606 10.984273 21.289020
H 6.095252 11.390662 22.018266
H 4.695989 11.781896 20.978035
C 2.175563 9.186889 24.414793
C 3.635142 11.387682 23.487445
H 4.189590 12.251740 23.138590
C 7.492286 4.592562 20.261074
H 7.310834 3.794789 19.529719
H 8.309751 4.289625 20.927622
C 5.647020 1.543617 22.554647
H 6.070522 0.546133 22.587151
C 7.827834 9.008024 19.377584
H 8.373657 9.821669 18.877161
H 7.159374 8.546510 18.635332
C 8.835953 8.005543 19.908740
H 9.529682 7.734711 19.100863
H 9.410424 8.468304 20.715569
C 7.868288 5.855949 19.523792
H 8.706725 5.626275 18.851306
H 7.020279 6.195246 18.912728
C 6.108738 10.470298 20.068693
H 5.390219 10.019399 19.368607
H 6.591591 11.321186 19.566839
C 5.309670 6.311485 26.772786
H 4.667085 6.952197 27.383984
H 6.354766 6.557677 26.964114
H 5.113333 5.276145 27.064055
C 0.801009 4.614444 24.556998
C -0.059474 3.558075 24.858889
C 0.413136 5.937940 24.818393
C -1.284495 3.838422 25.433325
H 0.182333 2.525540 24.637748
C -0.837300 6.208407 25.376022
C -1.670361 5.151443 25.688644
H -1.192591 7.217252 25.548173
O 1.643066 5.995420 21.831600
C 0.779238 6.827169 21.395759
O 0.486306 7.938888 21.809829
O 0.144952 6.293671 20.302596
C -0.938650 7.035146 19.742522
H -0.705257 8.103217 19.785308
C -0.975846 6.586714 18.288001
C -2.230814 6.748186 20.492023
H -1.234305 5.526243 18.208659
H -0.007870 6.758147 17.809829
O -1.980165 7.376798 17.638811
H -2.456521 5.677141 20.464111
H -2.137892 7.064417 21.533853
H -3.061909 7.293661 20.037218
C -2.211278 7.105415 16.361085
O -1.650376 6.259163 15.706392
O -3.161606 7.920974 15.926402
C -3.543733 7.769647 14.553469
H -3.935956 6.766236 14.376710
H -4.319260 8.514509 14.387710
H -2.690334 7.956697 13.898743
F -2.876859 5.382495 26.214600
F -2.130977 2.845476 25.722706

9_phenMe_carbonate.log

SCF (wB97x) = SCF
E(SCF)+ZPE(0 K)= -3303.513908
H(298 K)= -3303.459916

G(298 K)= -3303.606651
Lowest Frequency = 7.7936cm-1

Co	2.853205	6.396835	23.033000
K	6.234423	7.500964	22.102072
O	3.664028	8.074666	22.695573
O	4.325691	5.589456	22.134855
O	5.207598	10.073140	22.127440
O	3.643334	6.177196	24.764963
O	6.600221	4.966544	21.046595
O	8.719768	6.845415	20.914498
O	7.747203	9.598699	21.006624
O	5.781009	6.868254	24.604600
N	1.974013	4.728129	23.358653
N	1.373099	7.158998	23.959167
C	4.647984	4.342135	22.194746
C	5.903482	3.944900	21.617231
C	3.415600	9.165103	23.336757
C	4.261972	10.297749	23.080682
C	1.361386	8.339820	24.475032
H	0.519229	8.620524	25.108345
C	4.311981	1.972733	22.794285
H	3.678370	1.211377	23.240949
C	4.818199	6.414652	25.226621
C	2.203209	10.582893	24.947788
H	1.385773	10.690180	25.655616
C	6.329474	2.634480	21.662102
H	7.285219	2.352250	21.235184
C	3.850850	3.314990	22.769063
C	3.063029	11.627311	24.717261
H	2.950541	12.567948	25.244673
C	2.531226	3.569598	23.269755
H	1.961610	2.693769	23.582727
C	6.080196	11.129764	21.765796
H	6.720762	11.399261	22.615418
H	5.505778	12.012784	21.458032
C	2.357655	9.347491	24.266165
C	4.098534	11.482238	23.766123
H	4.762240	12.319618	23.581521
C	7.849877	4.679223	20.440936
H	7.721785	3.946115	19.634237
H	8.547534	4.274977	21.185371
C	5.530717	1.635480	22.262285
H	5.885048	0.611058	22.286457
C	8.587267	9.115774	19.976354
H	9.245696	9.918749	19.612957
H	7.981690	8.767344	19.126218
C	9.450386	7.997594	20.531713
H	10.216143	7.734674	19.788912
H	9.954161	8.349899	21.435773
C	8.403535	5.958338	19.858535
H	9.307472	5.712684	19.283657
H	7.669321	6.402365	19.171787
C	6.917994	10.666659	20.597469
H	6.264394	10.347366	19.772508
H	7.526705	11.512877	20.247528
C	4.945453	6.076628	26.704975
H	4.251996	6.695264	27.282131
H	5.964221	6.249982	27.053494
H	4.670633	5.031627	26.872708
C	0.634810	4.920825	23.764789
C	-0.355791	3.940032	23.793132
C	0.312095	6.239114	24.098559
C	-1.661679	4.253260	24.164220
H	-0.128143	2.922426	23.491973
C	-0.994799	6.561212	24.460526
C	-1.985830	5.583861	24.503602
H	-1.265996	7.591184	24.669413
O	1.920946	6.400368	21.336800
C	0.897030	7.090207	21.015746
O	0.590261	8.227535	21.343476
O	0.098695	6.365603	20.166324
C	-1.168761	6.917661	19.812596
H	-1.064534	7.995758	19.656303
C	-1.502536	6.245075	18.488172
C	-2.193894	6.637930	20.901725
H	-1.638306	5.167098	18.618603
H	-0.709239	6.422160	17.757047
O	-2.724775	6.831505	18.022469
H	-2.266565	5.560911	21.088182
H	-1.899543	7.131749	21.830498
H	-3.177171	7.011704	20.603966
C	-3.248950	6.309912	16.921310
O	-2.785354	5.394888	16.283179
O	-4.367960	6.963327	16.641488
C	-5.077336	6.528304	15.474634
H	-5.386834	5.487089	15.584793
H	-5.947809	7.177709	15.409229
H	-4.452163	6.638881	14.586473
C	-3.400920	5.960159	24.855208
H	-4.079759	5.757455	24.019035
H	-3.765697	5.383290	25.711803
H	-3.476975	7.021170	25.102814
C	-2.724151	3.185597	24.161404
H	-3.179511	3.076687	25.151658
H	-3.531157	3.434408	23.463267
H	-2.310505	2.217683	23.870087

9_phenOMe_carbonate.log

SCF (wB97x) = SCF
E(SCF)+ZPE(0 K)= -3453.905056
H(298 K)= -3453.848670
G(298 K)= -3454.001382
Lowest Frequency = 8.1597cm-1

Co	2.850697	6.402147	23.029114
K	6.239710	7.501523	22.112484
O	3.665828	8.077697	22.696065
O	4.325256	5.593964	22.136960
O	5.211541	10.073911	22.127595
O	3.632094	6.177444	24.763494
O	6.598687	4.968518	21.049062
O	8.723223	6.841565	20.928393
O	7.755108	9.596140	21.017295
O	5.769441	6.871360	24.612995
N	1.966451	4.734758	23.346873
N	1.368255	7.166070	23.952504
C	4.640912	4.344961	22.187985
C	5.896208	3.946171	21.610615
C	3.413799	9.170086	23.331416
C	4.261388	10.302083	23.075022
C	1.355207	8.349815	24.463935
H	0.512061	8.633482	25.094351
C	4.292739	1.972111	22.769955
H	3.654729	1.210546	23.209812
C	4.805283	6.414855	25.230367
C	2.192686	10.594415	24.931256
H	1.371862	10.704253	25.634687
C	6.315817	2.633700	21.647581
H	7.271447	2.350074	21.221365
C	3.837507	3.317025	22.752874
C	3.053229	11.637544	24.700605
H	2.938110	12.580370	25.223455
C	2.519551	3.573811	23.252844
H	1.946488	2.698981	23.562435
C	6.085673	11.129678	21.766340
H	6.722659	11.401452	22.617890
H	5.512284	12.011659	21.453834
C	2.350957	9.355846	24.255037
C	4.093841	11.488768	23.755058
H	4.758111	12.325727	23.570698

C 7.848646 4.679598 20.444372
H 7.719473 3.950535 19.634233
H 8.543301 4.269720 21.188509
C 5.510990 1.633712 22.238692
H 5.861020 0.607710 22.256425
C 8.601926 9.113490 19.992408
H 9.264474 9.915902 19.635346
H 8.002047 8.767789 19.137163
C 9.458864 7.992696 20.552037
H 10.228974 7.729088 19.813988
H 9.957309 8.342502 21.459996
C 8.407519 5.959270 19.868323
H 9.312328 5.713126 19.295041
H 7.676411 6.407761 19.181185
C 6.928098 10.663647 20.602582
H 6.277915 10.343043 19.775448
H 7.538571 11.509006 20.253667
C 4.927133 6.072208 26.707943
H 4.228987 6.686289 27.284257
H 5.943815 6.247809 27.061343
H 4.655308 5.025637 26.870661
C 0.630122 4.927505 23.755966
C -0.357702 3.944125 23.776401
C 0.308479 6.247650 24.096127
C -1.654035 4.266132 24.158568
H -0.161194 2.925871 23.459865
C -0.990733 6.571230 24.483233
C -1.969626 5.586571 24.532661
H -1.283417 7.587868 24.719827
O 1.928518 6.410333 21.328323
C 0.903388 7.097886 21.005483
O 0.588571 8.230592 21.341203
O 0.114791 6.376263 20.145108
C -1.158216 6.919655 19.797876
H -1.063860 7.999301 19.646211
C -1.491318 6.249118 18.472322
C -2.176319 6.624863 20.889522
H -1.615117 5.169330 18.599450
H -0.703487 6.437143 17.737997
O -2.721656 6.824747 18.015061
H -2.235835 5.546102 21.069131
H -1.883963 7.118047 21.819313
H -3.165188 6.988504 20.598159
C -3.253150 6.293042 16.922177
O -2.789613 5.377354 16.284983
O -4.379166 6.937280 16.649637
C -5.096759 6.490951 15.492097
H -5.397327 5.448055 15.610620
H -5.972643 7.133549 15.431989
H -4.481160 6.601606 14.597278
O -3.242570 5.949005 24.858475
O -2.602778 3.285264 24.165145
C -3.737257 5.413785 26.088263
H -4.744931 5.811072 26.208406
H -3.770020 4.321064 26.056053
H -3.109345 5.744146 26.923010
C -3.561592 3.374272 23.108588
H -3.065516 3.261652 22.137974
H -4.260945 2.552182 23.259517
H -4.096895 4.328105 23.144635

CO2.log

SCF (wB97x) = SCF
E(SCF)+ZPE(0 K)= -188.562314
H(298 K)= -188.558737
G(298 K)= -188.583663
Lowest Frequency = 654.2543cm-1

C -4.081649 0.326224 -0.049161
O -2.923431 0.326224 -0.049161
O -5.239866 0.326224 -0.049161

CO2_phenCl_TS_2.log

SCF (wB97x) = SCF
E(SCF)+ZPE(0 K)= -4144.160774
H(298 K)= -4144.107931
G(298 K)= -4144.250143
Lowest Frequency = -226.5899cm-1

Co 2.450761 6.306323 23.305266
K 5.585608 7.558836 21.928078
O 3.139446 8.017165 22.866277
O 3.926023 5.544070 22.384544
O 4.441592 10.074281 21.990146
O 3.363876 6.219560 24.989782
O 6.018639 5.007925 20.945272
O 7.985102 6.981787 20.533404
O 6.866586 9.693377 20.625430
O 5.410786 7.059759 24.561397
N 1.696913 4.604763 23.771296
N 0.995660 7.036888 24.321176
C 4.219603 4.302027 22.280463
C 5.391080 3.947296 21.518457
C 2.818368 9.141875 23.398564
C 3.536590 10.312998 22.974007
C 0.942662 8.255404 24.760698
H 0.174639 8.510243 25.490506
C 3.926923 1.888052 22.727902
H 3.345448 1.096416 23.192070
C 4.558632 6.578646 25.309116
C 1.561095 10.606839 24.936570
H 0.779480 10.712035 25.683539
C 5.799182 2.637828 21.411707
H 6.693498 2.385378 20.853237
C 3.481045 3.233884 22.862249
C 2.299644 11.691500 24.544669
H 2.126770 12.669217 24.979940
C 2.268545 3.457835 23.571403
H 1.790084 2.564135 23.970399
C 5.168097 11.162662 21.444665
H 5.861439 11.566098 22.193629
H 4.483533 11.958538 21.125271
C 1.796774 9.324231 24.367407
C 3.292929 11.540901 23.546793
H 3.860859 12.411772 23.239015
C 7.173909 4.770109 20.157472
H 6.950529 4.049566 19.360442
H 7.981390 4.372471 20.785149
C 5.064071 1.593714 22.026029
H 5.408076 0.570530 21.925809
C 7.649435 9.214866 19.548922
H 8.232792 10.036459 19.107377
H 6.998878 8.805757 18.761431
C 8.613819 8.162982 20.066712
H 9.335556 7.921982 19.273836
H 9.162751 8.568759 20.920520
C 7.594055 6.074433 19.521423
H 8.435372 5.871220 18.843803
H 6.763360 6.477047 18.925092
C 5.919181 10.664701 20.232573
H 5.212455 10.234288 19.507995
H 6.420223 11.521290 19.758974
C 4.857196 6.368030 26.786352
H 4.268547 7.078617 27.375030
H 5.916995 6.527062 26.989169
H 4.563757 5.361700 27.095304
C 0.473766 4.729148 24.462329
C 0.089529 6.047271 24.749014
C -0.366465 3.674668 24.813047
H -0.115759 2.647654 24.580044

C	-1.570880	3.927345	25.457356
C	-1.956055	5.242479	25.737321
C	-1.131122	6.298269	25.371347
H	-1.472703	7.308745	25.557990
O	-2.979175	7.883395	22.962382
C	-2.971899	6.716960	22.648411
O	-2.008389	6.100387	21.981141
C	-0.844322	6.873003	21.617913
C	0.266671	5.854404	21.322318
H	-0.568495	7.523410	22.446391
H	-1.095966	7.490291	20.752926
O	1.547882	6.365794	21.586664
H	0.088414	5.009549	21.996742
C	0.187229	5.322084	19.894229
H	-0.800314	4.887724	19.708555
H	0.346974	6.124745	19.166090
H	0.944966	4.549860	19.737835
O	-3.974630	5.907112	22.966566
C	-3.918948	4.518246	22.608188
H	-3.025633	4.049323	23.025280
H	-4.814123	4.077810	23.042960
H	-3.926780	4.402514	21.522903
C	2.100466	7.712196	20.447358
O	3.122992	7.337107	19.972574
O	1.252939	8.549156	20.487537
Cl	-3.462927	5.595004	26.522697
Cl	-2.589398	2.582499	25.871731

CO2_phenF_TS.log

SCF (wB97x) = SCF
E(SCF)+ZPE(0 K)= -3423.424184
H(298 K)= -3423.372081
G(298 K)= -3423.511235
Lowest Frequency = -226.3188cm-1

Co	2.476093	6.197603	23.326625
K	5.650672	7.540755	22.165780
O	3.120049	7.917896	22.864674
O	4.043371	5.473116	22.538088
O	4.333723	9.987306	21.897758
O	3.266371	6.185427	25.076271
O	6.116808	5.062692	21.015964
O	8.107155	7.016825	20.872514
O	6.930509	9.690104	20.861458
O	5.284418	7.146085	24.794242
N	1.756712	4.474054	23.741604
N	0.928999	6.880226	24.241654
C	4.192803	4.273915	22.110618
C	5.343202	3.981016	21.293808
C	2.622064	9.053675	23.199544
C	3.279686	10.238619	22.718138
C	0.713686	8.131294	24.501061
H	-0.126206	8.393354	25.141255
C	3.598540	1.880438	21.923472
H	2.913593	1.074720	22.172448
C	4.402675	6.630166	25.483150
C	1.033941	10.541212	24.362917
H	0.154347	10.645987	24.991831
C	5.593893	2.700311	20.854586
H	6.474498	2.486784	20.259386
C	3.321003	3.189486	22.406868
C	1.706537	11.647595	23.917198
H	1.379221	12.644919	24.188508
C	2.175442	3.356850	23.235611
H	1.613528	2.448066	23.449729
C	5.040721	11.083932	21.343694
H	5.592879	11.610563	22.132570
H	4.346515	11.785909	20.864904
C	1.470712	9.233965	24.010114
C	2.839975	11.491195	23.083011
H	3.357766	12.378190	22.735896
C	7.245668	4.898612	20.174119
H	6.975673	4.336017	19.271802
H	8.036680	4.358437	20.709416
C	4.712950	1.636468	21.166723
H	4.932506	0.639158	20.802550
C	7.939768	9.299248	19.951287
H	8.584204	10.156135	19.705115
H	7.486463	8.947167	19.013118
C	8.799016	8.218491	20.582984
H	9.650069	8.015792	19.916744
H	9.186883	8.573900	21.540888
C	7.719146	6.266185	19.739633
H	8.568076	6.136548	19.052971
H	6.909356	6.768559	19.192470
C	5.981933	10.563465	20.283949
H	5.413074	10.037462	19.503253
H	6.483513	11.426228	19.822210
C	4.587102	6.480612	26.986743
H	3.919042	7.180071	27.499227
H	5.617386	6.698863	27.270461
H	4.316046	5.471686	27.307277
C	0.621693	4.550658	24.574454
C	0.154338	5.851797	24.818049
C	-0.037611	3.452537	25.132022
H	0.312061	2.435082	25.008216
C	-1.173931	3.663428	25.886984
C	-1.656808	4.951381	26.101197
C	-1.001617	6.047767	25.578164
H	-1.419157	7.028516	25.769441
O	-2.188995	8.309213	23.299475
C	-2.485733	7.185932	22.964936
O	-1.854872	6.445659	22.068424
C	-0.651282	6.966181	21.455791
C	0.328495	5.783381	21.341726
H	-0.242020	7.759810	22.076017
H	-0.935603	7.380595	20.487032
O	1.667791	6.150744	21.564586
H	0.031905	5.081989	22.129208
C	0.200717	5.054027	20.007934
H	-0.832299	4.725898	19.852490
H	0.478163	5.709801	19.175632
H	0.853076	4.176633	19.997207
O	-3.522778	6.558748	23.508194
C	-3.792558	5.190513	23.166712
H	-2.931985	4.561236	23.404636
H	-4.646121	4.909366	23.780460
H	-4.039164	5.099640	22.107741
C	2.360203	7.346409	20.330844
O	3.421616	6.913696	20.023602
O	1.525386	8.182706	20.175090
F	-2.765752	5.118098	26.826332
F	-1.822396	2.627645	26.426171

CO2_phenMe_TS.log

SCF (wB97x) = SCF
E(SCF)+ZPE(0 K)= -3303.498769
H(298 K)= -3303.445309
G(298 K)= -3303.587718
Lowest Frequency = -233.2098cm-1

Co	2.453125	6.290846	23.294998
K	5.575865	7.562718	21.925863
O	3.128513	8.008331	22.851639
O	3.937000	5.540137	22.373444
O	4.428739	10.075631	21.992299
O	3.373917	6.214112	24.980827
O	6.037786	5.019392	20.937529
O	7.999765	7.002867	20.552731
O	6.869225	9.710476	20.648902

O	5.411739	7.080827	24.560163
N	1.713827	4.583559	23.762411
N	0.995253	7.010044	24.311475
C	4.236115	4.298274	22.261701
C	5.409641	3.952247	21.500615
C	2.805208	9.130198	23.392594
C	3.519799	10.305441	22.976283
C	0.931894	8.224475	24.750401
H	0.156409	8.472103	25.475049
C	3.947641	1.883236	22.688199
H	3.366587	1.086697	23.144879
C	4.560406	6.590788	25.304486
C	1.541577	10.580632	24.934333
H	0.758058	10.678965	25.680495
C	5.822108	2.644022	21.382417
H	6.718398	2.398968	20.823709
C	3.500512	3.225207	22.833726
C	2.276325	11.672497	24.549480
H	2.097641	12.646973	24.989913
C	2.283869	3.442554	23.548444
H	1.803582	2.544636	23.935855
C	5.156703	11.168486	21.459604
H	5.842269	11.570495	22.216623
H	4.473194	11.964518	21.137955
C	1.783578	9.303431	24.360832
C	3.271299	11.531242	23.553884
H	3.836699	12.405432	23.250730
C	7.197676	4.791168	20.155008
H	6.981740	4.076579	19.350440
H	8.003087	4.390513	20.783638
C	5.087774	1.594698	21.985318
H	5.432605	0.572428	21.877705
C	7.663740	9.240345	19.577422
H	8.248159	10.066301	19.145408
H	7.022121	8.832964	18.781713
C	8.627376	8.189305	20.098536
H	9.357569	7.955904	19.311063
H	9.166602	8.592660	20.959660
C	7.617787	6.101796	19.531703
H	8.463792	5.906755	18.857418
H	6.789288	6.506323	18.933555
C	5.920883	10.679310	20.252014
H	5.222545	10.248822	19.519300
H	6.422332	11.540087	19.786411
C	4.855021	6.396783	26.785455
H	5.920663	6.519593	26.983571
H	4.521861	5.411004	27.118642
H	4.298122	7.145229	27.358407
C	0.483176	4.700534	24.452842
C	0.087676	6.010902	24.729739
C	-0.355861	3.642813	24.809174
H	-0.078890	2.618499	24.584558
C	-1.576801	3.872210	25.436816
C	-1.985445	5.198669	25.694510
C	-1.148923	6.249339	25.331475
H	-1.490297	7.265639	25.497813
O	-2.978981	7.952240	22.912729
C	-2.977376	6.776601	22.634638
O	-2.019267	6.134430	21.985920
C	-0.840923	6.884343	21.620272
C	0.254022	5.846313	21.330727
H	-0.555490	7.532374	22.447195
H	-1.079907	7.502621	20.752262
O	1.543500	6.342282	21.580429
H	0.065676	5.012145	22.015789
C	0.158981	5.301857	19.907846
H	-0.836488	4.882720	19.728889
H	0.330487	6.094976	19.171766
H	0.902855	4.515302	19.755548
O	-3.989292	5.984490	22.972192
C	-3.935177	4.584301	22.663066
H	-3.052573	4.127701	23.115950
H	-4.843508	4.164381	23.091920
H	-3.921470	4.429729	21.582698
C	2.104045	7.700307	20.429686
O	3.124337	7.317708	19.958965
O	1.255063	8.534051	20.470803
C	-3.318415	5.478609	26.335776
H	-3.492450	6.552622	26.428709
H	-4.138206	5.054261	25.746334
H	-3.376415	5.034361	27.335462
C	-2.462947	2.713406	25.811942
H	-2.659715	2.701024	26.889356
H	-3.435791	2.777318	25.311600
H	-2.004211	1.760665	25.539056

CO2_phenOMe_TS_t4a.log

SCF (wB97x) = -3454.64181428

Co	2.466994	6.285092	23.309795
K	5.569201	7.591976	21.925294
O	3.129619	8.009734	22.880518
O	3.946168	5.551616	22.367911
O	4.406323	10.093390	22.026658
O	3.400598	6.195224	24.986952
O	6.045568	5.055413	20.924458
O	7.983771	7.060737	20.521209
O	6.829264	9.756792	20.645842
O	5.436043	7.061250	24.553816
N	1.740229	4.568122	23.764429
N	1.011485	6.984509	24.343925
C	4.266398	4.314632	22.267062
C	5.441150	3.982378	21.501453
C	2.797947	9.126115	23.429197
C	3.500178	10.309626	23.016323
C	0.935221	8.196783	24.785696
H	0.155797	8.435953	25.508949
C	4.024618	1.898546	22.719264
H	3.460707	1.095967	23.186715
C	4.593008	6.562122	25.301149
C	1.525647	10.557165	24.981030
H	0.743050	10.644932	25.729473
C	5.876505	2.681003	21.393670
H	6.773315	2.446734	20.831156
C	3.552892	3.233780	22.853369
C	2.250674	11.657389	24.600870
H	2.065013	12.627713	25.047550
C	2.332384	3.435034	23.564127
H	1.870141	2.531907	23.961647
C	5.117139	11.196071	21.491065
H	5.809411	11.599483	22.241182
H	4.422280	11.987971	21.183926
C	1.777441	9.285320	24.400468
C	3.243544	11.530258	23.601664
H	3.800740	12.410728	23.301486
C	7.202037	4.840798	20.132834
H	6.988595	4.121201	19.332075
H	8.018135	4.452759	20.755512
C	5.165469	1.624364	22.012483
H	5.528625	0.607657	21.912881
C	7.609274	9.297406	19.559061
H	8.177771	10.130280	19.119167
H	6.957701	8.885697	18.773709
C	8.592091	8.254618	20.060280
H	9.311033	8.030794	19.259839
H	9.141839	8.660837	20.913376
C	7.599611	6.155905	19.504292
H	8.439671	5.970465	18.819973
H	6.759556	6.551388	18.916293
C	5.868308	10.721442	20.269499
H	5.162922	10.291086	19.543512

H	6.356883	11.589270	19.803276
C	4.906611	6.333892	26.773302
H	4.293390	7.007381	27.380129
H	5.960632	6.527906	26.975840
H	4.653498	5.310228	27.060964
C	0.506698	4.671391	24.448004
C	0.114147	5.974404	24.753432
C	-0.326794	3.591307	24.761436
H	-0.046678	2.584743	24.481906
C	-1.543756	3.812916	25.396060
C	-1.934145	5.134796	25.715946
C	-1.114879	6.197171	25.383013
H	-1.476526	7.195049	25.602831
O	-3.057959	7.769994	22.905887
C	-3.003200	6.596789	22.622817
O	-2.001498	5.997234	21.998179
C	-0.852610	6.800655	21.655331
C	0.278684	5.811906	21.337817
H	-0.590937	7.434925	22.500774
H	-1.112766	7.434673	20.804675
O	1.548058	6.348839	21.602005
H	0.121369	4.952684	21.999232
C	0.201444	5.302379	19.900890
H	-0.777873	4.850481	19.712915
H	0.340264	6.120391	19.185485
H	0.974259	4.548999	19.726655
O	-3.990925	5.763472	22.927777
C	-3.879105	4.369267	22.606738
H	-2.993592	3.937461	23.076657
H	-4.780409	3.912457	23.010950
H	-3.833630	4.227844	21.525345
C	2.076766	7.729964	20.465336
O	3.097295	7.368122	19.978812
O	1.216352	8.550507	20.527735
C	-3.240872	5.050402	27.668995
H	-4.255861	5.302587	27.976073
H	-3.060637	3.983643	27.831995
H	-2.523350	5.643543	28.247189
C	-2.110763	1.500740	25.388706
H	-1.992071	1.371134	24.307131
H	-1.200498	1.176959	25.905015
H	-2.955584	0.907165	25.734234
O	-3.148566	5.378787	26.281861
O	-2.428369	2.846444	25.709354

CO2_phen_TS_2.log

SCF (wB97x) = SCF
E(SCF)+ZPE(0 K)= -3224.932232
H(298 K)= -3224.881902
G(298 K)= -3225.019372
Lowest Frequency = -230.8677cm-1

Co	2.454856	6.295210	23.302943
K	5.574641	7.556701	21.912217
O	3.133873	8.009694	22.855918
O	3.931688	5.537949	22.376673
O	4.434672	10.072356	21.989078
O	3.382634	6.218186	24.983869
O	6.030517	5.005709	20.944068
O	7.987959	6.989087	20.527348
O	6.860390	9.698238	20.622066
O	5.422006	7.073390	24.547724
N	1.712492	4.592603	23.778955
N	1.003031	7.018824	24.323372
C	4.237591	4.296712	22.285953
C	5.411136	3.944587	21.527106
C	2.819461	9.131293	23.401169
C	3.535842	10.304478	22.981254
C	0.950849	8.232651	24.768433
H	0.182023	8.482132	25.499471
C	3.964909	1.887068	22.755639
H	3.389868	1.095021	23.227370
C	4.574311	6.587559	25.298535
C	1.575472	10.583973	24.957918
H	0.798730	10.684158	25.710822
C	5.830897	2.637254	21.431114
H	6.726907	2.387541	20.874021
C	3.509154	3.229128	22.878285
C	2.312488	11.672590	24.569813
H	2.142813	12.646763	25.014394
C	2.291208	3.450900	23.586475
H	1.816452	2.556861	23.989332
C	5.162200	11.162717	21.450262
H	5.857152	11.560662	22.200801
H	4.478936	11.962046	21.136460
C	1.806100	9.306748	24.378293
C	3.298193	11.529290	23.564662
H	3.865096	12.401687	23.259136
C	7.188677	4.771349	20.160459
H	6.972155	4.045235	19.366480
H	7.997751	4.382116	20.791491
C	5.104704	1.593899	22.055111
H	5.455965	0.572204	21.964448
C	7.637627	9.219533	19.541651
H	8.215323	10.041919	19.094089
H	6.983278	8.806065	18.759535
C	8.609074	8.172280	20.055772
H	9.327657	7.933317	19.259410
H	9.160804	8.581864	20.905983
C	7.602136	6.075332	19.519027
H	8.444913	5.874015	18.842614
H	6.769600	6.471183	18.920668
C	5.911792	10.670682	20.234681
H	5.204016	10.242805	19.509570
H	6.411787	11.529297	19.763606
C	4.879351	6.387462	26.776402
H	4.311075	7.119388	27.359223
H	5.943620	6.527118	26.970416
H	4.566128	5.392338	27.101423
C	0.479522	4.710010	24.464397
C	0.088091	6.027224	24.742759
C	-0.361936	3.649872	24.809987
H	-0.095368	2.624950	24.580486
C	-1.577902	3.906807	25.432232
H	-2.230417	3.079634	25.690539
C	-1.974057	5.219599	25.691877
H	-2.938015	5.419523	26.146880
C	-1.148095	6.280166	25.341003
H	-1.488393	7.295731	25.507313
O	-2.994436	7.937229	22.910768
C	-2.993076	6.764908	22.618729
O	-2.024690	6.127220	21.979948
C	-0.844687	6.881964	21.631058
C	0.253141	5.848033	21.339593
H	-0.565943	7.522624	22.466114
H	-1.076726	7.508481	20.767087
O	1.540789	6.348547	21.590439
H	0.068541	5.011515	22.022873
C	0.159658	5.305711	19.915824
H	-0.834046	4.882519	19.736950
H	0.327009	6.100922	19.181087
H	0.906930	4.522800	19.761637
O	-4.013308	5.972873	22.928422
C	-3.965597	4.578422	22.589847
H	-3.107719	4.099884	23.066108
H	-4.893486	4.161847	22.977447
H	-3.915339	4.448672	21.507317
C	2.099278	7.700905	20.439217
O	3.118588	7.317797	19.965949
O	1.252350	8.537102	20.480529

RPO.log

SCF (wB97x) = SCF
E(SCF)+ZPE(0 K)= -192.999177
H(298 K)= -192.993859
G(298 K)= -193.025528
Lowest Frequency = 208.2383cm-1

C	-0.642500	-0.269305	0.020211
C	0.821415	-0.264585	-0.001118
O	0.085494	0.958436	-0.026691
H	-1.153774	-0.499572	-0.914636
H	1.353769	-0.501741	-0.919849
H	1.365093	-0.453606	0.923131
C	-1.422908	-0.544125	1.276297
H	-2.344290	0.045889	1.294242
H	-1.696209	-1.602824	1.323411
H	-0.830988	-0.296688	2.162110

References

1 Deacy, A. C., Moreby, E., Phanopoulos, A. & Williams, C. K. Co(III)/Alkali-Metal(I) Heterodinuclear Catalysts for the Ring-Opening Copolymerization of CO2and Propylene Oxide. *J. Am. Chem. Soc.* **142**, 19150-19160, (2020).

2 Cosier, J. & Glazer, A. M. A Nitrogen-Gas-Stream Cryostat for General X-ray Diffraction Studies. *J. Appl. Crystallogr.*, 105-107, (1986).

3 (Bruker AXS, Madison, WI, 1998, 1998).

4 Sheldrick, G. M. SHELXT - Integrated space-group and crystal-structure determination. *Acta Cryst.* ***A*71**, 3-8, (2015).

5 Sheldrick, G. M. Crystal structure refinement with SHELXL. *Acta Cryst.* ***C*71**, 3-8, (2015).

6 Dolomanov, O. V., Bourhis, L. J., Gildea, R. J., Howard, J. A. K. & Puschmann, H. OLEX2: a complete structure solution, refinement and analysis program. *J. Appl. Crystallogr.* **42**, 339-341, (2009).

7 Gaussian 16 Rev. C.01 (Wallingford, CT, 2016).

8 Deacy, A. C., Phanopoulos, A., Lindeboom, W., Buchard, A. & Williams, C. K. Insights into the Mechanism of Carbon Dioxide and Propylene Oxide Ring-Opening Copolymerization Using a Co(III)/K(I) Heterodinuclear Catalyst. *J. Am. Chem. Soc.* **144**, 17929-17938, (2022).

9 Chai, J. D. & Head-Gordon, M. Systematic optimization of long-range corrected hybrid density functionals. *J. Chem. Phys.* **128**, 084106, (2008).

10 Alipour, M. & Fallahzadeh, P. First principles optimally tuned range-separated density functional theory for prediction of phosphorus–hydrogen spin–spin coupling constants. *Phys. Chem. Chem. Phys.* **18**, 18431-18440, (2016).

11 Glendening, E. D., Landis, C. R. & Weinhold, F. NBO 6.0: Natural bond orbital analysis program. *J. Comput. Chem.* **34**, 1429-1437, (2013).

12 Weigend, F. & Ahlrichs, R. Balanced basis sets of split valence, triple zeta valence and quadruple zeta valence quality for H to Rn: Design and assessment of accuracy. *Phys. Chem. Chem. Phys.* **7**, 3297-3305, (2005).

13 Barone, V. & Cossi, M. Quantum Calculation of Molecular Energies and Energy Gradients in Solution by a Conductor Solvent Model. *J. Phys. Chem. A* **102**, 1995-2001, (1998).

14 Luchini, G., Alegre-Requena, J., Funes-Ardoiz, I. & Paton, R. GoodVibes: automated thermochemistry for heterogeneous computational chemistry data [version 1; peer review: 2 approved with reservations]. *F1000Research* **9**, (2020).

15 Foltran, S., Cloutet, E., Cramail, H. & Tassaing, T. In situ FTIR investigation of the solubility and swelling of model epoxides in supercritical CO2. *J. Supercrit. Fluids* **63**, 52-58, (2012).

16 Svatunek, D. & Houk, K. N. autoDIAS: a python tool for an automated distortion/interaction activation strain analysis. *J. Comput. Chem.* **40**, 2509-2515, (2019).

17 Vermeeren, P., van der Lubbe, S. C. C., Fonseca Guerra, C., Bickelhaupt, F. M. & Hamlin, T. A. Understanding chemical reactivity using the activation strain model. *Nat. Protoc.* **15**, 649-667, (2020).

18 Bickelhaupt, F. M. & Houk, K. N. Analyzing Reaction Rates with the Distortion/Interaction-Activation Strain Model. *Angew. Chem. Int. Ed.* **56**, 10070-10086, (2017).

19 Kong, R. Y. & Crimmin, M. R. Chemoselective C−C σ-Bond Activation of the Most Stable Ring in Biphenylene**. *Angew. Chem. Int. Ed.* **60**, 2619-2623, (2021).
